# Unexpected host dependency of Antarctic Nanohaloarchaeota

**DOI:** 10.1073/pnas.1905179116

**Published:** 2019-06-28

**Authors:** Joshua N. Hamm, Susanne Erdmann, Emiley A. Eloe-Fadrosh, Allegra Angeloni, Ling Zhong, Christopher Brownlee, Timothy J. Williams, Kirston Barton, Shaun Carswell, Martin A. Smith, Sarah Brazendale, Alyce M. Hancock, Michelle A. Allen, Mark J. Raftery, Ricardo Cavicchioli

**Affiliations:** ^a^School of Biotechnology and Biomolecular Sciences, University of New South Wales, Sydney, NSW 2052, Australia;; ^b^Department of Energy, Joint Genome Institute, Walnut Creek, CA 94598;; ^c^Bioanalytical Mass Spectrometry Facility, University of New South Wales, Sydney, NSW 2052, Australia;; ^d^Biological Resources Imaging Laboratory, University of New South Wales, Sydney, NSW 2052, Australia;; ^e^Kinghorn Centre for Clinical Genomics, Garvan Institute of Medical Research, Darlinghurst, NSW 2010, Australia;; ^f^St. Vincent’s Clinical School, University of New South Wales, Sydney, Darlinghurst, NSW 2010, Australia

**Keywords:** archaea, symbiont, DPANN

## Abstract

In hypersaline environments, Nanohaloarchaeota (Diapherotrites, Parvarchaeota, Aenigmarchaeota, Nanoarchaeota, Nanohaloarchaeota [DPANN] superphylum) are thought to be free-living microorganisms. We report cultivation of 2 strains of Antarctic Nanohaloarchaeota and show that they require the haloarchaeon *Halorubrum lacusprofundi* for growth. By performing growth using enrichments and fluorescence-activated cell sorting, we demonstrated successful cultivation of *Candidatus* Nanohaloarchaeum antarcticus, purification of *Ca.* Nha. antarcticus away from other species, and growth and verification of *Ca.* Nha. antarcticus with *Hrr. lacusprofundi*; these findings are analogous to those required for fulfilling Koch’s postulates. We use fluorescent in situ hybridization and transmission electron microscopy to assess cell structures and interactions; metagenomics to characterize enrichment taxa, generate metagenome assembled genomes, and interrogate Antarctic communities; and proteomics to assess metabolic pathways and speculate about the roles of certain proteins. Metagenome analysis indicates the presence of a single species, which is endemic to Antarctic hypersaline systems that support the growth of haloarchaea. The presence of unusually large proteins predicted to function in attachment and invasion of hosts plus the absence of key biosynthetic pathways (e.g., lipids) in metagenome assembled genomes of globally distributed Nanohaloarchaeota indicate that all members of the lineage have evolved as symbionts. Our work expands the range of archaeal symbiotic lifestyles and provides a genetically tractable model system for advancing understanding of the factors controlling microbial symbiotic relationships.

## Significance

We demonstrate that *Candidatus* Nanohaloarchaeum antarcticus requires *Halorubrum lacusprofundi* for growth, illustrating that Nanohaloarchaeota require a host rather than being free living as previously proposed. Developing the means of cultivating Nanohaloarchaeota in the laboratory provides the capacity to advance understanding of how archaea interact and the factors that control their symbiotic relationship (e.g. mutualism, commensalism, antagonism). Our findings amplify the view that Antarctic lakes are a treasure trove for the discovery of microbes with previously unknown properties.

Recent exploration into the genomic landscape of the domain Archaea has shed light on the ecological and evolutionary prominence of uncultivated lineages (1). The Nanohaloarchaea were first identified from metagenomic data as a class of uncultivated halophilic archaea composed of 6 clades (2) and were subsequently placed in the phylum Nanohaloarchaeota within the Diapherotrites, Parvarchaeota, Aenigmarchaeota, Nanoarchaeota, Nanohaloarchaeota (DPANN) superphylum (3). The lineage has since been identified in data from a range of hypersaline environments including: Australian thalassohaline lake (2, 4), Spanish saltern (5), Russian soda brine (6), Californian saltern (7), and Chilean halite (8). From these data, 1 complete metagenome-assembled genome (MAG; *Candidatus* [*Ca.*] Nanopetramus SG9) (8) and 12 incomplete MAGs have been generated. Nanohaloarchaeal cells in environmental samples appear as small cocci with an average diameter of ∼0.6 μm (0.1 to 0.8 μm size range) (2, 4) and a genome size smaller (e.g., 1.1 Mb for *Ca.* Nanopetramus SG9) (8) than haloarchaea (phylum Euryarchaeota, class Halobacteria; ∼3 to 4 Mb).

Despite the monophyly of the DPANN superphylum being called into question (9), members, such as *Candidatus* Nanoarchaeum equitans (0.4-μm diameter, <0.5 Mb) (10), *Candidatus* Nanopusillus acidilobi (<0.3-µm diameter, ∼0.6 Mb) (11), *Candidatus* Parvarchaeum acidophilus (0.6-μm diameter, <1 Mb) (12), and *Candidatus* Mancarchaeum acidophilum (∼1-µm diameter, ∼1 Mb) (13), have all been shown to possess small cell and genome sizes; these are traits that have been proposed to be typical of all DPANN phyla (3). Due to their reduced genomic capacity, these Nanoarchaeota and Pavarchaeota require cell–cell contact with larger (10–12) or similarly (13) sized hosts to proliferate and can be described as ectoparasites (11). The DPANN *Candidatus* Huberarchaeum crystalense has also been described as a possible epibiotic symbiont of *Candidatus* Altiarchaeum sp. (14). Although most DPANN genomes lack some biosynthetic pathways required for autonomous growth, it has been reasoned that certain genetic traits (diversity generating retroelements) may enhance the ability of DPANN lineages (e.g., Pacearchaeota and Woesearchaeota) to have dynamic interactions with their hosts that possibly enable shifts between mutualism, predation, and parasitism (15).

In contrast to host dependence, analyses of an ∼1.24-Mb single amplified genome of *Candidatus* Iainarchaeum andersonii (Diapherotrites) (16) and ∼1.2-Mb metagenome assemblies of *Candidatus* Nanosalina sp. J07AB43 and *Candidatus* Nanosalinarum sp. J07AB56 (Nanohaloarchaea) (2) have drawn the conclusion that these DPANN members could be capable of autonomous growth. The Nanohaloarchaea were predicted to be free living based on their genome size being larger than other known DPANN representatives and nanohaloarchaeal cells not being observed to associate with potential host cells in environmental samples (2). The view that Nanohaloarchaeota may be capable of leading an independent lifestyle is reflected in the current literature (17).

Here, we report the cultivation of Antarctic strains of nanohaloarchaea (proposed as *Candidatus* Nanohaloarchaeum antarcticus) from 2 different hypersaline lakes and show that an Antarctic haloarchaeon, *Halorubrum lacusprofundi*, is required for growth. Examination of the nanohaloarchaea–haloarchaea interactions and analysis of available MAGs of nanohaloarchaea lead us to conclude that Nanohaloarchaeota have evolved as symbionts requiring hosts rather than as free-living cells.

## Cultivation of Antarctic Nanohaloarchaeota

Enrichment cultures were previously established using hypersaline water from Rauer 1 Lake, East Antarctica, resulting in the isolation of *Hrr. lacusprofundi* strain R1S1 (18) (*SI Appendix*, Table S1). Metagenome data of an enrichment culture revealed high relative abundance of *Hrr. lacusprofundi* (47%) and Nanohaloarchaeota (43%) (*SI Appendix*, Fig. S1*A* and Table S1). From guanine-cytosine (GC) binning, iterative assembly, and additional PCR sequencing, 2 scaffolds resolved representing a MAG for Rauer 1 Nanohaloarchaeota, herein referred to as *Ca.* Nha. antarcticus R1 (“Nha-R1”). Transmission electron microscopy (TEM) of the enrichment culture (*SI Appendix*, Fig. S1 *A*–*D*) and 0.22-µm filtrate (*SI Appendix*, Fig. S1 *E* and *F*) revealed small cocci (0.3 to 1.0 µm) resembling the morphology of nanohaloarchaea (2, 4), including small cocci associated with larger, pleomorphic rod-shaped cells characteristic of *Hrr. lacusprofundi* (*SI Appendix*, Fig. S1 *A* and *C*) (18–20).

While Nha-R1 grew in liquid enrichment and on solid medium, multiple attempts to propagate Nha-R1 using the 0.22-µm filtrate in liquid or on solid medium were unsuccessful (*Materials and Methods*). Isolation attempts included the use of spent media from the Nha-R1 enrichment, fresh medium supplemented with a cell extract of the enrichment, and a diffusion chamber inoculated with the Nha-R1 filtrate and placed into a growing enrichment culture. The growth experiments provided nutrients from the enrichment community while preventing Nha-R1 from having physical contact with other species, indicating that cell–cell contact was required for Nha-R1 to proliferate.

To confirm the cultivability of Nanohaloarchaeota and obtain a second MAG, an enrichment culture was established using water from Club Lake, East Antarctica (see Fig. 2). TEM revealed morphologies similar to those from the Rauer 1 Lake enrichment (*SI Appendix*, Fig. S2). After metagenome sequencing (*SI Appendix*, Table S1), a single scaffold was obtained, which had >99% average nucleotide identity (ANI) and an identical 16S ribosomal ribonucleic acid (rRNA) gene sequence to the Nha-R1 MAG, indicating that the enrichments contain strains of the same Nanohaloarchaeota species. The Club Lake Nanohaloarchaeota *Ca*. Nha. antarcticus C is referred to as “Nha-C,” and its metagenome “Nha-Ce” denotes *Ca.* Nha. antarcticus Club Lake enrichment (*SI Appendix*, Table S1). *Hrr. lacusprofundi* (41%) and Nanohaloarchaeota (26%) were the 2 most abundant taxa in the enrichment (*SI Appendix*, Fig. S1*B* and Table S1).

## Growth of Nha-C with *Hrr. lacusprofundi* ACAM34-hmgA

**Fig. 1.**
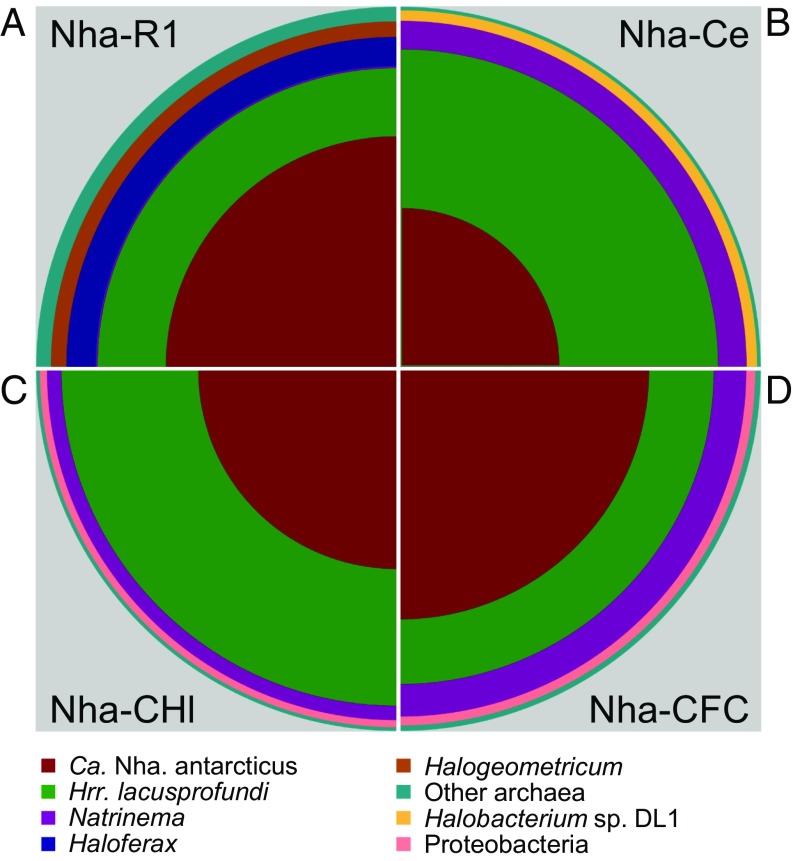
Relative abundances of the major taxa present in *Ca.* Nha. antarcticus enrichment cultures. Metagenomes for Nha-R1 (*A*), Nha-Ce (*B*), Nha-CHl (*C*), and Nha-CFC (*D*). Quadrants show relative abundances of the major taxa identified in each enrichment metagenome. Abundances were calculated using average read depth across a set of universally distributed single-copy genes. Area of the colored sections is directly proportional to the relative abundance of each taxonomic unit. The only taxa identified in all 4 metagenomes were *Ca.* Nha. antarcticus (Nha-R1: 41%; Nha-Ce: 19%; Nha-CHl: 30%; Nha-CFC: 47%), *Hrr. lacusprofundi* (Nha-R1: 28%; Nha-Ce: 59%; Nha-CHl: 56%; Nha-CFC: 29%), and *Natrinema* sp. (Nha-R1: 1%; Nha-Ce: 14%; Nha-CHl: 8%; Nha-CFC: 16%).

**Fig. 2.**
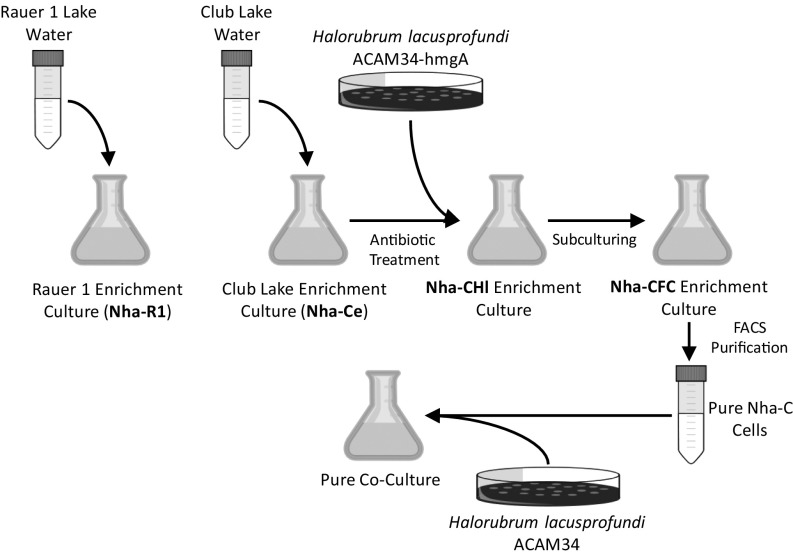
Experimental design for cultivating *Ca.* Nha. antarcticus in enrichments through to coculture with *Hrr. lacusprofundi*. The names of the metagenomes associated with the enrichment cultures (Fig. 1) are in bold: Nha-R1, Nha-Ce, Nha-CHl, and Nha-CFC.

In the enrichments from both lakes, *Hrr. lacusprofundi* was the most abundant species (Fig. 1 *A* and *B*), indicating that it could be the host. The up-regulated 3-hydroxy-3-methylglutaryl coenzyme A reductase gene (*hmgA*) gene in plasmid pJWID1 confers resistance to 2.5 μg mL^−1^ pravastatin in *Hrr. lacusprofundi* ACAM34 (21). Pravastatin inhibits HmgA and hence, lipid synthesis via the mevalonate pathway, which is absent from the *Ca.* Nha. antarcticus MAGs (see below). To inhibit the growth of other archaea in the enrichment, the transformed ACAM34 strain was grown with an aliquot of the Club Lake enrichment in pravastatin (2.5 μg mL^−1^) and bacterial antibiotic (ampicillin 100 µg mL^−1^, chloramphenicol 25 µg mL^−1^, kanamycin 50 µg mL^−1^, tetracycline 10 µg mL^−1^) containing media (Fig. 2). To provide stronger selection pressure for *Hrr. lacusprofundi* ACAM34, after several rounds of growth, pravastatin concentration was increased up to 10 μg mL^−1^, and additional archaeal antibiotics were added (mevinolin 0.02 µg mL^−1^, simvastatin 0.01 µg mL^−1^). This resulted in selection of a strain (ACAM34-hmgA) where the *hmgA* gene from the plasmid (flanked by *Hrr. lacusprofundi* transposases) relocated into the chromosome, and all other plasmid genes were lost or rendered nonfunctional (*SI Appendix*, Fig. S3).

The metagenome of cultures (grown with 10 μg mL^−1^ pravastatin) harvested during midlog phase growth showed a high relative abundance of Nha-C (30%) and *Hrr. lacusprofundi* (56%), with a relatively low abundance of *Natrinema* (8%) and bacterial *Marinobacter* (6%) (*SI Appendix*, Fig. S1*C* and Table S1). The *Ca.* Nha. antarcticus MAG is referred to as “Nha-CHl,” denoting *Ca.* Nha. antarcticus Club Lake *Hrr. lacusprofundi* culture (*SI Appendix*, Table S1).

**Fig. 3.**
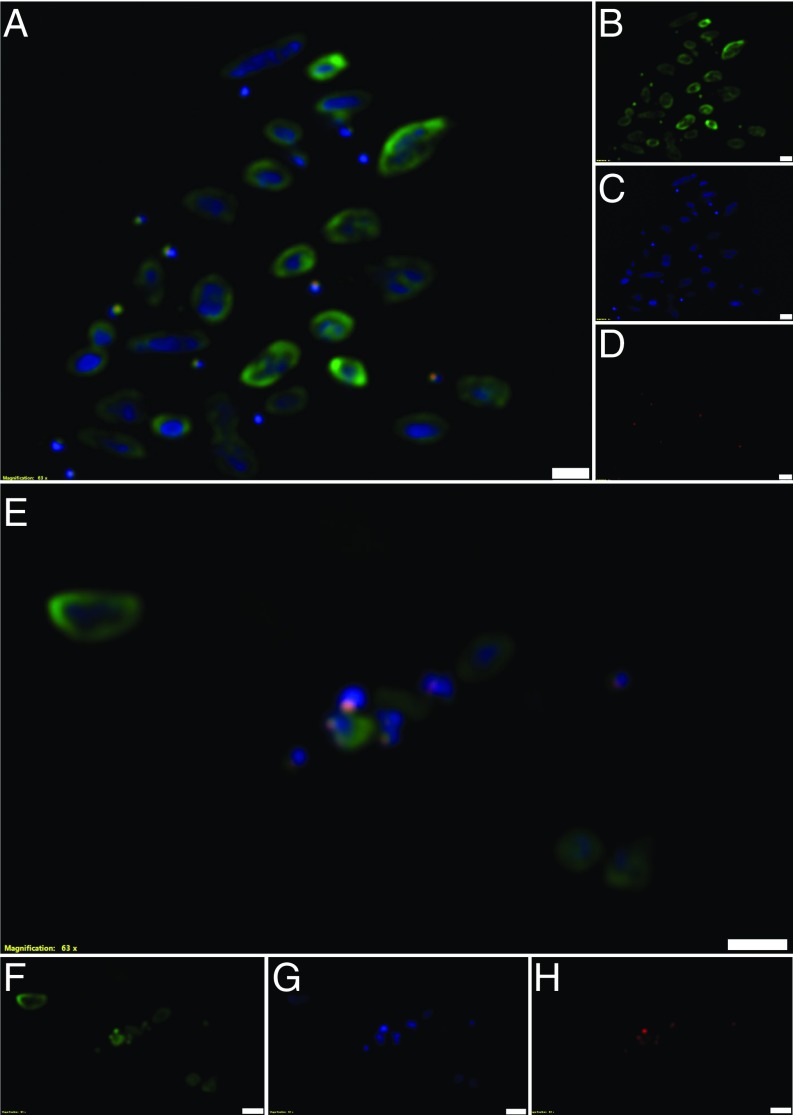
FISH of Nha-C enrichment with *Hrr. lacusprofundi* ACAM34-hmgA. Fluorescence micrographs show individual Nha-C cells among *Hrr. lacusprofundi* cells (*A*–*D*) and Nha-C cells in contact with *Hrr. lacusprofundi* cells (*E*–*H*). Nha-C cells are labeled with a Cy5-conjugated (red fluorescence) probe. *Hrr. lacusprofundi* cells are labeled with a Cy3 (yellow fluorescence; recolored to green to improve contrast) probe; all nucleic acid-containing cells are stained with DAPI (blue fluorescence). Composite image of all 3 filters (*A* and *E*). Individual filters for Cy3 (*B* and *F*), DAPI (*C* and *G*), and Cy5 (*D* and *H*). (Scale bars: 2 µm.)

Using fluorescent in situ hybridization (FISH), *Hrr. lacusprofundi* cells appeared as green pleomorphic rods ∼1 to 3 µm in length (Fig. 3 and *SI Appendix*, Fig. S4). In the same field, coccoid cells <1 µm in diameter fluoresced bright red for the *Ca.* Nha. antarcticus probe (Fig. 3 and *SI Appendix*, Fig. S4). In addition to isolated single cells (Fig. 3*A*), Nha-C cells were frequently observed in contact with Cy3 fluorescing *Hrr. lacusprofundi* cells (Fig. 3*E*). Large cells not fluorescing for either probe but fluorescing blue (DAPI) were other species in the enrichment (e.g., *Natrinema* and bacteria) (*SI Appendix*, Fig. S4*E*). A pure culture of *Natrinema* sp. (previously isolated from the enrichment culture) did not fluoresce for either the Nha-C– or *Hrr. lacusprofundi*-specific probes (*SI Appendix*, Fig. S5), and a pure culture of *Hrr. lacusprofundi* ACAM34 fluoresced for *Hrr. lacusprofundi*-specific probe but not for the Nha-C–specific probe (*SI Appendix*, Fig. S6).

**Fig. 4.**
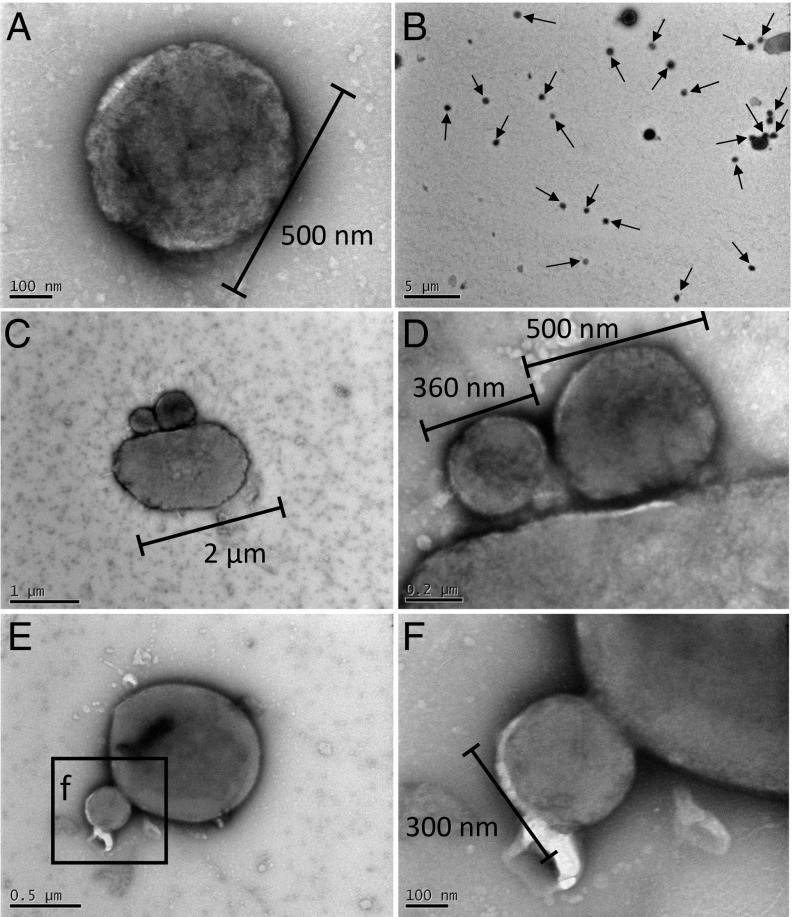
TEM of Nha-C enrichment with *Hrr. lacusprofundi* ACAM34-hmgA. Small individual cells consistent with being Nha-C (*A* and *B*). Arrows mark putative Nha-C cells (*B*). Small cells that appear to have intact membranes with minimal visible boundary layers separating them from larger cells, consistent with Nha-C associating with *Hrr. lacusprofundi* (*C* and *D*). Small cell that appears to be attached with a possible membrane collar to a large cell (*E* and *F*); the morphology is reminiscent of a site of budding (detachment) or possibly, initial stages of fusing after attachment.

TEM images showed the presence of pleomorphic rods and smaller cocci (Fig. 4 and *SI Appendix*, Fig. S7). The small cocci were observed individually (Fig. 4 *A* and *B* and *SI Appendix*, Fig. S7 *A* and *B*) and in contact with larger cells (Fig. 4 *C–F* and *SI Appendix*, Fig. S7 *C*–*F*, *I*, and *J*). Some images revealed the appearance of the cells fused together (Fig. 4 *E* and *F* and *SI Appendix*, Fig. S7 *G* and *H*) and the small cells possibly dividing (*SI Appendix*, Fig. S7 *K*–*N*).

## Nha-C Fluorescence Activated Cell Sorting (FACS) Cells Grow with *Hrr. lacusprofundi*

One enrichment that had a high relative abundance of Nha-C (47%; *Hrr. lacusprofundi*, 29%; *Natrinema*, 16%; other archaea, 6%) (Fig. 1*D* and *SI Appendix*, Table S1) was used for FACS to obtain purified, live Nha-C cells (*SI Appendix*, Figs. S8 and S9); this specific enrichment culture is referred to as “Nha-CFC,” with “FC” denoting use for flow cytometry (Fig. 2). Flow cytometry measurements of cell size were calibrated using size beads (*SI Appendix*, Fig. S8*A*), revealing that a high proportion of the cells were <1 µm in diameter (*SI Appendix*, Fig. S8 *B* and *C*), and all FISH-stained cells in the size range 200 to 800 nm fluoresced for the Nha-C–specific probe (*SI Appendix*, Fig. S8*D*). Consecutive rounds of FACS collected 1,600,000 and 800,000 live cells using gates targeting ∼400 and ∼600 nm in diameter, respectively (*SI Appendix*, Fig. S9).

The only taxa present in all enrichment metagenomes were *Ca.* Nha. antarcticus, *Hrr. lacusprofundi*, and *Natrinema* sp. (Fig. 1 and *SI Appendix*, Fig. S10). Aliquots of the 2 FACS size fractions were inoculated into media only and into cultures of *Hrr. lacusprofundi* ACAM34 or *Natrinema* sp., and the presence of Nha-C was determined by PCR (16S rRNA gene). After 1 mo, Nha-C was detected in an *Hrr. lacusprofundi* culture but was not detected in the media-only incubations (2 replicates) or with *Natrinema* (4 replicates). After an additional 5 d of incubation, all 4 *Hrr. lacusprofundi* replicates were positive for Nha-C. The *Hrr. lacusprofundi* replicate that first gave positive results for Nha-C stopped growing (no change in culture optical density) and was not pursued further. The remaining 3 Nha-C–positive *Hrr. lacusprofundi* replicates were grown with the addition of fresh media, and the presence of Nha-C was assessed at 4 time points starting at early exponential phase (8 d postdilution) and ending at late stationary phase (98 d postdilution). Intensity of PCR products for all 3 replicates varied across the time course, with the strongest band occurring at early exponential phase and gradually declining in band intensity thereafter (*SI Appendix*, Fig. S11). The DNA sequence of the PCR product from early exponential phase matched with 100% nucleotide identity to *Ca.* Nha. antarcticus (100% base call accuracy across ∼1,000 bp). After 130 d, 1 of the media-only replicates had growth of *Hrr. lacusprofundi* and *Natrinema*, while the other replicate had no growth, and none of the media-only or *Natrinema* replicates were positive for *Ca.* Nha. antarcticus.

**Fig. 5.**
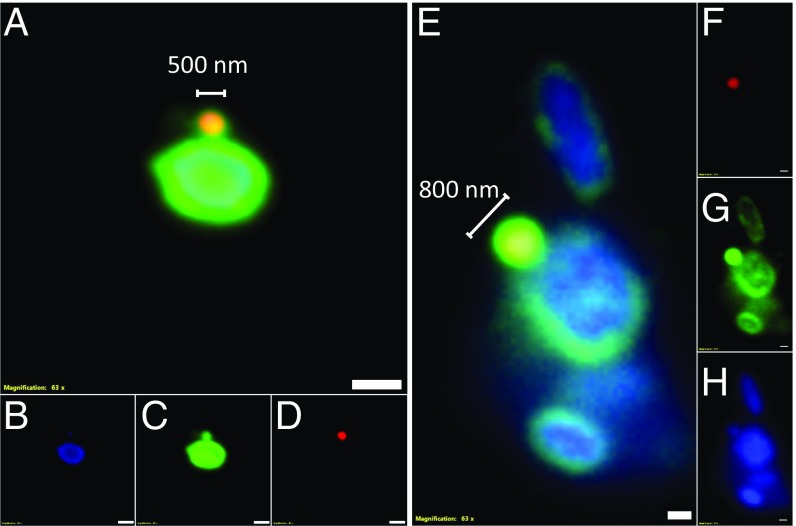
FISH of Nha-C FACS cells with *Hrr. lacusprofundi* ACAM34. Fluorescence micrographs show Nha-C cells in contact with *Hrr. lacusprofundi* cells. The Nha-C cells fluoresced for both the Nha-C and *Hrr. lacusprofundi* probes, indicating that *Hrr. lacusprofundi* rRNA transfers to Nha-C cells. Nha-C cells are labeled with a Cy5-conjugated (red fluorescence) probe. *Hrr. lacusprofundi* cells are labeled with a Cy3 (yellow fluorescence; recolored to green to improve contrast) probe; all nucleic acid-containing cells are stained with DAPI (blue fluorescence). Composite image of all 3 filters (*A* and *E*). Individual filters for Cy3 (*B* and *F*), DAPI (*C* and *G*), and Cy5 (*D* and *H*). (Scale bars: 2 µm.)

**Fig. 6.**
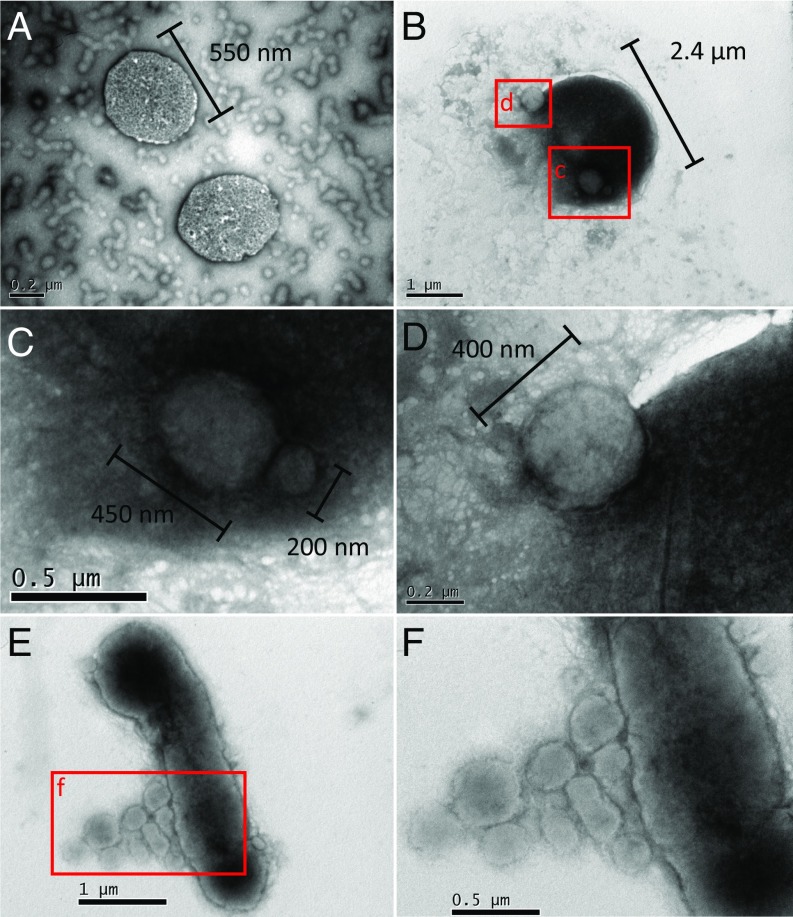
TEM of Nha-C FACS cells with *Hrr. lacusprofundi* ACAM34. Small individual cells consistent with being Nha-C (*A*). A single and “budded” cell (putative Nha-C) intimately associated with a larger cell (putative *Hrr. lacusprofundi*) (*B*–*D*). The budded structure may arise from cell division occurring while Nha-C cells are attached to *Hrr. lacusprofundi* (*D*). Multiple small cells (putative Nha-C) seem to be associated with a larger cell (putative *Hrr. lacusprofundi*) surrounded by possible extracellular material (*E* and *F*). The appearance of extracellular material occurred in other images of FACS cultures (*SI Appendix*, Fig. S14), including in an image of a large cell (putative *Hrr. lacusprofundi*) that appears to have lysed (*SI Appendix*, Fig. S14*E*).

Similar to the enrichments, FISH images of the Nha-C FACS cells grown with *Hrr. lacusprofundi* showed single Nha-C cells (*SI Appendix*, Fig. S12) and Nha-C cells in contact with *Hrr. lacusprofundi* (Fig. 5 and *SI Appendix*, Fig. S12). Consistent with FISH observations, a variety of TEM images showed intimate interactions between small and large cells (Fig. 6 and *SI Appendix*, Fig. S14). Large cells were observed in association with 1 (*SI Appendix*, Fig. S14 *A*, *E*, and *F*), 2 (Fig. 6 *B–D* and *SI Appendix*, Fig. S14 *E* and *F*), or multiple (Fig. 6 *E* and *F*) small cells, similar to observations from enrichment cultures (Fig. 4 and *SI Appendix*, Figs. S1, S2, and S7).

## Characteristics of Nha-C Interactions with *Hrr. lacusprofundi*

Nha-C cells were observed fluorescing for both the Nha-C and *Hrr. lacusprofundi* probes (Fig. 5 and *SI Appendix*, Figs. S12 and S13 *J* and *K*) or just the Nha-C probe (*SI Appendix*, Fig. S13 *B*, *C*, *F*, *G*, *N*, and *O*). Cofluorescence indicates that ribosomal RNA (presumably in ribosomes) transferred from *Hrr. lacusprofundi* to Nha-C, suggesting that direct transfer of cytoplasmic content is a characteristic of their interaction. Similar findings have been reported for DPANN archaea and their hosts, specifically Huberarchaeota (22), Parvarchaeota and Micrarchaeota (previously “ARMAN”) (12, 23, 24), and Nanoarchaeota (25).

The images from across the FACS and enrichment experiments showed cells with minimal visible boundary layers separating them (Fig. 4 *C* and *D* and *SI Appendix*, Figs. S7 *C*–*F* and *I*–*N* and S14 *E* and *F*), cell association possibly involving extracellular material (Fig. 6 *E* and *F* and *SI Appendix*, Figs. S1 *A*–*D* and S2 *E* and *F*), cell fusion suggestive of a shared lipid membrane (Fig. 6 *B–D* and *SI Appendix*, Fig. S7 *G* and *H*), and possible membrane-collar structures associated with budding/fission (Fig. 4 *E* and *F*). In addition, long structures (e.g., 700 × 60 nm) extending outward from small cells were observed by TEM (*SI Appendix*, Fig. S15). Comparable structures were observed using FISH that fluoresced only for the *Hrr. lacusprofundi*-specific probe (*SI Appendix*, Fig. S16), suggesting that they derive from *Hrr. lacusprofundi*. The structures may be the remnants of cytoplasmic bridges, similar to those described for Parvarchaeota and their hosts (12).

Some small cells appeared to have divided while attached to a larger cell (Fig. 6 *B* and *C* and *SI Appendix*, Fig. S7 *K*–*N*), while others were observed apparently divided but unattached to large cells (*SI Appendix*, Fig. S14 *B*–*D*). These images would be consistent with unattached *Ca.* Nha. antarcticus cells achieving cell division before separation from *Hrr. lacusprofundi* (as per Fig. 6 *B* and *C*). It is also possible that gaining sufficient nutrients from *Hrr. lacusprofundi* might enable *Ca.* Nha. antarcticus to perform a limited extent of cell division after host separation.

While *Hrr. lacusprofundi* ACAM34 supported the growth of Nha-C FACS cells, enrichments enabled more stable growth and achieved higher yields of *Ca.* Nha. antarcticus. Enrichments have the capacity to support the growth of a variety of *Hrr. lacusprofundi* strains, although presumably, strain diversity would reduce in enrichments propagated under antibiotic selection with *Hrr. lacusprofundi* ACAM34-hmgA. Enrichments are likely to enable interactions with other species (possibly including viruses) and produce metabolites that alter the physiology of *Hrr. lacusprofundi* and promote the growth of *Ca.* Nha. antarcticus. Known *Hrr. lacusprofundi* strain differences include genome content of integrated viruses, secondary replicons and plasmids, production of plasmid vesicles, and susceptibility to infection by a specific halovirus (18, 26). By performing growth using enrichments and FACS, we demonstrated successful cultivation of Nha-C, purification of Nha-C away from other species, and the growth and verification (16S rRNA gene sequence) of Nha-C with *Hrr. lacusprofundi*; these findings are analogous to those required for fulfilling Koch’s postulates.

## Possible Molecular Mechanisms of Interaction

**Fig. 7.**
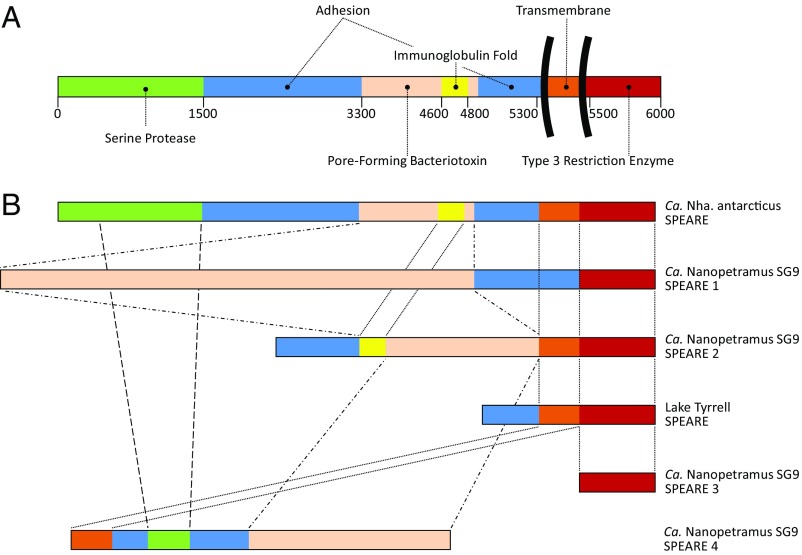
Domain structure of SPEARE proteins. (*A*) Protein domains predicted for the 5,998 amino acid *Ca.* Nha. antarcticus SPEARE protein (IMG gene identification no. 2643306914; locus tag: NAR1_1133). (*B*) Domain structure comparison of SPEARE proteins from Nha-R1, *Ca.* Nanopetramus SG9 (8), and Lake Tyrrell metagenome data (2) depicted approximately to scale. *Ca.* Nanopetramus SG9 SPEARE 1 (8,553 amino acids; hypothetical protein AQV86_04780; accession no. AOV94740.1). *Ca.* Nanopetramus SG9 SPEARE 2 (4,057 amino acids; hypothetical protein AQV86_02335; accession no. AOV95205.1). Lake Tyrrell SPEARE (717 amino acids; IMG gene identification no. LTJ07AB_218510; locus tag: LTJ07AB_218510). *Ca.* Nanopetramus SG9 SPEARE 3 (3,315 amino acids; hypothetical protein AQV86_02330; accession no. AOV94739.1).

The most distinctive feature of nanohaloarchaeal genomes is the existence of genes encoding very long (up to 8,553 amino acids) “SPEARE” proteins, which contain serine protease, adhesion, and restriction endonuclease domains (Fig. 7*A* and *SI Appendix*, Table S2). The *Ca.* Nha. antarcticus SPEARE protein is 5,998-amino acids long, with *Ca.* Nanopetramus SG9 possessing 8,553-, 4,057-, 3,315-, and 655-amino acid versions (Fig. 7*B*). Matches to SPEARE proteins were also identified in 3 incomplete assemblies (∼1 Mbp, PSG99524.1, halite; 27 kbp, PSG99792.1, halite; 9 kbp, RLG14629.1, deep sea hydrothermal vent sediments), and a high identity match was identified in the Lake Tyrrell metagenome that the assemblies derived from (Fig. 7*B*) (2). The *Ca.* Nanopetramus SG9 8553 amino acid protein is the second largest protein described for Archaea, exceeded only by halomucin, a sugar binding protein from *Haloquadratum walsbyi* (∼9,000 amino acids) (27). The 4 *Ca.* Nanopetramus SG9 SPEARE genes combined represent 4% of the genome.

Most of the SPEARE proteins are predicted to have the majority of their length extracytoplasmic, with a transmembrane domain toward the C terminus anchoring the protein to the membrane and the C-terminal region positioned inside the cytoplasm (Fig. 7*A*). The domain structure of the 3,315-amino acid *Ca.* Nanopetramus SG9 protein is reversed, and the transmembrane domain is toward the N terminus (Fig. 7*B*).

The most conserved region of the SPEARE proteins is the intracellular portion, which contains domains characteristic of type III restriction enzymes, suggesting a role in DNA degradation. Similar sequences were identified in assemblies of the DPANN lineages Woesearchaeota, Aenigmarchaeota, and Micrarchaeota as well as Altiarchaeales (*SI Appendix*, Fig. S17 and Table S3) but not in Nanoarchaeota or Parvarchaeota.

The extracytoplasmic portions of the SPEARE proteins display more sequence variation, but all extracytoplasmic portions possess at least 1 laminin-G domain and multiple other adhesion domains (Fig. 7*B* and *SI Appendix*, Table S2). The *Ca.* Nha. antarcticus protein contains distinct serine protease (amino acids 1 to 1,500) and bacteriotoxin (3,300 to 4,800) domains (Fig. 7*A*). Tertiary structure of the toxin domain is predicted to be similar to the bacterial BC toxin subunit of the ABC toxin complex that mediates formation of pores in the membrane of targeted cells (28). The highest identity (25 to 35%) was to predicted adhesion proteins from several bacterial genomes. Notably, many of the bacterial lineages (*Bacteriovorax*, Elusimicrobia, members of the Candidate Phyla Radiation) are characterized by or predicted to have lifestyles in which they attach to and parasitize other organisms (29–31).

Proteomic analysis demonstrated that the Nha-C SPEARE protein was synthesized (*SI Appendix*, Table S4). The protein was most abundant in polyacrylamide gel fractions >315 kDa, with mass spectrometry (MS) analysis detecting 81 unique peptides (18% sequence coverage) of the ∼550-kDa extracytoplasmic portion of the protein (*SI Appendix*, Fig. S18). Peptides to the transmembrane and cytoplasmic portions were identified only in size fractions <315 kDa (*SI Appendix*, Fig. S18). The presence of the cytoplasmic and transmembrane-extracytoplasmic portions in distinct size ranges may be the result of posttranslational processing or the protein preparation protocol (bead beating).

Knowing that the protein is synthesized and remains largely (or fully) intact prompts speculation about its possible function. The adhesion domains could facilitate physical interactions, with the host’s S layer degraded through serine protease activity. The anchoring domain may help position the bacterial toxin-like domain correctly to form a pore that enables transport in and/or out of the cytoplasm. Transfer of genetic material into the nanohaloarchaeal cell could be degraded by the intracellular restriction enzyme domain. Such activity may serve to reduce possible negative impacts of taking up DNA (e.g., mutation via recombination) and/or provide a source of nucleotides for replication and energy conservation.

In addition to the SPEARE protein, proteins annotated as subunits FlaI, FlaJ, and archaellin were identified in the Nha-C proteome (*SI Appendix*, Table S4). By themselves, these subunits are not sufficient to form a functional archaellum, and the other requisite subunits are not present in the *Ca.* Nha. antarcticus MAG. The genes are homologous to type IV pilus proteins (PilB, PilC, and pilin), and adhesion domains (immunoglobulin folds and polycystic kidney disease domains) are present in the “archaellum.” Other DPANN lineages (Woesearchaeota and Diapherotrites) also possess these putative pilus-like structures, indicating that they may play a role in mediating the interactions between DPANN archaea and their hosts.

## Metabolic and Cellular Capacity of Nanohaloarchaeota

Strong evidence for Nanohaloarchaeota host dependency relates to the absence of key biosynthetic pathways for essential compounds, such as lipids, amino acids, nucleotides, and cofactors (*SI Appendix*, *SI Text*). The mevalonate pathway genes for lipid precursor synthesis are conserved and recognizable in Archaea (32), and therefore, their absence is strong evidence that Nanohaloarchaeota are incapable of synthesizing phospholipids. The genomic and proteomic data are indicative of *Ca.* Nha. antarcticus having an aerotolerant, heterotrophic, fermentative metabolism as inferred previously for Nanohaloarchaeota (2), and a total of 458 proteins were detected in the proteome (38% of protein-encoding genes) (*SI Appendix*, Table S4). Overall, the proteome is consistent with the genomic evidence that *Ca.* Nha. antarcticus can synthesize its own carbohydrates (including for storage) but otherwise, has extremely limited biosynthetic capabilities (*SI Appendix*, *SI Text*). Thus, *Ca.* Nha. antarcticus would require certain biopolymers from its host, including polypeptides and nucleic acids as sources of amino acids and nucleotides, respectively. Conceivably, *Ca.* Nha. antarcticus could gain access to essential metabolic products when it is attached to hosts, with surplus carbon stored as polysaccharides that could be mobilized when cells are nonattached. It is possible that *Hrr. lacusprofundi* may gain some benefit from by-products produced by *Ca.* Nha. antarcticus that the latter apparently cannot assimilate, such as ammonium or acetate (33, 34).

## Genome Variation and Evolution of Nanohaloarchaeota

Metagenome read and contig mapping identified Nanohaloarchaeota in 2 Vestfold Hills lakes (Deep Lake and Club Lake) and 4 Rauer Island lakes (3, 6, 11, 13) (*SI Appendix*, Figs. S19–S21). The samples from Deep Lake represented 37 metagenomes spanning 8 y (December 2006 to January 2015) and covering a seasonal cycle (December 2013 to January 2015) and depth profile (2008: surface and 5, 13, 24, and 36 m) (26). Read coverage across the Deep Lake metagenomes was ∼0.5%, similar to Club Lake, with the Rauer Island lakes having more variable coverage, and highest overall coverage (3.5%) was from Rauer 3 Lake (*SI Appendix*, Fig. S19). All of the lakes with Nanohaloarchaeota contained high levels of haloarchaea, with the highest representation in the Vestfold Hills lakes (26).

Contig and read coverage were high (average genome coverage 68%), although the relative abundance of Nanohaloarchaeota was low (≤3.5%), and contigs had high identity (>99%), illustrating that little genomic variation existed within the nanohaloarchaeal populations (*SI Appendix*, Figs. S19–S21). Short regions existed with high read depth (*SI Appendix*, Figs. S20 *D*–*G* and S21) and contained mobile elements (e.g., transposases) with high identity to Antarctic haloarchaea (e.g., 100% match to Halorubraceae IS5-like element) (*SI Appendix*, Table S5), indicating that they arose through sharing with haloarchaea. Gaps between contigs (∼270, 930, and 1,025 kb) coincided with low read recruitment (*SI Appendix*, Figs. S20 *D*–*G* and S21) and contained similar types of sequences (transposases, viral genes, hypothetical proteins), most of which had best matches to haloarchaea. Of note was a complete type 5 Bacteriophage Exclusion (BREX) system that was absent in Nha-CHl but present in Nha-R1 (*SI Appendix*, Fig. S20*C* and Table S5). BREX functions by inhibiting viral replication (35). One additional gene was present (2643307549) downstream from the BREX genes, which is annotated as conserved Nanohaloarchaeota hypothetical protein and may possibly be a Nanohaloarchaeota-specific BREX gene. The BREX cluster was flanked by a gene annotated as a viral integrase (2643307539 integrated microbial genomes [IMG] gene identification), with average coverage indicating that the BREX cluster itself may be part of a mobile element. The read depth coverage across all lake metagenomes was ∼25% of average, indicating that the BREX-containing phylotype was present throughout all of the lakes in approximately 1/4 of the Nanohaloarchaeota population.

While genomic variation across the Antarctic Nanohaloarchaeota is minimal, the genomes of non-Antarctic Nanohaloarchaeota have much lower similarity (≤70% ANI, <90% 16S rRNA gene identity) (*SI Appendix*, Fig. S22), indicating that the other known nanohaloarchaeal lineages represent distinct genera from a range of diverse types of hypersaline environments. This level of diversity (i.e., genera) is similar to the diversity present within haloarchaea (36) and may arise within the Nanohaloarchaeota as a result of host specificity. If coevolution occurs, each lineage of Nanohaloarchaeota would be expected to interact with a specific lineage of haloarchaea. In this regard, we predict that a host for some of the Lake Tyrrell Nanohaloarchaeota is *Hqr. walsbyi*, as it is an abundant species of haloarchaea in the lake, and nanohaloarchaea appear in FISH images associated with square-shaped cells (figure 2 in ref. 2).

## Concluding Remarks

Cultivation of *Ca.* Nha. antarcticus with its host *Hrr. lacusprofundi* represents successful laboratory cultivation of a member of the Nanohaloarchaeota. In defining a host, the study corrects the view that Nanohaloarchaeota are likely to be free living and have a nonhost-associated lifestyle (2). We propose that members of the haloarchaea, which are present in all environments in which Nanohaloarchaeota have been identified, are the hosts. The phylogenetic diversity of Nanohaloarchaeota seems to be equivalent to that of the haloarchaea (2). The existence of distinct clades of Nanohaloarchaeota may reflect specialization to hosts from specific clades of haloarchaea.

The main species of haloarchaea in Deep Lake are known to support a high level of intergenera gene exchange that is counterbalanced by niche adaptation, thereby maintaining sympatric speciation (36, 37). In searching for mechanisms of gene exchange between the dominant Antarctic species, *Hrr. lacusprofundi* R1S1 was found to harbor a plasmid (pR1SE) that packages into specialized membrane vesicles and disseminates like a virus (18). Identifying *Hrr. lacusprofundi* as a host for *Ca.* Nha. antarcticus adds to the known physiological capacity of this species and suggests that it is particularly receptive to internal (pR1SE) and external (*Ca.* Nha. antarcticus) stimuli that invoke major changes to its membrane. Other than *Ca.* Nha. antarcticus, the cultivated DPANN superphyla are the hyperthermophilic *Ca.* Nanoarchaeum equitans (10), *Ca.* Nanopusillus acidilobi (11), and *Ca.* Nanoclepta minutus (38) and the acid mine drainage *Ca.* Micrarchaeota (ARMAN-1) A_DKE (17, 39) and *Ca.* Mancarchaeum acidophilum, which is no longer cultivatable (13). The *Hrr. lacusprofundi*–*Ca.* Nha. antarcticus model provides a useful experimental system that includes the ability to genetically manipulate *Hrr. lacusprofundi* (20) for advancing understanding of how archaea interact and the factors that control their symbiotic relationship (e.g., mutualism, commensalism, antagonism).

## Materials and Methods

### Cultivation of Nanohaloarchaeota.

Enrichment cultures were grown using water collected from Rauer 1 Lake, Filla Island, Rauer Island group, East Antarctica (−68.8081667, 77.8550500) in September 2014 and Club Lake, Vestfold Hills, East Antarctica (−68.5417333, 78.2467667) in November 2014. Descriptions of the systems, metagenomes, and/or isolation of haloarchaea from these lakes were previously described (18, 26). Lake water was inoculated into a range of concentrations of modified David Burns cultivation media 2 (DBCM2) medium [DBCM2 (40) supplemented with 1 g L^−1^ peptone and 0.1 g L^−1^ yeast extract] or artificial Deep Lake vitamin broth (ADLVB) (19) in glass flasks shaken at 100 to 120 rpm at 16 °C to 20 °C, and the cultures were monitored for the presence of small cells using TEM (see below). From MAGs of Nha-R1 (see below), specific primer sets were designed (*SI Appendix*, Fig. S23 and Table S6, primer pairs SB1, SB2, and NHA-FISH). To monitor the community composition of enrichment cultures, PCR sequencing was performed using these primers plus 16S rRNA gene primers specific to Bacteria (*SI Appendix*, Table S6, primer pair Bacterial 16S) and Archaea (*SI Appendix*, Table S6, primer pair Archaeal 16S) (41). Subculturing the enrichment culture onto ADLVB agar plates (16.5 g L^−1^ agar, 20 °C) produced single colonies, which were subsequently inoculated into ADLVB (10 mL), grown at 100 rpm at 16 °C for 1 wk, and then, transferred into 40 mL ADLVB medium, and growth was continued.

To attempt to purify Nha-R1 away from other species, a culture was filtered through a 0.22-μm pore filter (Corning Filter Systems), and the filtrate was examined by TEM to assess the presence of *Ca.* Nha. antarcticus-sized cells. Fresh ADLVB media (30 mL) were inoculated with the filtrate (10 mL) and grown for 4 wk (100 rpm, 16 °C). To provide Nha-R1 with the compounds present in the enrichment culture, spent media were obtained by sequential filtration through 0.8-, 0.22-, and 0.1-μm filters. Spent media (30 mL) were inoculated with Nha-R1 inoculum (10 mL) and grown for 4 wk (16 °C, 100 rpm). Cell extract supplemented media were used to provide Nha-R1 with any compounds present in the enrichment culture that would not pass through the filtration steps used for producing spent media. Cell extracts from the enrichment culture, a pure *Hrr. lacusprofundi* ACAM34 culture, and a pure culture of a halophilic Antarctic bacterium *Halomonas* sp. isolated from the enrichment culture were obtained by sonication or autoclaving. Cells harvested by centrifugation at 6,800 × g for 30 min were sonicated using a Q500 Sonicator (Qsonica) at an output intensity of 30% with 3 bursts each of 1 min, or pellets were resuspended in media and autoclaved at 121 °C for 20 min. For growth experiments, cell extracts were added to stock ADLVB media to a concentration proportional to the volume of the culture from which the extract was acquired (i.e., 100 mL of cell extract was harvested from 1 L of culture, and therefore, 10 mL of cell extract was added for every 90 mL of stock media). Cultures were inoculated with the Nha-R1–enriched inoculum and grown for 3 wk (40 mL, 100 rpm, 16 °C). Diffusion chambers were used to attempt to accurately emulate nutrient flux and provide an ongoing supply of nutrients to Nha-R1. Diffusion chambers were made from Vivaspin 20 columns (1 million-molecular weight cutoff; Sartorius Stedim) or from a 14-kDa dialysis tube (Millipore Sigma). Both types of chambers were inoculated with 10 mL of 0.22 µm filtered culture, with an additional 10 mL of fresh ADLVB media added to the dialysis tubing or 40 mL of fresh ADLVB added to the Vivaspin unit, and the chambers were submerged in the enrichment culture and left to grow for 3 wk (100 rpm, 16 °C). All growth experiments were performed at least 3 times each. To obtain a pure culture of the *Natrinema* sp. present in the enrichment, a selective medium was designed using the inferred capacity of this *Natrinema* sp. to utilize gluconate as a sole carbon source and nitrite as a sole nitrogen source. Growth of the Nha-R1 enrichment in *Natrinema* isolation media [3.1 M NaCl, 813 mM MgCl_2_ 6H_2_O, 62 mM MgSO_4_ 7H_2_O, 50 mM KCl, 510 nM gluconate, 1.5 µM NaNO_2_, 100 mM CaCl_2_, 0.01× Vitamin Mix (40)] yielded a pure culture of the *Natrinema* sp. that was then used for additional growth experiments as a potential host.

### Growth of *Ca.* Nha. antarcticus with *Hrr. lacusprofundi* ACAM34.

*Hrr. lacusprofundi* ACAM34 was transformed with plasmid pJWID1 as described previously (21) and grown to an optical density (OD)_600_ of 1.0 in modified DBCM2 medium. The *Hrr. lacusprofundi* ACAM34 pJWID1 and Nha-Ce enrichment cultures were centrifuged at 8,000 × *g* for 20 min, and pellets were resuspended and combined in 1 mL total of fresh DBCM2 media. The mixture was incubated in a 2-mL microfuge tube at room temperature for 2 h and then used to inoculate 4 flasks of modified DBCM2 media containing 2.5 µg mL^−1^ of pravastatin and bacterial antibiotics (ampicillin 100 µg mL^−1^, chloramphenicol 25 µg mL^−1^, kanamycin 50 µg mL^−1^, tetracycline 10 µg mL^−1^). Cultivation of the enrichment, referred to as Nha-CHl, was continued by diluting cultures when they had reached OD_600_ of 1.0 to 0.05 using fresh modified DBCM2 medium supplemented with 10 µg mL^−1^ pravastatin, 0.02 µg mL^−1^ mevinolin, 0.01 µg mL^−1^ simvastatin, and bacterial antibiotics as described previously (100 mL, 100 rpm, 22 °C). Treatment of the enrichment with this combination of antibiotics resulted in selection of an *Hrr. lacusprofundi* strain in which pJWID1 had integrated into the genome, losing all genes with the exception of the up-regulated *hmgA* gene; the strain is referred to as *Hrr. lacusprofundi* ACAM34-hmgA. Cultures were incubated for prolonged periods (up to 3 mo) and routinely assessed for taxonomic composition, and *Ca.* Nha. antarcticus abundance was evaluated using PCR and microscopy. Microscopy of a replicate of the Nha-CHl culture that had high relative abundance of *Ca.* Nha. antarcticus was subjected to metgenome sequencing, confirming its abundance. This culture is referred to as Nha-CFC and was used for FACS experiments.

### Transmission Electron Microscopy.

TEM was performed as described previously (18). Samples were diluted to OD_600_ of 0.2 to 0.5, fixed with 2.5% glutaraldehyde, incubated overnight at 4 °C, mounted onto formvar-coated copper grids, negatively stained with uranyl acetate (2%) for 2 min, and dried overnight. Imaging was performed at 100 kV using a JEOL JEM-1400 electron microscope at the Electron Microscopy Unit at the University of New South Wales, Sydney, Australia.

### Fluorescence Microscopy.

The 16S rRNA probes conjugated with fluorophores were designed using ARB (42), screened for specificity using ProbeCheck (43), and synthesized by Integrated DNA Technologies Inc. *Ca.* Nha. antarcticus probes were conjugated with Cy5, and *Hrr. lacusprofundi* was conjugated with Cy3 (*SI Appendix*, Fig. S23 and Table S6). Samples were fixed with 2.5% glutaraldehyde, incubated overnight at 4 °C, and dehydrated using an ethanol series (50, 80, 100% [vol/vol]; 3 min for each concentration). Hybridization conditions were optimized at 46 °C for 3 h as previously described (44). Samples were counterstained with DAPI (ThermoFisher Scientific) as per the manufacturer’s instructions. Samples were mounted onto glass slides along with SlowFade Gold antifade reagent (ThermoFisher Scientific). Images of the emission spectrum of each dye were acquired separately in grayscale at 100× magnification on an Olympus BX61 microscope using cellSens v2.2 software (Olympus Corporation). Images were assigned color postacquisition to maximize contrast, and images were combined to form composite images of all 3 dyes for each field of view.

### Flow Cytometry and Cell Growth.

Sorting was performed on a BD Influx Cell Sorter (BD). Software used for sorting was the BD FACS Sortware version 1.2.0.117, Valcomp 7.5.1.3.16, on a Utopex server, build 1.2.0.107. Sorting was done into a 2-tube holder and was carried out using a piezo amplitude of 0.18 with a drop delay of 45.8111. Mode used was 1.5 Drop Pure with a drop envelope of 1.5 drops and an objective of purify. The phase mask was 16/16, with extra coincidence bits of 4 and a drop frequency of 38.40 kHz. Size regions for sorting were established using 4,000 series monosized particles of sizes 300 nm, 500 nm, 800 nm, and 1 µm (ThermoFisher Scientific). Two gates for sorting were drawn around regions representing ∼400 and ∼600 nm, respectively. A second gate was drawn using fluorescence for Cy5, with an unstained sample used as a negative control and FISH-stained cells used as a positive control. Cell sizes were estimated by summing the total number of cells recorded in each size range equating to the standard size beads and taking the average across all 3 size ranges that were expected to capture Nha-C cells (300, 500, and 800 nm), resulting in an average cell size estimate of 340 nm in diameter. The average cell size for Nha-C cells from TEM was calculated from the diameter of 30 cells across a number of fields of view, including both attached and nonattached cells, resulting in an average cell size estimate of 400 nm. Based on these estimates, there is minimal difference (60 nm) between flow cytometry and TEM estimates of cell size.

Sorted Nha-C cells (500 µL) from each size range (∼400 and ∼600 nm in diameter), equating to ∼320,000 and 160,000 cells, respectively, were each added to 500 µL (∼5 × 10^6^ cells) of *Hrr. lacusprofundi* ACAM34 or *Natrinema* sp. or 500 µL of fresh DBCM2 media. A total of 4 replicates, 2 for each size range used in sorting (i.e., total of 8), were performed for each incubation of Nha-C with *Hrr. lacusprofundi* ACAM34 or *Natrinema* sp., and 2 replicates were performed for Nha-C with fresh media. All cultures were incubated for 1 d static at 18 °C before fresh media were added to bring cultures to a final volume of 10 mL. Cultures were incubated at 18 °C shaking at 100 rpm and monitored for up to 130 d.

### Metagenomes and MAGs of Enrichment Cultures.

Enrichment culture biomass was harvested by centrifugation at 5,000 × *g*, and DNA was isolated using a QIAGEN DNeasy Blood and Tissue Kit following the manufacturer’s instructions. To obtain the Nha-R1 MAG, DNA was sheared to 300 bp using the Covaris LE220 and size selected using SPRI beads (Beckman Coulter). The fragments were treated with end repair, A tailing, and ligation of Illumina compatible adapters (IDT, Inc.) using the KAPA-Illumina library creation kit (KAPA Biosystems). qPCR was used to determine the concentration of the libraries before sequencing on the Illumina HiSeq-2500 to yield 150-bp paired end reads at the Department of Energy (DOE) Joint Genome Institute. Quality-filtered metagenomic sequences were assembled with Megahit (version 1.0.6) (45). Assembly of the enrichment culture metagenome yielded 28,529 scaffolds (maximum length = 136,362 bp, scaffold N50 = 53,057 bp), which derived from 2 main taxa: *Hrr. lacusprofundi* 670-fold average read depth (28% relative abundance) and Nanohaloarchaeota 983-fold average read depth (41% relative abundance). GC binning identified reads belonging to a low-GC (40%) archaeon, which resolved after iterative assembly using SPAdes (version 3.5.0) into 3 scaffolds representing a single incomplete MAG for Nha-R1, and contigs were uploaded and annotated by the IMG pipeline (46). To close gaps in the Nha-R1 MAG, contigs from the Rauer 1 Lake enrichment metagenome (IMG genome identification no. 3300005925) were aligned to the 3 Nha-R1 scaffolds using Burrows–Wheeler Aligner (BWA) (47) and manually assessed to identify overlapping sequences. A metagenomic contig was identified that aligned to both scaffolds 2 and 3, indicating that the 2 could be concatenated. PCR primers were designed (SB1 and SB2 in *SI Appendix*, Table S6), and PCR amplification of the region was performed. Sanger sequencing of the product confirmed that scaffolds 2 and 3 could be concatenated, resulting in gap closure and removal of ∼5.8 kbp of sequence from scaffold 2.

To obtain MAGs for Nha-C, Nanopore sequencing was performed according to the manufacturer’s instructions on a GridION release v17.11.4 with the following alterations. The SQK-LSK108 1D Genomic DNA by ligation kit was used to prepare 1.4 µg of DNA from the Club Lake enrichment (Nha-Ce) and 2.3 µg of DNA from the Club Lake enrichment grown with *Hrr. lacusprofundi* ACAM34 (Nha-CHl). The formalin-fixed, paraffin embedded (FFPE) repair and end prep steps were combined into a single reaction with the following components: DNA in 48 μL water, 3.5 μL FFPE DNA Repair Buffer, 2 µL NEBNext FFPE DNA repair mix, 3.5 µL Ultra II End-prep reaction buffer, and 3 µL Ultra II End-prep enzyme mix. The reaction was incubated at 20 °C for 5 min and 65 °C for 5 min. After end repair, the DNA was purified by adding Ampure XP beads at a 1:1 ratio (Beckman Coulter A63880) and rotating on a Hula mixer for 5 min. The beads were pelleted on a magnet, washed twice with 200 μL of fresh 70% ethanol, and resuspended in 31 µL of nuclease-free water. After the final elution, 408 or 660 ng of adapted DNA was loaded onto 2 FLO-MIN106 flow cells with 1,407 and 1,560 pores each for samples Nha-Ce and Nha-CHl, respectively. The sequencing ran for a total of 48 h for each run. The most up to date Oxford Nanopore software available at the time of the run was used. For Nha-Ce, MinKNOW acquisition software version 1.10.24 and Guppy base calling software version 0.3.0 were used. For Nha-CHl, MinKNOW version 1.13.5 and Guppy version 1.4.3 were used.

De novo assembly was carried out on the Nanopore data using a combination of miniasm version 0.2-r168-dirty (parameter flags: -f), minimap version 2.9 (parameter flags: -Sw5 -L100 -m0 -t16) (48), and racon version 1.2.0 as described previously (49). Contigs were uploaded and annotated using the IMG pipeline. Taxonomic classification was assigned based on best basic local alignment search tool (BLAST) hit, interpreted through the MEGAN 6.12.0 software, and manually reviewed for accuracy. Raw reads were aligned to the polished contigs using the BWA-mem algorithm, and coverage data were calculated using the pileup.sh script within the bbmap package. Relative abundances were calculated by taking the average fold coverage across a set of universally distributed single-copy genes (50). Genes were manually identified using IMG annotations, and taxonomy was assigned based on best blast hit. Raw reads were aligned, and coverage was calculated as above. Abundances were averaged across all genes identified for each taxonomic unit. Metagenome statistics were calculated using the stats.sh script from the bbmap package. *Ca.* Nha. antarcticus contigs in the Nha-Ce and Nha-CHl metagenomes were identified by aligning all contigs against the reference Nha-R1 MAG using BWA-mem. Identified Nha-Ce and Nha-CHl contigs were aligned to each other to establish a consensus sequence and determine the level of heterogeneity in the Nha-Ce and Nha-CHl metagenomes. This process yielded a single contiguous sequence representing the complete genome of *Ca.* Nha. antarcticus from the Nha-CHl metagenome, referred to as the Nha-CHl MAG.

The Nha-R1 MAG contained the 53 housekeeping genes identified as signature sequences for archaeal genomes (2). Metabolic pathways were reconstructed using IMG and manual analysis. Hypothetical proteins and the SPEARE protein were manually annotated as described previously (51). Sequence similarity to known proteins was assessed using BLAST (52), conserved domains using InterProScan (53), signal peptides using SignalP (54), prediction of cellular location using TMHMM (55), protein alignments by HMM–HMM (hidden Markov model) comparison using HHpred (56), and tertiary structure prediction using Swiss Model (57). In addition for the SPEARE protein, phylogenetic tree construction was performed using the maximum likelihood method based on the Jones Taylor Thornton (JTT) matrix-based model (58) using MEGA7 (59). Tertiary structure and function prediction was also performed using I-TASSER (60) with a 1,500-amino acid sliding window. Potential functional domains within the SPEARE protein were subjected to small-scale structural modeling using I-TASSER (60).

For contig recruitment to replicons, contigs ≥1 kb from each metagenome were compared with the Nha-Ce contig (Ga0309993_1030) using nucmer from the MUMMER 3 toolkit (61). Only hits spanning at least 5 kb and with ≥80% nucleotide identity were considered. The percentage of genome covered by metagenome contigs was calculated based on the hits identified by nucmer cumulated over the entire genome. The corresponding read coverage was calculated by summing the number of reads mapped to all contigs with a nucmer hit to the replicon and expressed as a percentage of the total number of reads mapped to all contigs. In addition, unassembled reads for each lake sampling time point were pooled together and mapped to the Nha-Ce contig (Ga0309993_1030) using Bowtie2 (62). The resulting alignments were then analyzed using Samtools (63) to generate total read mapping and read depth values using the idxstats and depth options, respectively. Read depth was plotted for each pooled set of reads using a 5-kb moving average. ANI between the 6 nanohaloarchaeal genomes (Nha-R1, Nha-CHl, *Ca.* Nanopetramus, *Ca.* Nanosalina, *Ca.* Nanosalinarum, and *Ca.* Haloredivivus) was calculated using the JSpeciesWS ANIb tool (64).

### Proteomics.

Proteomics was performed based on methods previously used for the analysis of proteins from Antarctic haloarchaea (18, 20). Biomass for proteomics was collected from an Nha-CHl enrichment culture in which *Ca.* Nha. antarcticus appeared (by microscopy) to be the most abundant taxon. Cells were centrifuged at 20,000 × *g* for 30 min and resuspended in 400 μL of radioimmunoprecipitation assay buffer containing 2 μL proteinase inhibitor mixtures (Sigma) and 2 scoops of silica beads (BioSpec, 0.5 mm). Bead beating was performed for 30 s at 5,000 rpm and repeated 4 times. The sample was maintained on ice between pulses. After the bead beating, the sample was incubated at 37 °C for 5 min followed by centrifugation at 19,083 × *g* at 4 °C for 10 min. The supernatant was transferred to a new tube, and the protein concentration was measured using a 2-D Quant kit (GE Healthcare) as per the manufacturer’s instructions. For in-solution digestion of proteins, ∼40 μg of protein was reduced in 5 mM dithiothreitol (DTT) at 37 °C for 30 min, alkylated in 10 mM iodoacetic acid at room temperature for 30 min, and digested with trypsin (1 μg; sequencing grade; Promega) overnight. The digest (5 μg) was desalted using an SCX Stage Tip (Thermo Scientific) before mass spectrometry analysis. For analysis of the SPEARE protein, protein samples (23 to 43 μg), 5 μL color-coded prestained protein marker, high range (Cell Signaling Technology), and 5 μL PageRuler prestained protein marker (Thermo Fisher) were loaded onto 3 to 8% NuPAGE Tris-Acetate Mini Gels (Thermo Fisher Scientific) and electrophoresed at 150 V following the manufacturer’s instructions. The gel was stained using a mass spectrometry-compatible silver staining method as described previously (65). Silver-stained sections were digested by incubation of the gel bands with 40 µL DTT (10 mM) in NH_4_HCO_3_ (50 mM) for 30 min at 37 °C. After removal of the solvent, the bands were incubated with 40 µL iodoacetamide (25 mM) in NH_4_HCO_3_ (50 mM) for 30 min at 37 °C. The bands were washed twice with 50 µL of acetonitrile for 10 min each, 40 µL trypsin (∼100 ng) in NH_4_HCO_3_ (20 mM) was added, and the solution was incubated at 37 °C for 14 h. The bands were washed with 50 µL deionized water plus 1% (vol/vol) formic acid and 100 µL of acetonitrile for 15 min. The extracted peptides were dried and dissolved in 10 µL deionized water with 0.05% (vol/vol) heptafluorobutyric acid and 0.1% (vol/vol) formic acid.

Peptides from in-solution and in-gel digestions were separated by nano-liquid chromatography using an Ultimate nanoRSLC UPLC and autosampler system (Dionex). Samples (2.5 µL) were concentrated and desalted onto a micro-C18 precolumn (300 µm × 5 mm; Dionex) with H_2_O:CH_3_CN (98:2; 0.1% trifluoroacetic acid) at 15 µL min^−1^. After a 4-min wash the solvent flow through from the precolumn was changed (Valco 10 port UPLC valve, Valco) to allow the trapped peptides to elute onto a fritless nanocolumn (75 μm × ∼15 cm) containing C18AQ media (1.9 μm, 120 Å; Dr Maisch). Peptides were eluted using a linear gradient of H_2_O:CH_3_CN (98:2; 0.1% formic acid) to H_2_O:CH_3_CN (64:36; 0.1% formic acid) at 200 nL min^−1^ over 30 min. High voltage of 2,000 V was applied to low-volume Titanium union (Valco), and the tip was positioned ∼0.5 cm from the heated capillary (*T* = 275 °C) of a Orbitrap Fusion Lumos (Thermo Electron) mass spectrometer. Positive ions were generated by electrospray and the Fusion Lumos operated in data-dependent acquisition mode. A survey scan *m/z* 350 to 1,750 was acquired in the orbitrap (resolution = 120,000 at *m/z* 200, with an accumulation target value of 400,000 ions) and lockmass enabled (*m/z* 445.12003). Data-dependent tandem MS analysis was performed using a top-speed approach (cycle time of 2 s). Mass spectrometry stage 2 spectra were fragmented by higher-energy collisional dissociation (normalized collision energy = 30) activation mode, and the ion trap was selected as the mass analyzer. The intensity threshold for fragmentation was set to 25,000. A dynamic exclusion of 20 s was applied with a mass tolerance of 10 ppm. Peak lists were generated using Mascot Daemon/Mascot Distiller (Matrix Science) using default parameters, and submitted to the database search program Mascot (version 2.5.1; Matrix Science). Search parameters were precursor tolerance 4 ppm and product ion tolerances ±0.5 Da, Met (O) carboxyamidomethyl-Cys specified as variable modification; enzyme specificity was trypsin, and 1 missed cleavage was possible. A custom database containing predicted protein coding sequences from *Ca.* Nha. antarcticus, proteins encoded by the host genome, and a database of common contaminant proteins were searched at the same time. A total of 458 proteins with 2 or more unique peptides were identified, of which 279 (∼61%) were annotated with clusters of orthologous genes identifications: 155 information processing and storage (B, J, K, L); 72 metabolism (C, E, F, G, H, I, P, Q); 32 general cellular processes and signaling (D, M, N, O, T, U, V, W, Y, Z). The remaining 179 proteins included 89 hypothetical proteins, of which 62 had no BLAST hits outside of the Nanohaloarchaeota. The mass spectrometry data have been deposited to the ProteomeXchange Consortium via the Proteome Identification (PRIDE) partner repository with Project Name: Cultivation of an Antarctic Nanohaloarchaea and Project accession no. PXD010625.

## Supplementary Material

Supplementary File
