# Supplementary material for: Unexpected host dependency of Antarctic Nanohaloarchaeota
Source: Proc Natl Acad Sci U S A. 2019 Jun 28;116(29):14661–70. doi: 10.1073/pnas.1905179116 (PMC6642349; doi:10.1073/pnas.1905179116)
Supplement: Supplementary File [file pnas.1905179116.sapp.pdf]

Supplementary Information for

Unexpected host-dependency of Antarctic Nanohaloarchaeota

Joshua Hamm, Susanne Erdmann, Emiley A. Eloe-Fadrosh, Allegra Angeloni, Ling Zhong, Christopher Brownlee, Timothy J. Williams, Kirston Barton, Shaun Carswell, Martin A. Smith, Sarah Brazendale, Alyce M. Hancock, Michelle A. Allen, Mark J. Raftery and Ricardo Cavicchioli

Ricardo Cavicchioli
E-mail. r.cavicchioli@unsw.edu.au

This PDF includes:

SI Text
Figures S1-S23
Tables S1-S6
References for SI reference citations

www.pnas.org/cgi/doi/10.1073/pnas.1905179116

Supplementary Information Text

Metabolic and cellular capacity of Nanohaloarchaeota

The Nha-R1 MAG was examined for genes from both archaeal versions of the mevalonate pathway (isoprenoid lipid synthesis), and the non-mevalonate pathway used by bacteria to produce isopentenyl pyrophosphate (IPP). None of the genes from the non-mevalonate pathway were present. *Ca.* Nha. antarcticus does have two genes that are likely involved in isoprenoid lipid synthesis, each with low-quality scores. The first is a short-chain isoprenyl synthase (= dimethylallyltransferase) that constructs the lipid chain by condensation of the isoprenoid building blocks IPP and dimethylallyl pyrophosphate (DMAPP), both of which are generated by the mevalonate pathway. The other is genanylgeranyl reductase, which catalyzes hydrogenation of double bonds in isoprenoid chains (1). Thus, one hypothesis is that *Ca.* Nha. antarcticus employs these enzymes to determine chain length and saturation of its membrane phospholipids, with all other steps (including providing the precursors IPP and DMAPP) carried out by the host. *Hrr. lacusprofundi* ACAM34 encodes the upregulated *hmgA* gene from the pJWID1 recombined on the chromosome thereby enabling it to synthesize lipids in the presence of pravastatin. While *Ca.* Nha. antarcticus lacks *hmgA* and would therefore not be inhibited by pravastatin, its ability to form membranes requires that it acquires lipids from another source, with *Hrr. lacusprofundi* being metabolically capable of being the provider.

All glycolysis (Embden-Meyerhof-Parnas pathway) proteins were detected in the proteome, except phosphoglycerate mutase (Table S2) (encoded in the genome, but not detected in the proteome). Detected glycolytic proteins include a bifunctional ADP-specific glucokinase/phosphofructokinase; it has been proposed that ADP-specific sugar kinases are correlated with a low-energy metabolism where availability of ATP is limited (2). Fructose 1,6-bisphosphatase I and phosphoenolpyruvate synthase were also detected, indicating gluconeogenesis was also functioning. The end-product of gluconeogenesis is likely glucose-6-phosphate (glucose-6-phosphatase is not encoded). One possible fate of glucose-6-phosphate is that it is directed to glycogen synthesis; putative glycogen synthesis enzymes were detected (phosphoglucomutase, UDP-glucose pyrophosphorylase, glycogen synthase, debranching enzyme). Also detected were a putative S-layer protein and several proteins inferred to be involved in glycosylation and sulfation of the S-layer. *Ca.* Nha. antarcticus lacks an identifiable tricarboxylic acid (TCA) cycle and electron transport chain complexes, as is common in other DPANN archaea (3), and there is no identifiable pentose phosphate

pathway. Pyruvate dehydrogenase complex proteins and acetate-CoA ligase were detected, for conversion of pyruvate to acetyl-CoA and then to acetate. Glycolysis and acetyl-CoA cleavage generate ATP through substrate-level phosphorylation, consistent with a fermentative metabolism. A D-lactate dehydrogenase was also detected, which is possibly a means for regenerating NAD^+. Electron transfer proteins were detected, including a possible NADH oxidase that would also serve to replenish NAD^+, as well as protect against oxidative stress. Several DNA repair enzymes were detected, as well as superoxide dismutase, consistent with a cellular response to DNA damage and oxidative stress. In the absence of catalase (not encoded), *Ca.* Nha. antarcticus could use alkyl hydroperoxide reductase to reduce H_2O_2.

Components of an A-type ATPase were detected, which may function as an ATP synthase. *Ca.* Nha. antarcticus encodes a rhodopsin that is most closely related to haloarchaeal sensory rhodopsins, but does not encode a cognate Htr transducer. In the absence of this transducer, sensory rhodopsins serve as outward-directed H^+ pumps (4); thus, one hypothesis is that the *Ca.* Nha. antarcticus rhodopsin serves as a light-induced H^+ pump that establishes a chemiosmotic gradient that is coupled to ATP synthesis. Alternatively, the A-type ATPase might extrude H^+ to establish a proton motive force to drive antiporters. The genome encodes a seven-subunit Mrp-type Na^+: H^+ antiporter. Additionally, a cation:H^+ exchanger and a Na^+ exporter ATPase were detected in the proteome. These diverse transporters could be involved in Na^+ efflux, as adaptations to a hypersaline environment.

The reduced biosynthetic capacity of *Ca.* Nha. antarcticus would necessitate exogenous uptake, particularly from the host. The extracytoplasmic solute-binding component of the single ABC transporter system for uptake of free amino acids was detected, indicating scavenging of exogenous amino acids. Secreted and cytoplasmic proteases or peptidases were detected, along with enzymes that catalyze the interconversion of certain amino acids, e.g., serine hydroxymethyltransferase, asparagine synthetase (glutamine-hydrolyzing), and broad-specificity class I aminotransferase (aspartate aminotransferase [AspAT]). Enzymes involved in the synthesis of tyrosine and phenylalanine from chorismate were detected, but *Ca.* Nha. antarcticus lacks an identifiable shikimate pathway, so presumably chorismate is acquired exogenously. Glutamate dehydrogenase (GDH) was detected and shows the closest identity to catabolic GDH, which produces 2-oxoglutarate and ammonium. If aspartate is available, 2-oxoglutarate could be converted to oxaloacetate by AspAT, which could then be converted to pyruvate using malate dehydrogenase (oxaloacetate decarboxylating) (detected in the proteome). Thus, degradation of certain amino acids to pyruvate would allow these

compounds to be used for energy conservation and gluconeogenesis, as well as for synthesizing proteins. However, unless GDH can also act as an assimilatory enzyme in response to high ammonia levels, *Ca.* Nha. antarcticus cannot assimilate ammonium as it lacks genes for glutamine synthetase and glutamate synthase. A two-step citrate cleavage pathway is evident in the genome, involving citryl-CoA synthetase and citryl-CoA lyase, with acetyl-CoA and oxaloacetate as products, which could be catabolized further to generate ATP. The citrate is presumably obtained exogenously, and might be imported using a TctA family transporter. Thus, although *Ca.* Nha. antarcticus has no TCA cycle, it appears to be capable of catabolizing TCA cycle intermediates as energy sources.

Proteins required for replication, transcription (including transcriptional regulation), and translation were especially prominent in the proteome; the latter included all 18 amino acyl-tRNA synthetases, and glutamyl- and aspartyl-tRNA amidotransferases, which indicates that cells are poised to incorporate amino acids into proteins. The proteome was also notable for many proteins with nuclease domains, as well as enzymes involved in the regeneration of nucleotides, and pyrophosphatase (non-proton-pumping). Proteasome and exosome complex proteins were detected, indicating turnover of proteins and RNA.

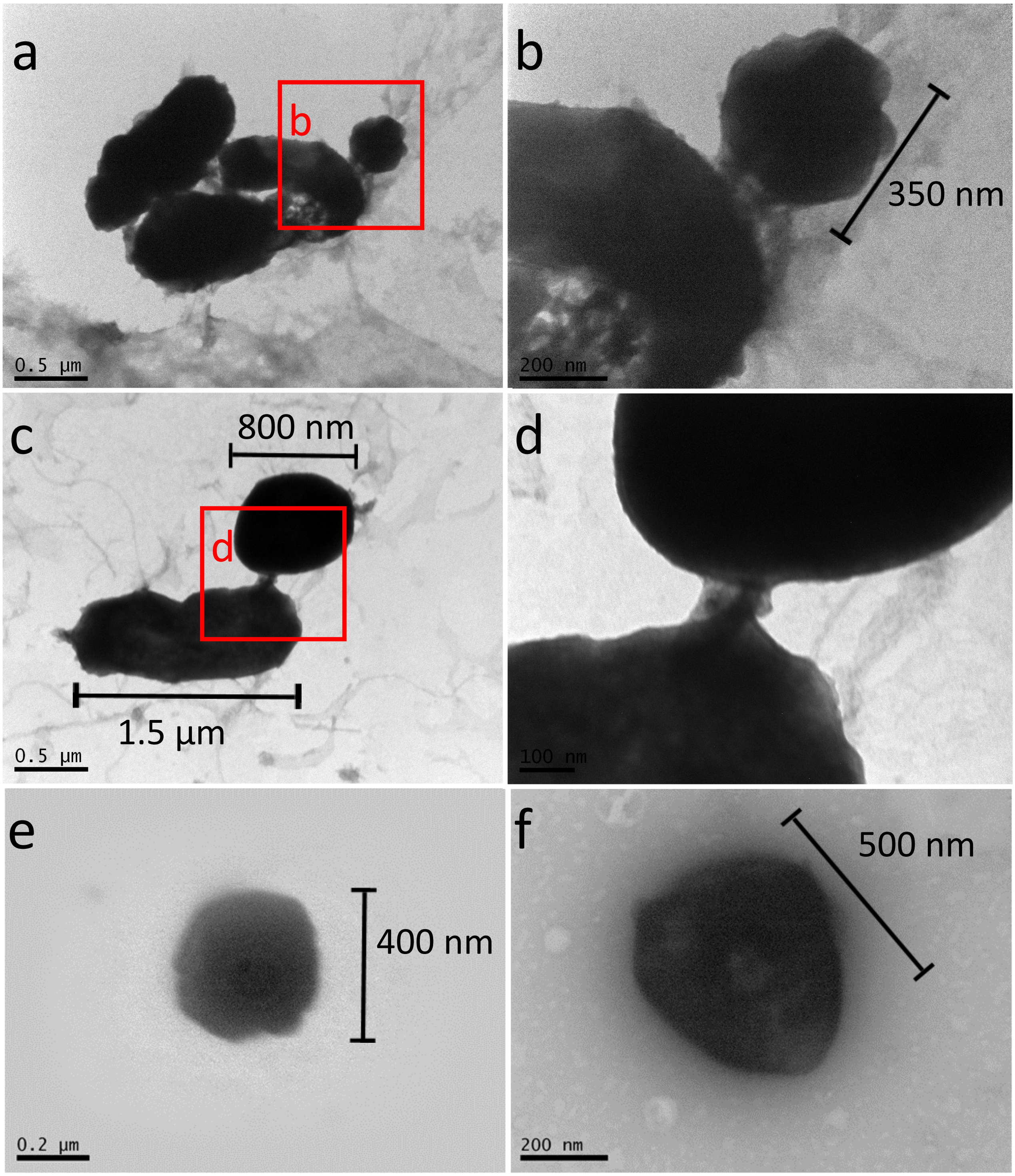

Fig. S1. TEM of Nha-R1 enrichment and Nha-R1 filtrate used for growth experiments. Small cells in close contact with larger cells, consistent with Nha-C associating with *Hrr. lacusprofundi* (**A-D**). Small (putative Nha-C) coccoid cells ranged in diameter from ~0.15 – ~1.0 µm. Larger cells (putative *Hrr. lacusprofundi*) ranged in size from ~1 - ~3 µm and were typically pleomorphic rods. The appearance of possible extracellular material may indicate an involvement in interactions between Nha-C and *Hrr. lacusprofundi* (**A-D**). TEM of the 0.22 µm filtrate of the Rauer 1 Lake enrichment that was used as inoculum for cultivation experiments to attempt to grow *Ca.* Nha. antarcticus independently (**G,H**); the images revealed the presence of predominantly small cocci.

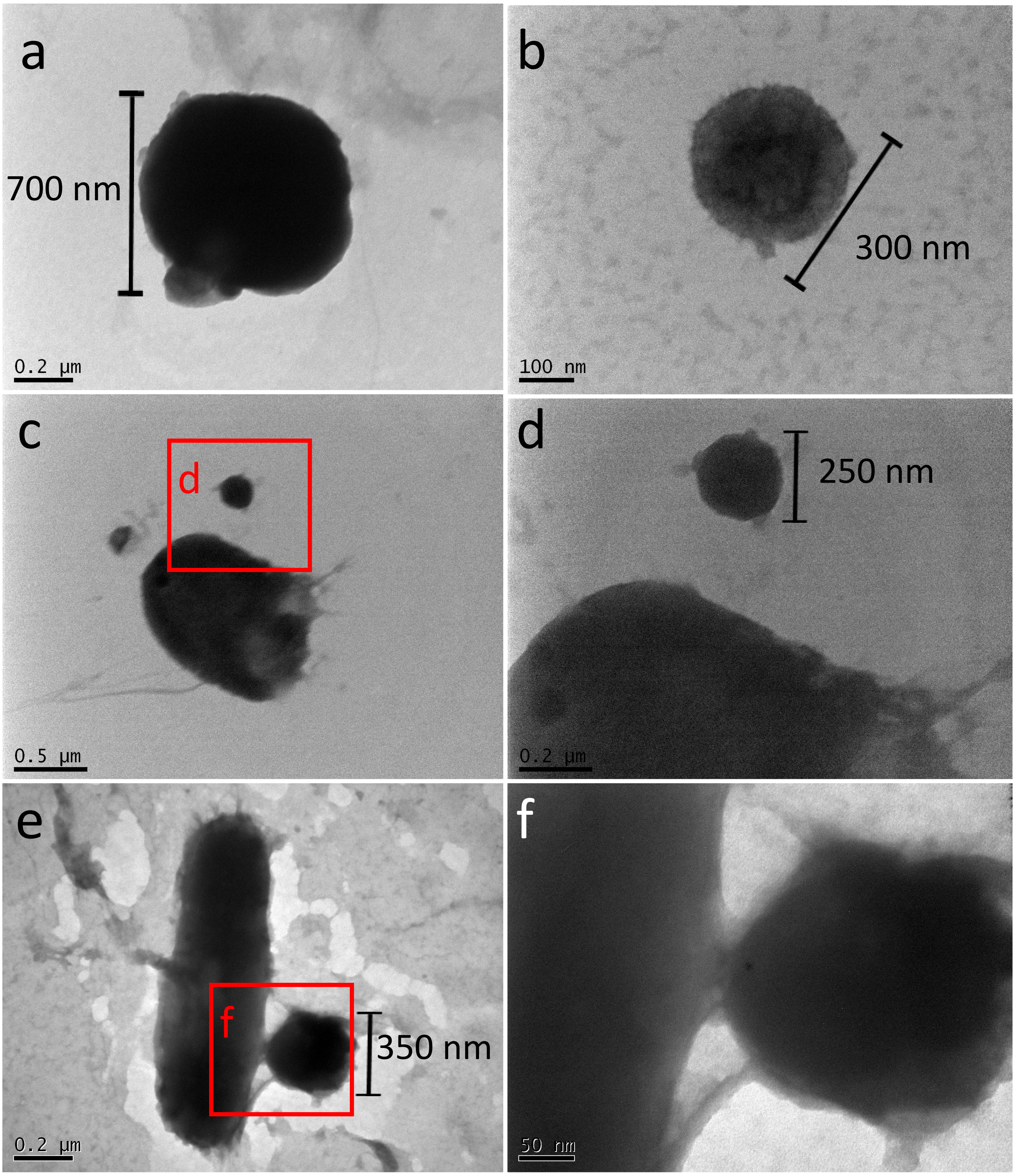

Fig. S2. TEM of Nha-C enrichment. Small individual cells consistent with being Nha-C (**A-D**). Small cells in close contact with larger cells, consistent with Nha-C associating with *Hrr. lacusprofundi* (**E,F**). The appearance of possible extracellular material may indicate an involvement in interactions between Nha-C and *Hrr. lacusprofundi* (**E,F**).

a

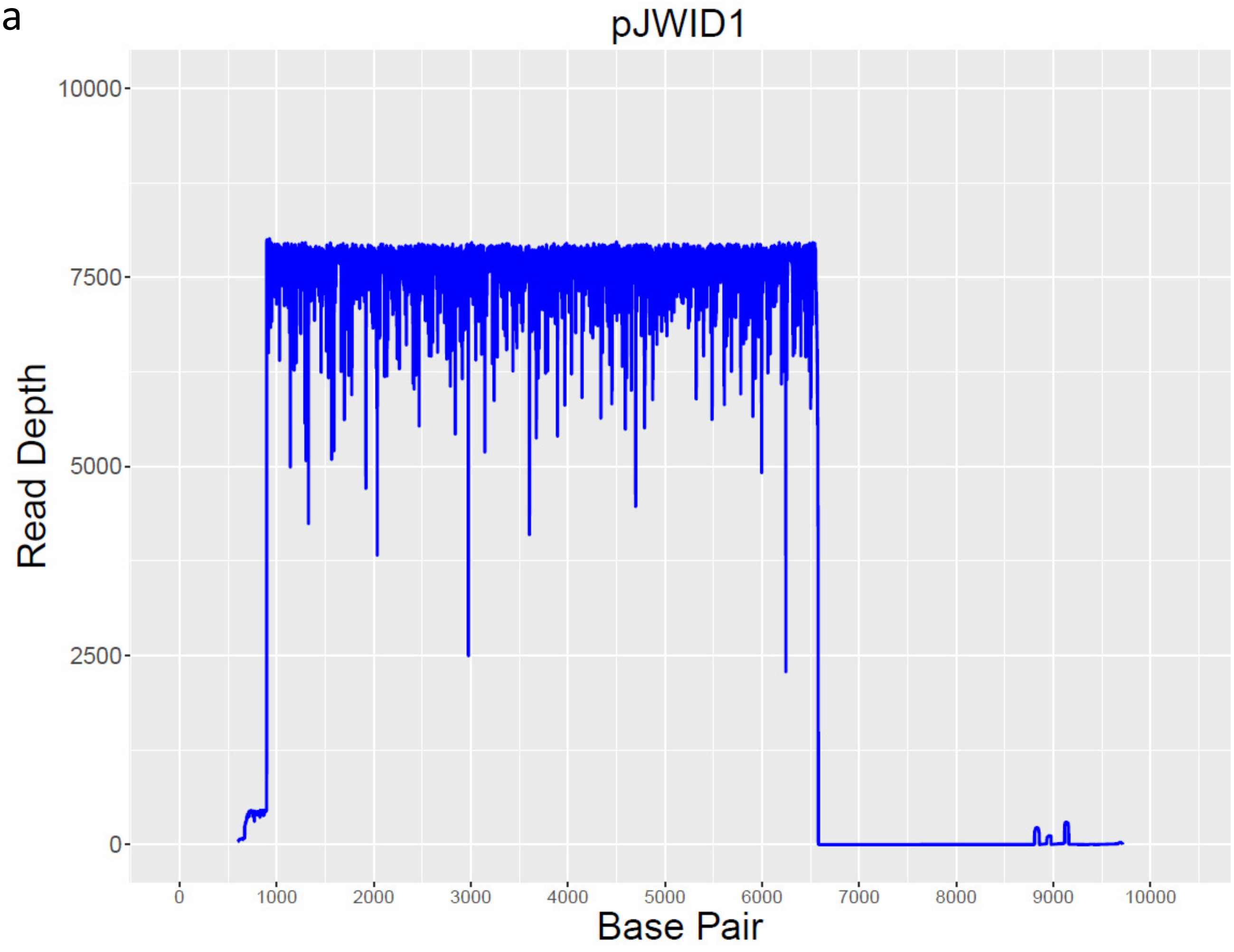

b

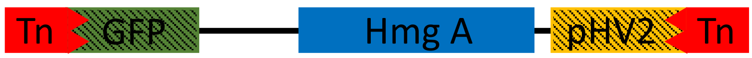

Fig. S3. Relocation of the *hmgA* gene from pJWID1 to the chromosome of *Hrr. lacusprofundi* ACAM34. Growth at high concentrations of pravastatin (up to 10 μg ml^{-1}) led to selection of a strain (referred to as *Hrr. lacusprofundi* ACAM34-hmgA) where the upregulated hmgA gene from pJWID1 (5) relocated to the chromosome of *Hrr. lacusprofundi* ACAM34 flanked by *Hrr. lacusprofundi* transposases (IMG gene ID: 643710163). Insertion of the transposase interrupted both the GFP gene and *Haloferax volcanii* origin of replication, rendering both non-functional. Insertion of the plasmid sequence also led to duplication at each insertion point, with copy number ranging from 1-5 per insertion event and plasmid sequences inserting onto all three of the *Hrr. lacusprofundi* replicons. Average read depth across the inserted pJWID1 sequence was approximately 8000, while coverage across the *Hrr. lacusprofundi* genes flanking the insertion point was approximately 500.

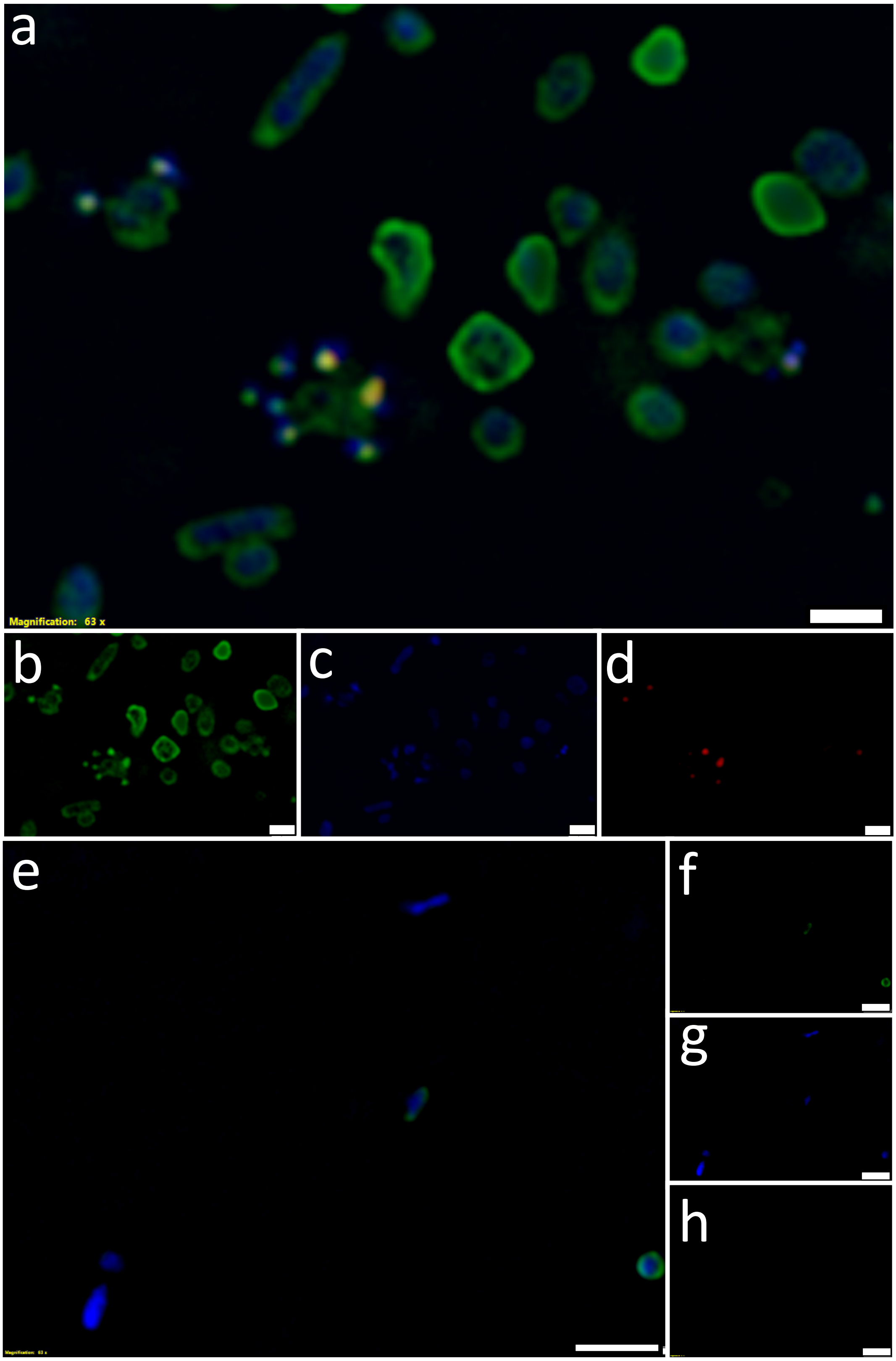

Fig. S4. FISH of Nha-C enrichment with *Hrr. lacusprofundi* ACAM34-hmgA. Fluorescence micrographs show Nha-C cells in contact with *Hrr. lacusprofundi* cells (**A-D**). Large cells fluorescing blue (DAPI) but not fluorescing for Nha-C or *Hrr. lacusprofundi* specific probes represent other species in the enrichment (e.g. *Natrinema* and bacteria) (**E-H**). Equivalently sized cells hybridizing to the *Hrr. lacusprofundi* specific probes are present in the same field of view (compare **E,F** with **G**). Nha-C cells labelled with a Cy5 (red fluorescence) conjugated probe; *Hrr. lacusprofundi* cells labelled with a Cy3 (yellow fluorescence, recoloured to green to improve contrast) probe; all nucleic-acid containing cells stained with DAPI (blue fluorescence). Composite image of all three filters (**A, E**). Individual filters for Cy3 (**B, F**), DAPI (**C, G**), Cy5 (**D, H**). Scale bars represent 2 µm.

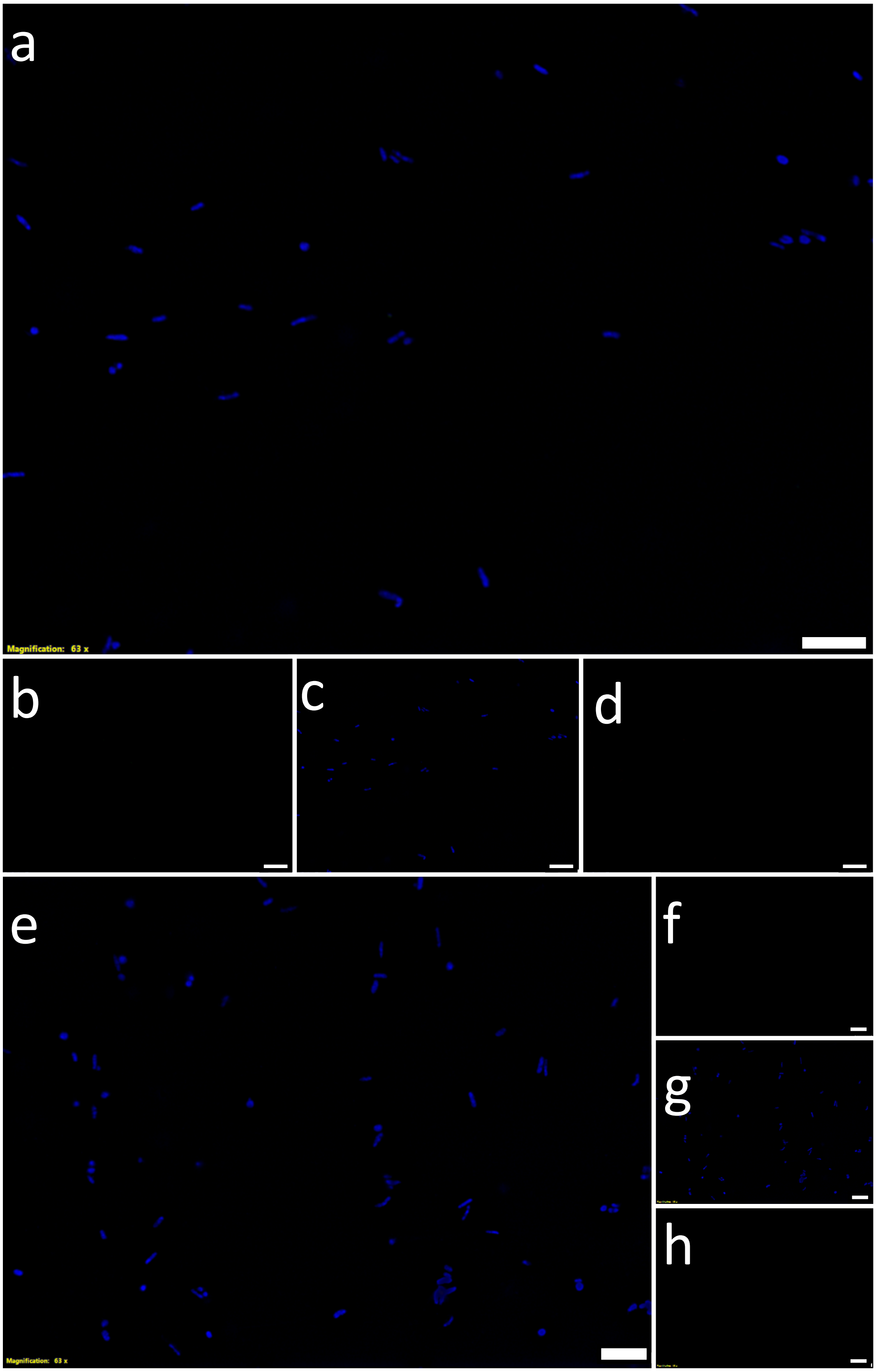

Fig. S5. FISH of *Natrinema* sp. Fluorescence micrographs show lack of fluorescence to Nha-C or *Hrr. lacusprofundi* specific probes while cells fluorescing blue (DAPI). Cy5 Nha-C specific probe (red fluorescence); Cy3 *Hrr. lacusprofundi* specific probe (yellow fluorescence, recoloured to green to improve contrast); all nucleic-acid containing cells stained with DAPI (blue fluorescence). Composite image of all three filters (**A, E**). Individual filters for Cy3 (**B, F**), DAPI (**C, G**), Cy5 (**D, H**). Scale bars represent 2 µm.

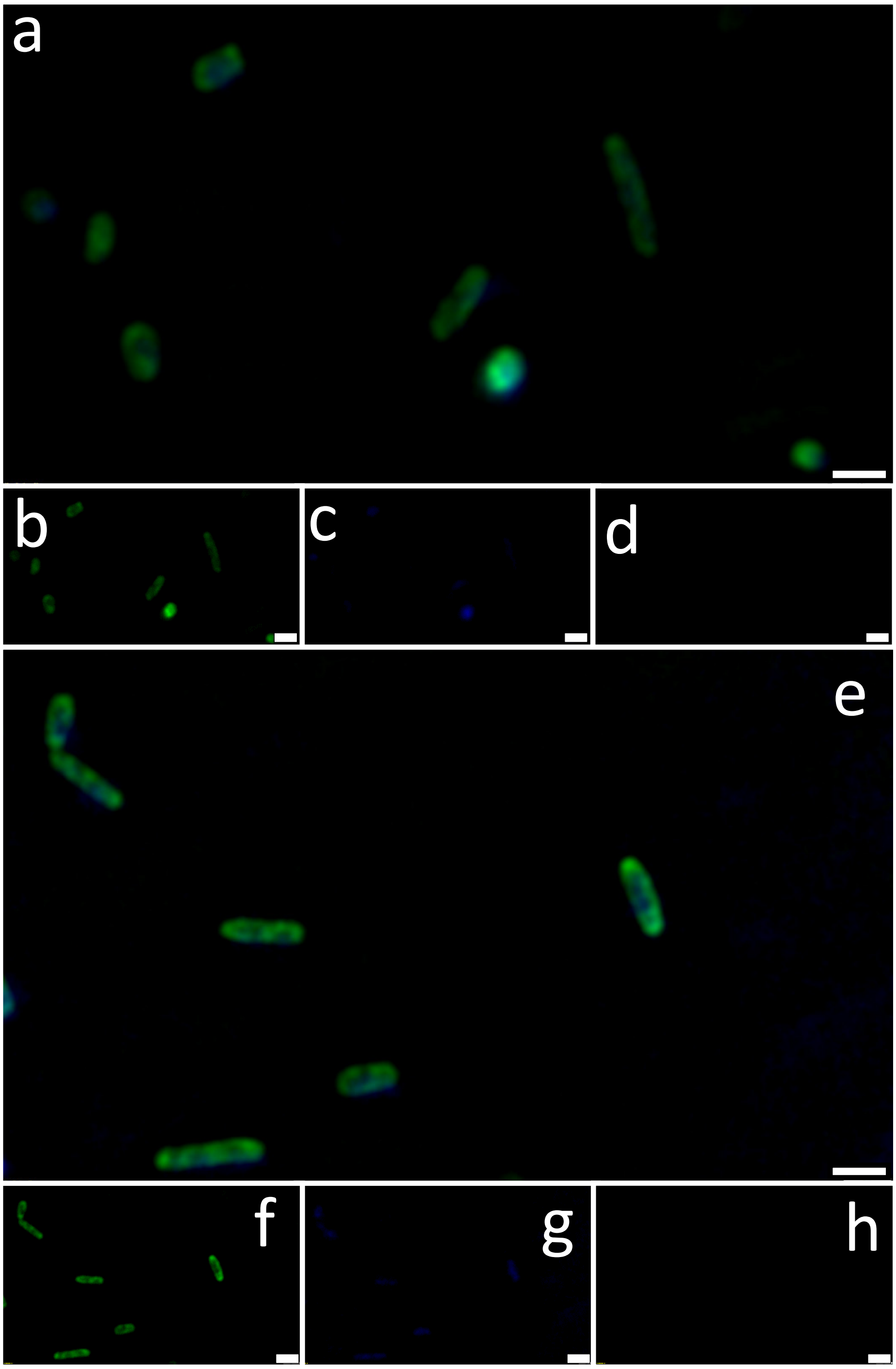

Fig. S6. FISH of *Hrr. lacusprofundi* ACAM34. Fluorescence micrographs show fluorescence to the *Hrr. lacusprofundi* specific probe but not the Nha-C specific probe, and fluorescence to DAPI. Cy5 Nha-C specific probe (red fluorescence); Cy3 *Hrr. lacusprofundi* specific probe (yellow fluorescence, recoloured to green to improve contrast); all nucleic-acid containing cells stained with DAPI (blue fluorescence). Composite image of all three filters (**A, E**). Individual filters for Cy3 (**B, F**), DAPI (**C, G**), Cy5 (**D, H**). Scale bars represent 2 µm.

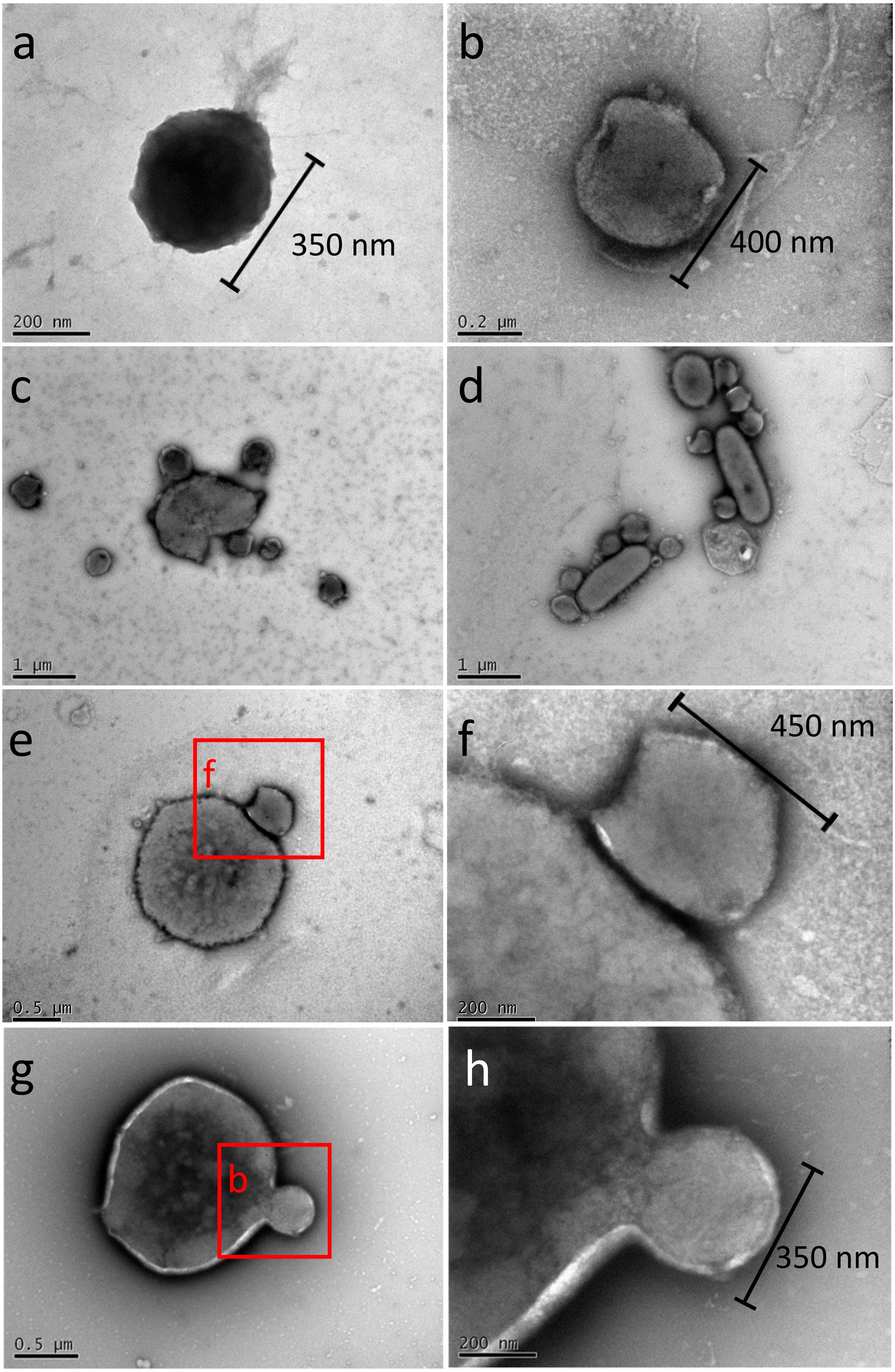
a
350 nm
200 nm
b
400 nm
0.2 µm
c
1 µm
d
1 µm
e
f
0.5 µm
f
450 nm
200 nm
g
b
0.5 µm
h
350 nm
200 nm

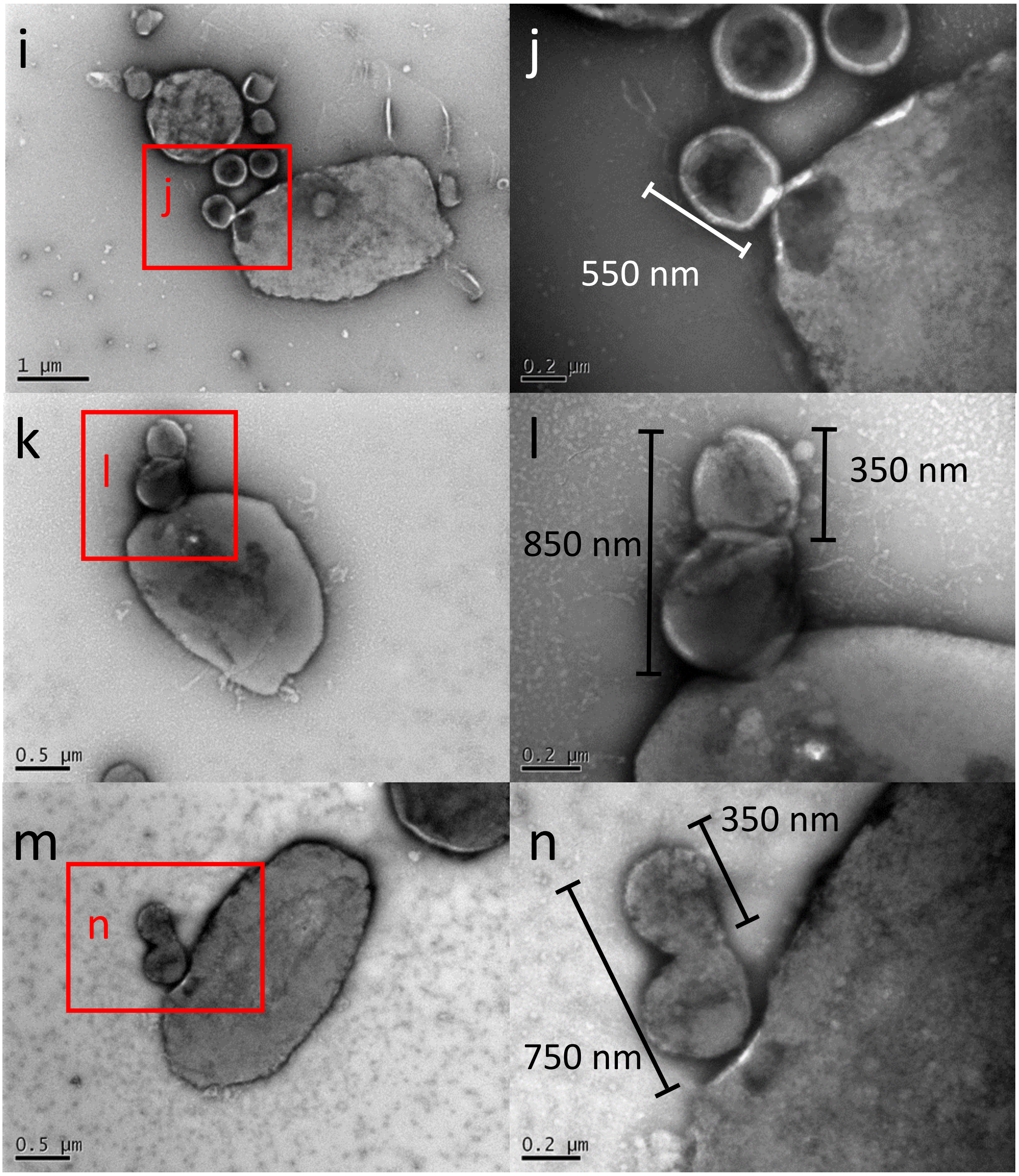

Fig. S7. TEM of Nha-C enrichment with *Hrr. lacusprofundi* ACAM34-hmgA. Small individual cells consistent with being Nha-C (**A,B**). Small cells that appear to have intact membranes with minimal visible boundary layers separating them from larger cells, consistent with Nha-C associating with *Hrr. lacusprofundi* (**C-F**). Small cell that appears to be fused to a large cell, consistent with the membranes of Nha-C and *Hrr. lacusprofundi* being connected, possibly occurring during attachment and/or prior to separation (**G,H**). Small cell in intimate contact or near a large cell; the cell in contact is consistent with Nha-C intimately associating with *Hrr. lacusprofundi* (**I,J**). Small divided cells with minimal visible boundary layers separating them from a larger cell, consistent with Nha-C cells having divided while in contact with *Hrr. lacusprofundi* (**K-N**).

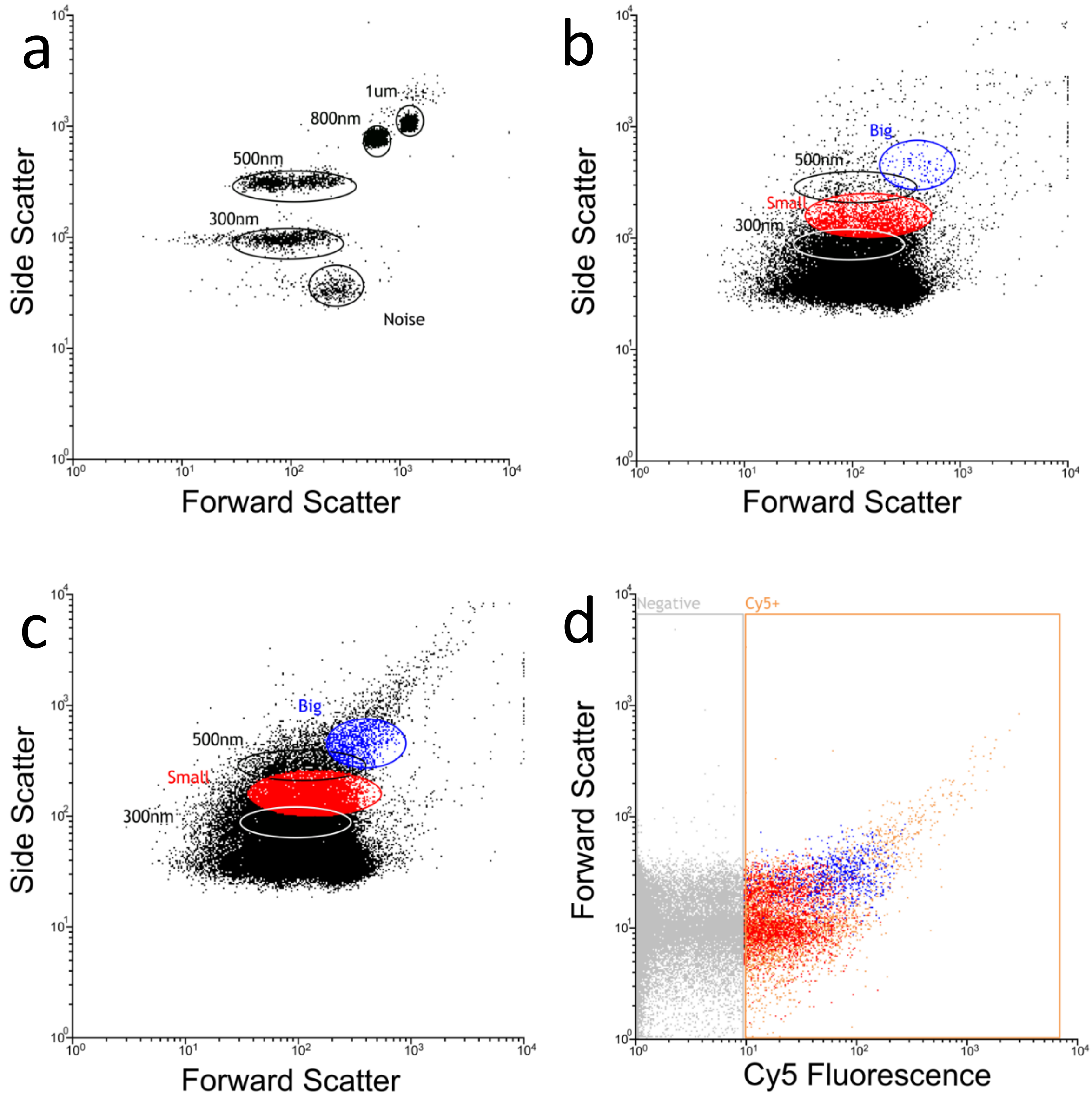

Fig. S8. Flow cytometry analysis of Nha-C cells. A Nha-C enrichment with high relative abundance of Nha-C (47 %) was used to obtain purified samples of Nha-C. Forward and side scatter plot of standard size beads used to calibrate the cytometer (**A**). Forward and side scatter plot of live cells subjected to sorting with gates used for sorting marked (Small, ~400nm, red; Big, ~600nm, blue) (**B**). Forward and side scatter plot of cells subjected to FISH using Cy5 Nha-C specific and Cy3 *Hrr. lacusprofundi* specific probes, with gates used for sorting marked (**C**). Cy5 fluorescence and forward scatter plot of cells subjected to FISH, showing cells within the size gates used for sorting (Cy5+, orange box) only fluoresced for the Nha-C specific probe, compared to those outside the gate (Negative, grey box) (**D**). 'Small' and 'Big' (**C,D**) coloured as for (**B**).

Sort Report

System

Sort Start:	9/27/2018 2:23:36 PM	Server:	Utopex
Application:	BD FACS™ Sortware	Build:	1.2.0.107
Version:	1.2.0.117	Cytometer Model:	BD Influx System
ValComp:	7.5.1.3.16	Cytometer Serial #:	X646500I2001

Details

Data Source:	Cytometer	Sort Mode:	1.5 Drop Pure
Nozzle Diameter (µm):	0.00	Drop Envelope:	1.5 Drops
Sheath Pressure (PSI):	0.00	Sort Objective:	Purify
Sort Device:	2 Tube Holder - 2 Way Sort	Phase Mask:	16/16
Piezo Amplitude:	0.18	Extra Coincidence Bits:	4
Drop Delay:	45.8111	Drop Frequency (kHz):	38.40

Sort Details

Name	Population	Event Limit	Event Count	Sort Count	Sort Rate	Aborts	Abort Rate	Efficiency	Time (sec)
Left (Totals	Big	Unlimited	21,690,302	806,881	202	138,499	33	85.1%	3,981
=> Run 1	Big	Unlimited	8,775,280	100,532	55	18,154	9	84.7%	1,827
=> Run 2	Big	Unlimited	12,915,022	706,349	327	120,345	55	85.4%	2,154
Right (Tota	Small	Unlimited	21,693,768	1,568,150	393	241,421	60	86.5%	3,982
=> Run 1	Small	Unlimited	8,776,326	290,879	159	46,363	25	86.3%	1,827
=> Run 2	Small	Unlimited	12,917,442	1,277,271	592	195,058	90	86.8%	2,155

Fig. S9. FACS report for Nha-C cells used for growth studies. Sort report for the final FACS run. Consecutive rounds of sorting collected 1,568,150 (~400 nm dia) and 806,881 (~600 nm dia) live cells from a total of ~21,700,000 sorted cells.

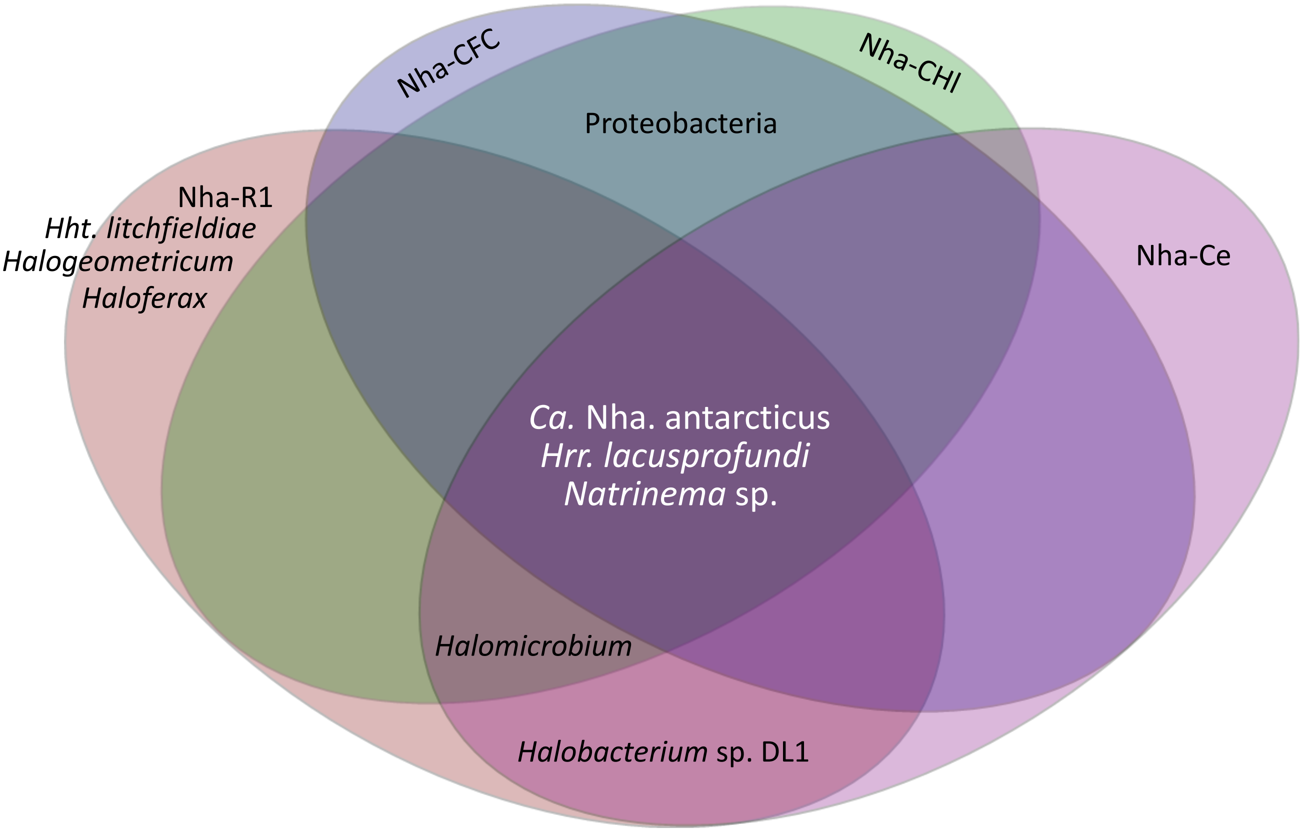

Fig. S10. Taxa common to all *Ca.* Nha. antarcticus metagenomes. Venn-diagram showing that the only taxa common to all enrichment metagenomes were *Ca.* Nha. antarcticus, *Hrr. lacusprofundi* and *Natrinema* sp.

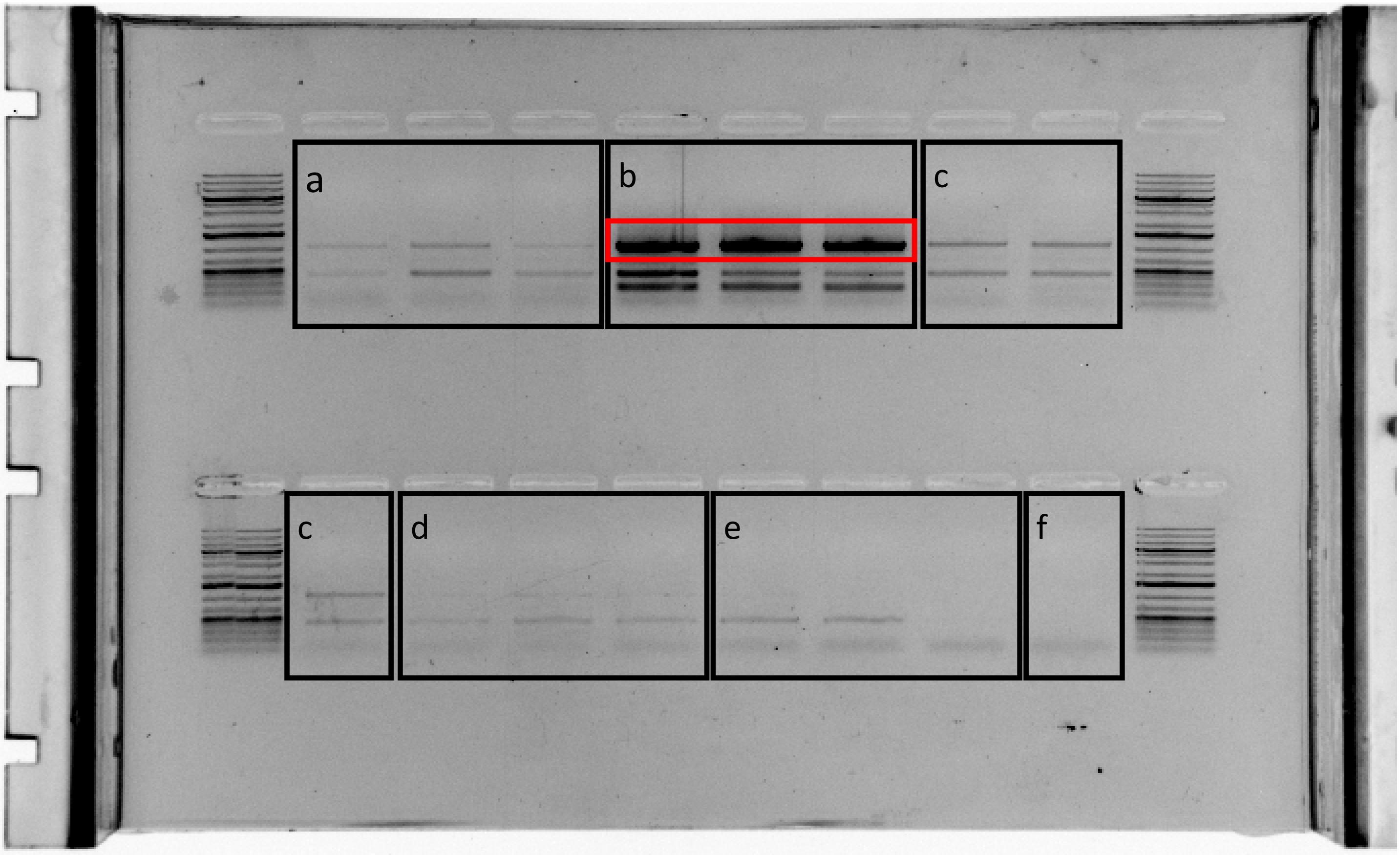

Fig. S11. PCR-based assessment of the presence of Nha-C grown with *Hrr. lacusprofundi*. Agarose gel (1%) stained using Sybr Safe DNA stain (ThermoFisher Scientific, Massachusetts, USA) and imaged on a GelDoc XR+ (Bio-Rad Laboratories, California, USA) showing banding patterns from PCR performed using Nha-C 16S rRNA primer sets. PCR was performed after Nha-C was incubated with *Hrr. lacusprofundi* and grown for ~ 5 weeks (**A**). Fresh media was then added and PCR performed at early exponential growth (8 d) (**B**), late exponential growth (21 d) (**C**), early stationary phase (30 d) (**D**), and late stationary phase (98 d) (**E**). Lanes represent the three replicates (one originating from the 'Small' and two from the 'Big' size fractions; see Fig. S8). Negative control (water) (**F**). Bands highlighted in red were excised from the gel and sequenced. DNA ladders in the outside lanes of the gel are GeneRuler 1kb+ (ThermoFisher Scientific, Massachusetts, USA).

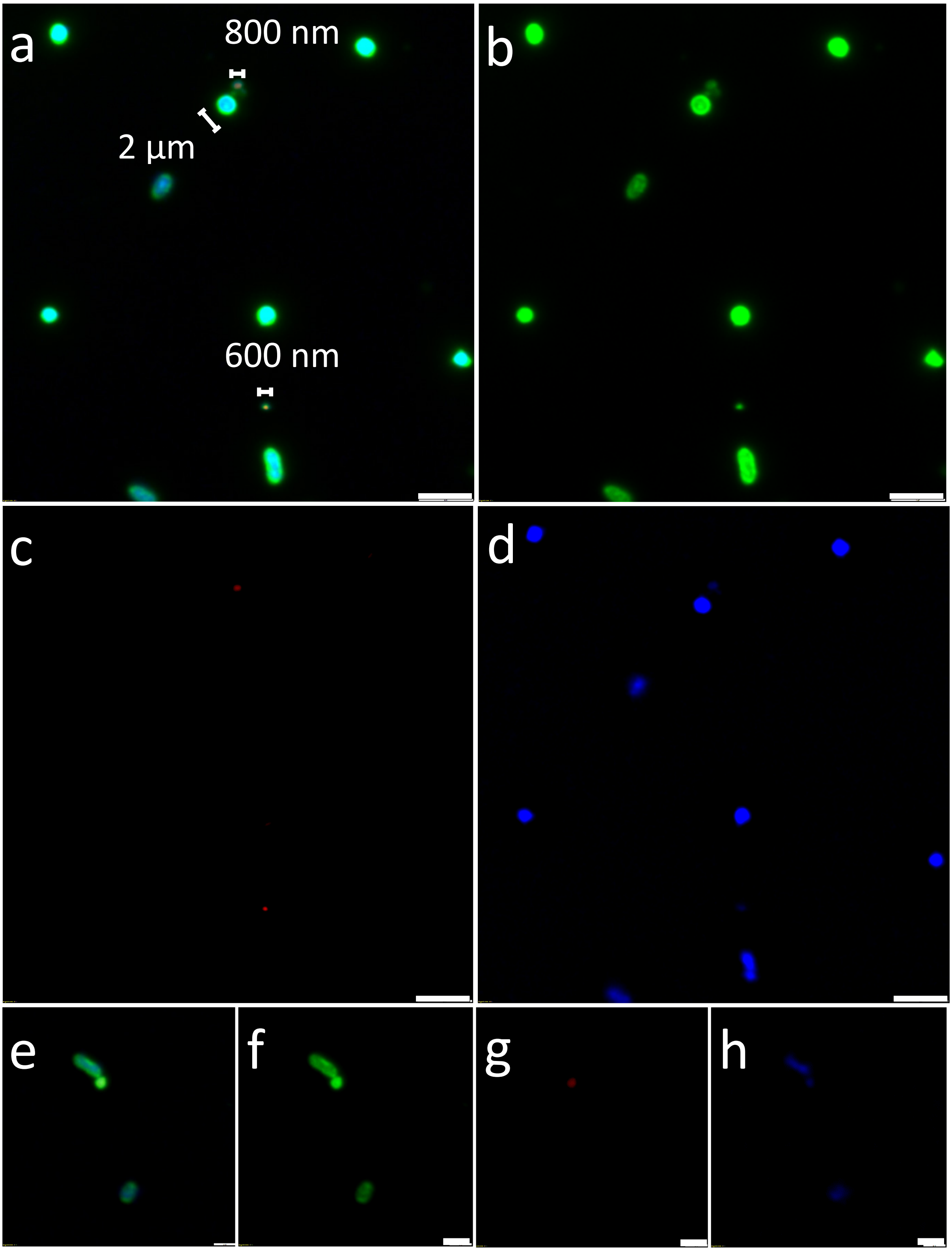

Fig. S12. FISH of Nha-C FACS cells with *Hrr. lacusprofundi* ACAM34. Fluorescence micrographs show Nha-C cells in contact with *Hrr. lacusprofundi* cells (**A-H**) and as an individual isolated cell (**A-D**). Nha-C cells labelled with a Cy5 (red fluorescence) conjugated probe; *Hrr. lacusprofundi* cells labelled with a Cy3 (yellow fluorescence, recoloured to green to improve contrast) probe; all nucleic-acid containing cells stained with DAPI (blue fluorescence). Composite image of all three filters (**A, E**). Individual filters for Cy3 (**B, F**), DAPI (**C, G**), Cy5 (**D, H**). Scale bars represent 2 µm.

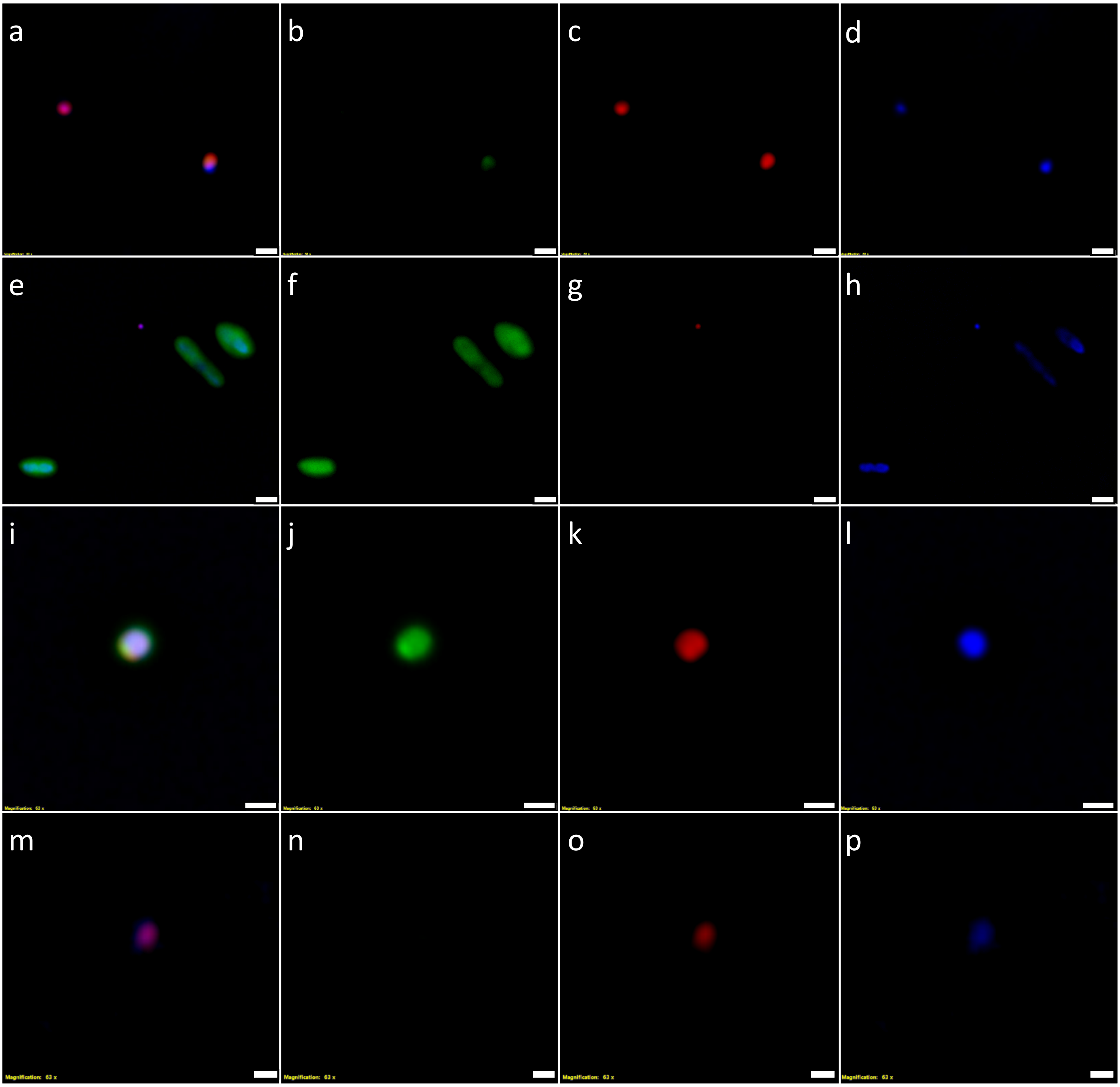

Fig. S13. FISH showing Nha-C cells fluorescing only with the Nha-C probe or co-fluorescing with the *Hrr. lacusprofundi* probe. Nha-C FACS cells with *Hrr. lacusprofundi* ACAM34. Fluorescence micrograph showing a Nha-C cell fluorescing only with the Nha-C probe and in the same field, one cell co-fluorescing with both the Nha-C and *Hrr. lacusprofundi* probes (**A-D**). Nha-C cell fluorescing only with the Nha-C probe (**E-H, M-P**). Nha-C cell co-fluorescing with both the Nha-C and *Hrr. lacusprofundi* probes (**I-L**). Nha-C cells labelled with a Cy5 (red fluorescence) conjugated probe; *Hrr. lacusprofundi* cells labelled with a Cy3 (yellow fluorescence, recoloured to green to improve contrast) probe; all nucleic-acid containing cells stained with DAPI (blue fluorescence). Composite image of all three filters (**A, E**). Individual filters for Cy3 (**B, F**), DAPI (**C, G**), Cy5 (**D, H**). Scale bars represent 2 µm.

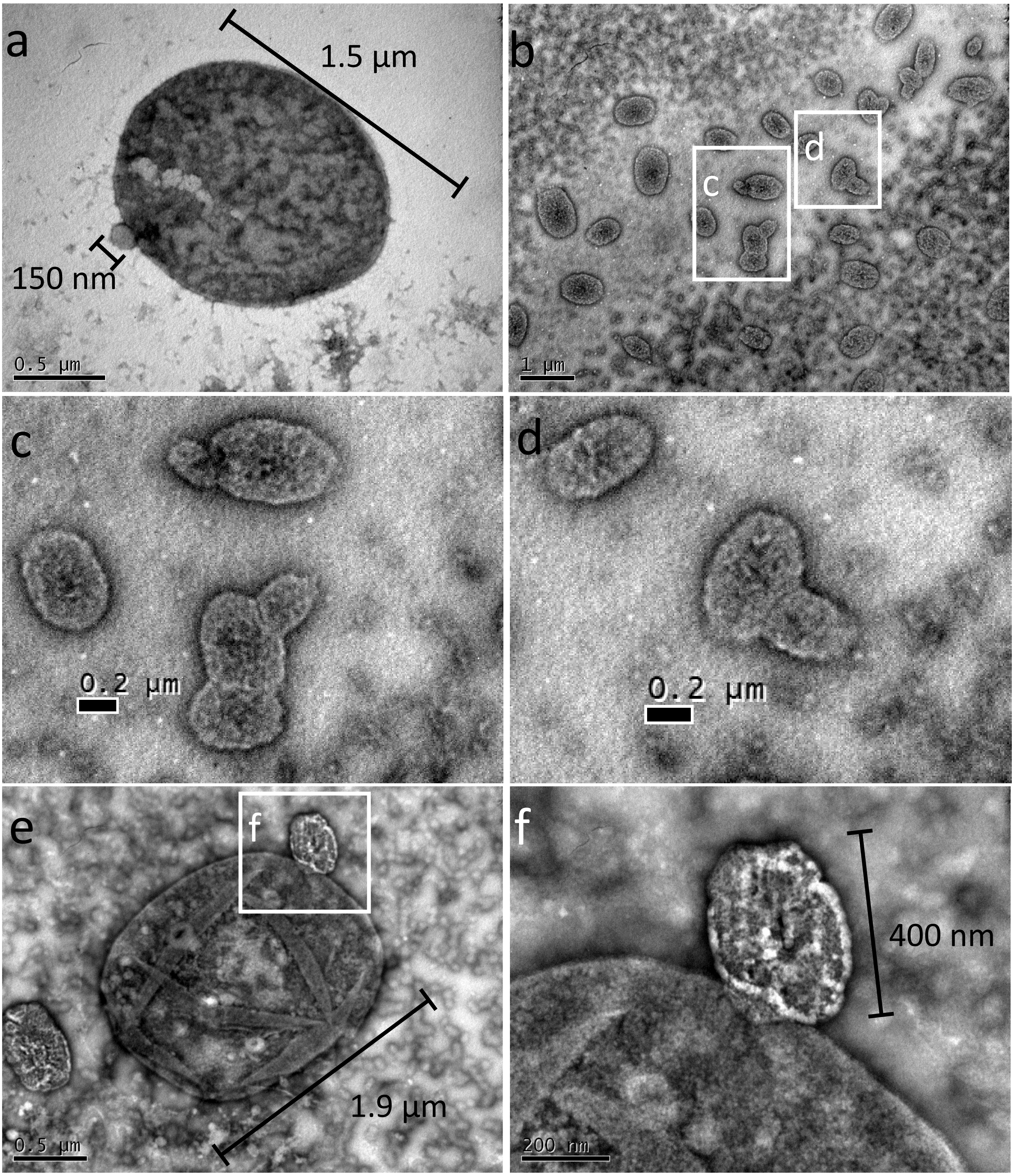

Fig. S14. TEM of Nha-C FACS cells with *Hrr. lacusprofundi* ACAM34. Very small cell in contact with large cell, consistent with Nha-C associating with *Hrr. lacusprofundi* (**A**). Multiple small cells not associated with large cells, consistent with them being Nha-C cells (**B-D**). A number of the cells appear to have divided. Division of Nha-C may have occurred while attached to *Hrr. lacusprofundi*, followed by separation from the host. It is also possible the Nha-C underwent limited cell division after separation from *Hrr. lacusprofundi*. The staining towards the top left and bottom right may be extracellular material (**B**). A small cell associated with a large cell that appears to be lysed (as indicated by bands extending across the cell), consistent with Nha-C in contact with a lysed *Hrr. lacusprofundi* cell (**E-F**).

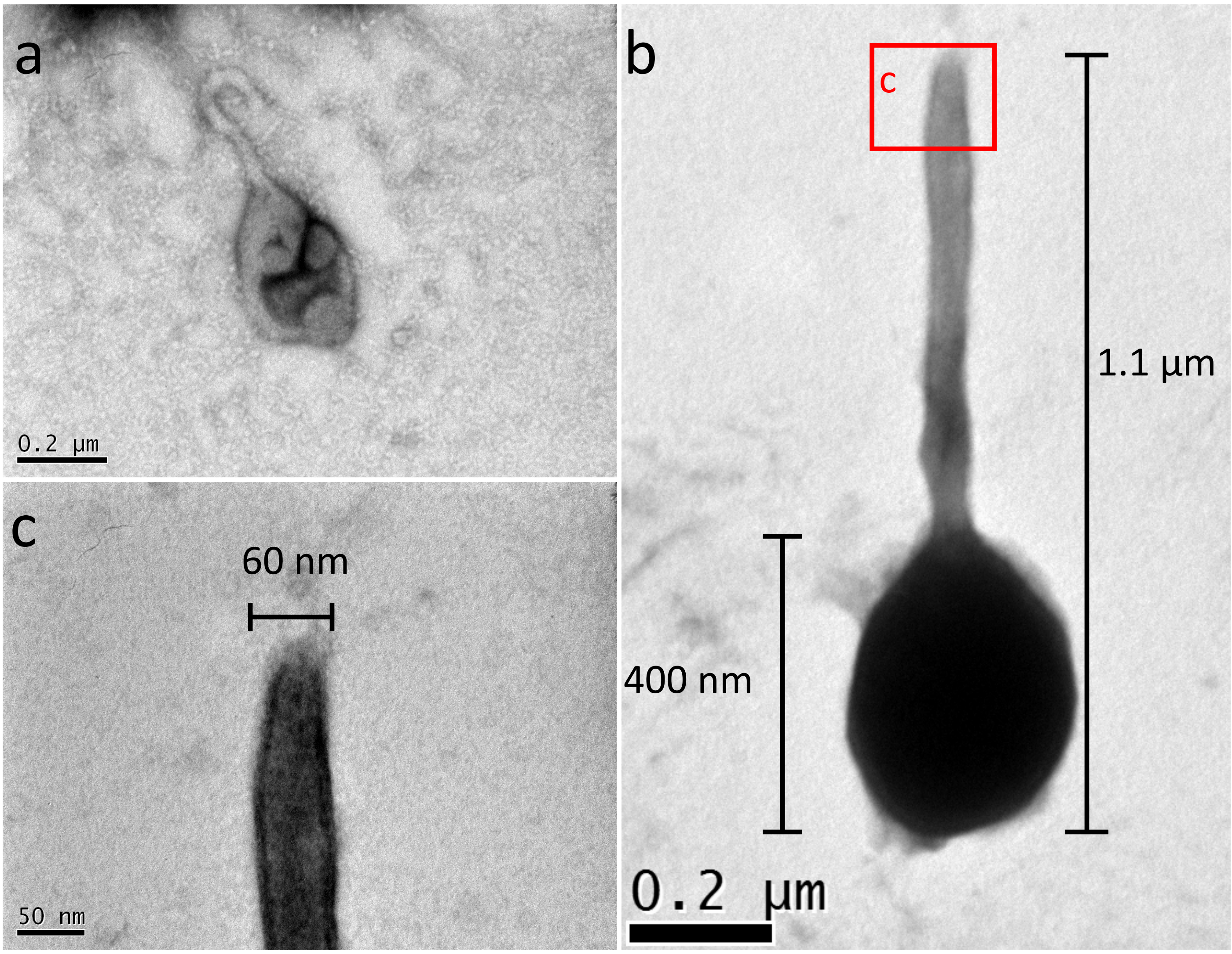

Fig. S15. TEM of Nha-C FACS cells with *Hrr. lacusprofundi* ACAM34 showing extracellular structures. Small cells with apparent appendages, consistent with Nha-C cells with remnants of cytoplasmic bridge or other membrane associated structures (**A-C**). The enhanced image appears to show intact exterior walls (possibly membranes) with a perfuse tip (**C**). The tip may represent the point at which contact with the host was severed.

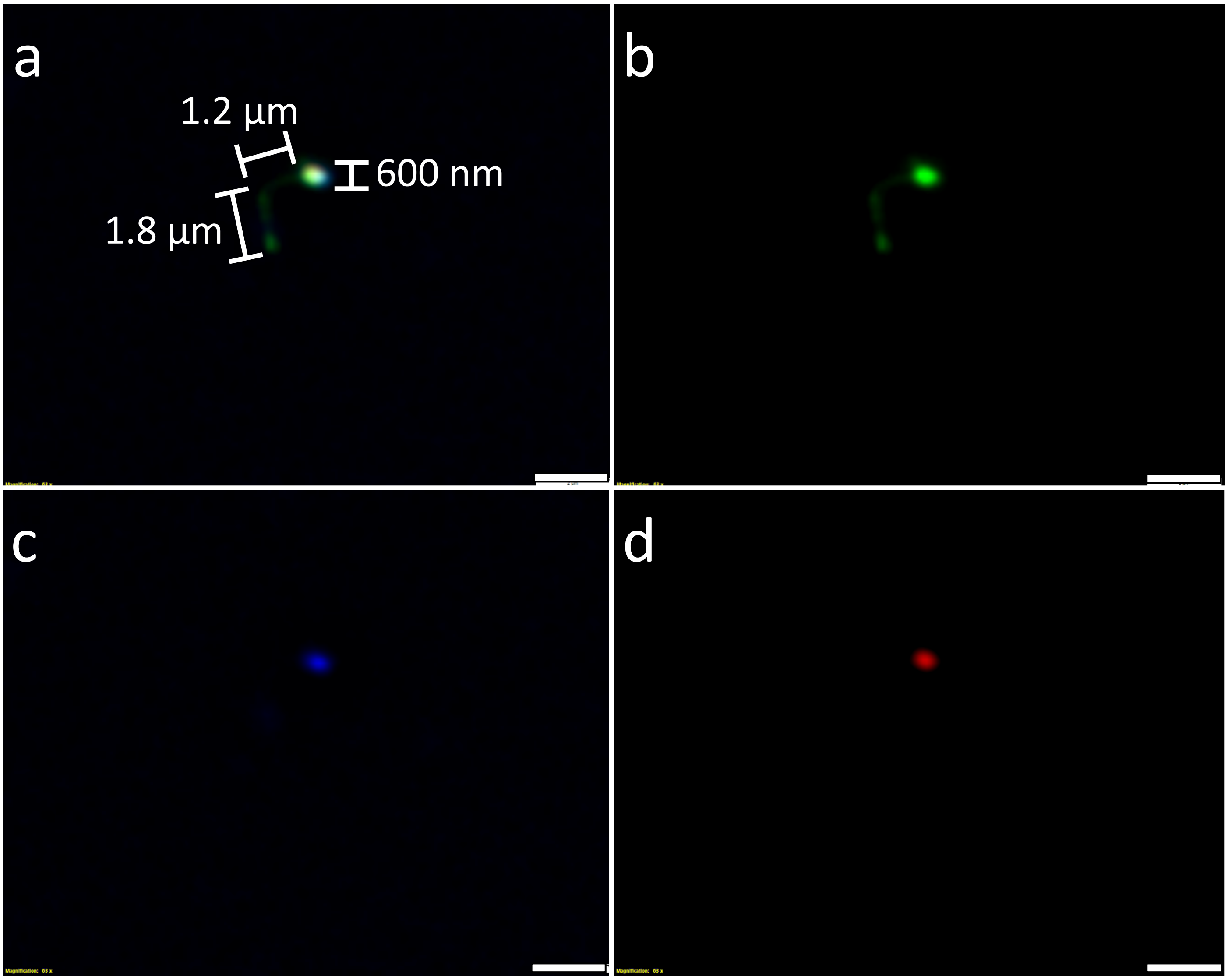

Fig. S16. FISH of Nha-C FACS cells with *Hrr. lacusprofundi* ACAM34 showing possible cytoplasmic bridges. Fluorescence micrograph of a Nha-C cell appearing to show a large extracellular structure (**A-D**). The structure fluoresces positively for the *Hrr. lacusprofundi* specific probe (**B**) but not for the Nha-C probe (**D**) or for the non-specific DAPI nucleic acid stain (**C**), indicating that it is likely of host origin (i.e. *Hrr. lacusprofundi* rRNA, possibly ribosomes) and that there is a barrier between it and the main Nha-C cytoplasm. The structure may be similar to those seen using TEM (Fig. S15). Cy5 Nha-C specific probe (red fluorescence); Cy3 *Hrr. lacusprofundi* specific probe (yellow fluorescence, recoloured to green to improve contrast); all nucleic-acid containing cells stained with DAPI (blue fluorescence). Composite image of all three filters (**A**). Individual filters for Cy3 (**B**), DAPI (**C**), Cy5 (**D**). Scale bars represent 2 µm.

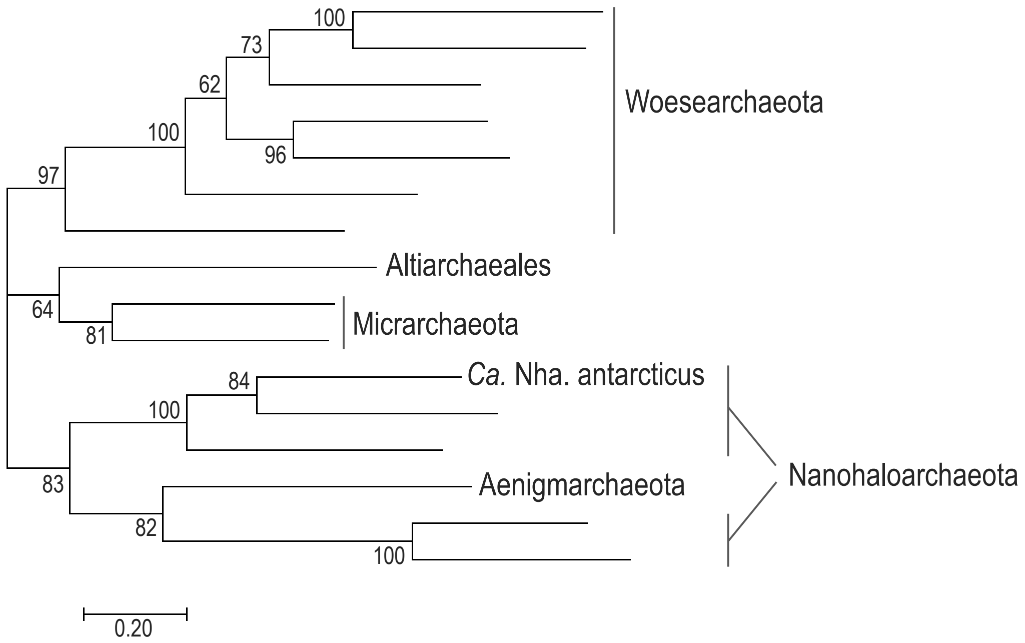

Fig. S17. Phylogenetic tree of the predicted intracellular region of archaeal SPEARE proteins. The predicted intracellular region contains the restriction enzyme domain (Fig. 7). The evolutionary history was inferred using the Maximum Likelihood method based on the JTT matrix-based model (6). The tree was drawn to scale, with branch lengths measured in the number of substitutions per site. The analysis involved 16 protein sequences with a total of 719 positions in the final dataset (Table S3). Evolutionary analyses were conducted in MEGA7 (7).

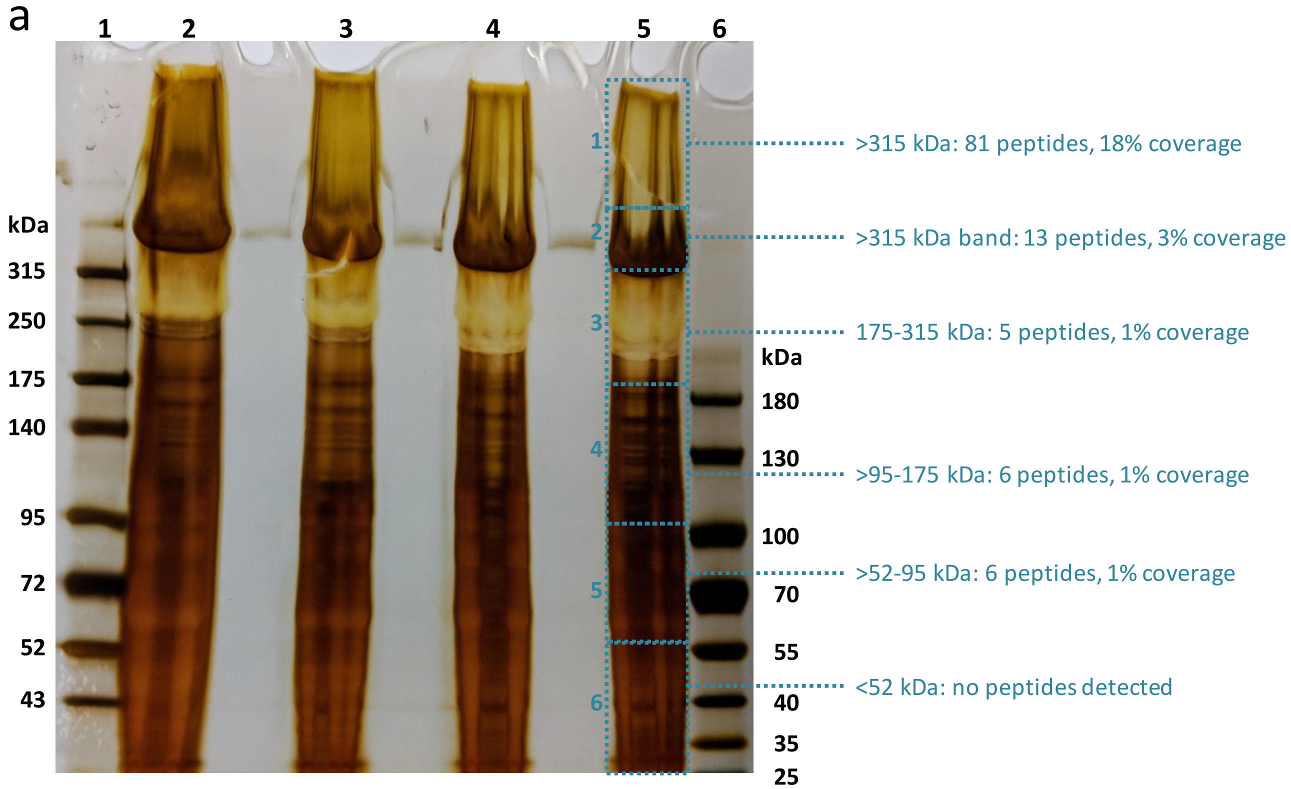
a
1
2
3
4
5
6
kDa
315
250
175
140
95
72
52
43
kDa
180
130
100
70
55
40
35
25
1
2
3
4
5
6
>315 kDa: 81 peptides, 18% coverage
>315 kDa band: 13 peptides, 3% coverage
175-315 kDa: 5 peptides, 1% coverage
>95-175 kDa: 6 peptides, 1% coverage
>52-95 kDa: 6 peptides, 1% coverage
<52 kDa: no peptides detected

b

VPFISTSTALTCDAGTVDSTCVINTTHDVSTETIIGTGSIEVREGGTLTTDDGVANLATFELDGEFYINDTVNGNFDITASDLEITENGQLR
SDSRGFSGGARYEDGEGPAGGDNPGNDGAGGGHGGFGGRTDLDSSKQPYGSYMYPTTAGSGGGGSNQRNGGDGGGILTVNVSNSIIVNGPIS
ANGANGGSRWSRDAGGGAGGSILLEGDTITGSAPISSQGGNSGGGGGGGGSGGRIAFHVTTNNYNGEISVDPGSGQDADKGTIYPQTESDIC
TSGSLSTTCAVQEQVNLINNLSDTGDLVISSGGEILVYEKTDIDLAEVDIRSGGTIKVI**RDIDLTGTNLFKV**NGVLDTTGENNFDVNQFEVS
NDGQVTTTQQVEFENTDLVDIIGSLTTQQGVDFKNTDQISVPGTLDAQTDIEATSASNIDIAGTLTTQNGGINFENADELIVSGVLEAKNTI
NANSATNIDIEGDITTEGLIDLRLAKTGTEIDLSNTALLEGEKVNITSENFRLGLDSLISVDGFGYSGGSTGSNGEGPGNGFFVTASDYGSG
AGYGGEGGDDADGDTGGSTYGTIEKPTQPGSGGGGSNSQPGSAGGGVIFGNVTNDIVIDGTISADASDSNCGAGAGSGGAIWLNAGNLSGTG
SVTANGGDSCGGGGSGGAG**RIALDTEIDNYQGSVFTNGGIGGNEAGKA**GSIYPRDFICEEGNLDSTCTITSSHAINSLEGNNLIVDGTEIYS
PAMGMDLNFTDITLVSSGKITTSRDLNLIETLRVTLNTADLEVGNNLNWDSSGVLFDLSDSLVDVENIVDIELNSTDSVIELESSSEIKSSR
INLTAENVSVNSQNTLNTRGLGFSGGDSSEPGSGPGSGVYGDPDDGTGASFGGLGGTDAQGDPVEPTYGSLIQPTQFGSGGGGSSNRKGGNG
GGAIFINSTNELALGGKLTSRGQDPSNNNNYAGAGSGGSIWIVADLFSGNGEINAEGGTAFNQGGGGGGGRVAFNVTT**RTFSGLTTVNGGSG**
NEFGEPGTIYPRNNICNEGTKDTTCRITSSIQLNQLEGAGTLELRNNAEIYAERSDIELNFSTIDIESGS**KITAPQKLNLAGTKTFINRGDI**
NAQGTISLENAERIRSSNIIESEEAIILENISDVEIASNKLQAPTINVTGQNLTLSQSGTLSTNYDGFAGGESNTDGSGPGGGSYVEGDDGS
GGGYG**RNGGDTDEGQPGGFAYGNPDEPIQFGSGGAGSSNRKG**GNGGGSIFVNISDTITVDGQITSNGENGQCNSNNGAGGGSGGSIWIITES
ITGSTDGSITSNGGEGGCDGGGGSGGRISIESISESYDGVVQTNGGTSSYEYGSPGTVRPKDYLCDVGYLSTTCEINSYHHVQELKGNTLTV
STGTLFSDTNPMDLNFSSITIQNNGVIESKSDITINTTDKITVDGVIDTQQ**KLTVNSGDTLENRGNINAKDAFLQPSRVITQSNGAYSIIDL**
LSSKNTDEFTNDGNIDASTVNVSSSTISVNGEINTDGKGFPGGNDDQDGSGPGGGIYPGSQDGSGGSHGGIGAPNSYGDDPIPPVYGSLTYP
SEFGSGGSGSNKKPGGNGGGIIHLYSENGIAIDGTISADGSQAQQTYGQGGGGGAGGSIYF**KSDNLAGTGMVTANGGNGRS**GGGGGGAGG**RIA**
ADTTNNFDGNMYAKGGTGGPNGAPGTIHPQNYICDTGTLSTTCTINTDHETGRLNGTGTL**RLSDGNSIRN**SISSINISGFEKVELLGSSSIG
SNYEGVEFNVTTLDLSSNAQIDTETSAKFTGIDTFNLDGNVESTNNISVS**RVQNFNSNGQITARL**LEGIDISSFIIDGSITTDRTNISAVSG
EITSNG**KIDTTDRGFKAGGSYSDGTGPGKGLRG**SGNDASGGAHGGIGGIDSQGDIQTNTYGSLTQPTQFGSSGGRGKRSGGNGGGAIFINTS
NTLTLNGNLLANGGNAGCRHRVNGGGGGAGGSIWTIADTLEGTGDMEANGGRGSCGGGDGSGG**RIALQVENDGYDGSTFVNSGDAYRY**AEAG
TVYPDDFLCDQGNTSTTCTITENHHIQEMSGTGQLVVDDTGTLFSGNGTLNFDFSRIDVNGKIETYQDTGNGIDLKETETLIVRGSLLSDES
LNATELESFEVKDGLVDVQKIVDIDAGNIDINGDVLGQKIFFDSARDIIV**RAGNEISTNFRGFSGGARYEDGEGPGGGTYGGGNRGGAGGGY**
GGQGGDSSNSDGGIAYGTFASPDSMGSGGAGGRSTEGGAGGGFIKLRTEDDLQIDGTVSANGEGTSGCCD**RGGGGSGGGIFLSSDSLTGAGE**
ILSQGGDAGTGQGETGGGGGGRIVLY**RSSENFFGDIRV**DGGTGDFQNGEDGSIYTSPTDDVLGRLNVTL**KDAEGNPISGKPVELKIAGESAT**
ACSYETDLTGKMSCLIDTTEDYDINVQLFSSDIQGRIYRDIEAPQDIVIQENTDISVKDVVSGEFLQDQTVTVYSKDTGNIIAQGETLGIFE
NFEDGEEQESLTDNTGWIQNSKDLLYNKEESAIGDTSVYTGDLNTGTPLSHKEIYPDNRKPEVIETWY**REGSSSEGFGMTLRDENNNRV**LGF
GSDGPNFFVYDDANFNRNLCSSEAETWYKVNLTIDWESETFDFERSDGCTGTNFEL**RHTDGFQSIYVENQNGGNWGSSRINTWIDGIRI**KPN
KGAEGITFALEDEEYRV**RITGDGIETTDRDLFRPDNFQAEVKN**LNSSTLCEQTGDIQVSGECTITPGEYNV**RSFQLLETGSIDIEAAPSQDS**
ASSYGGVKIVSNGEIEILGDIDGAQEGYSTESGPGAGGSNEDGNSGAGYGGPGGDGGSGIGAPGGSAYGQDSEAT**RLGSGGGDNNGGFGGGS**
IWLESKSNI**RVDGSINVDAEDGTQDAGGGSGGSIRMESPAVSGSGTLLARG**GDGGDNTNENKSGAGGGSGGRLAIYSLEGGVSNFQVNLSGG
KGGIGNETSGQDGEDGPTGTTYTGTITDPEINISLEDASGNQVTEDAGLTIY**RTGTPYEECSSTPSDTGRFLCDVDPGQRYDIATAGFGGSA**
RTLYNVSVPLETTIKENFNVSILDTNGNPVEGTDVYVKDNETNEIQCEGTTDEEGLMGCAVPEGSSYDVAVGEKNWTNSFDNELPGSVEIRD
LFSLNILADEQRGLINRFPENILFTVYQTNSDVPQCQGSTNNVG**RLGCSLDTNSLYDIGLTKYNANDDDRSYHPYSLVAKNVGVPQNRNIEI**
KDYLLEVKSVGSTGASQSDTELNVFPQPLGGLACSGTTNDKGLLYCGLDNDQKYQI**KNADGLLERL**LVNIPQDTVVQNTPANELINVSFQDE
LGGPLTDLRFRIDRGPNSNACEGVTGPSGNASCGISTGQYNL**KIGDEIAKTDVESGNNYLIKRH**FNH**RLNSVGSYDTGEFRVTGRI**WFDEDL
GNFNENIDFWVDGE**KITSTSTGSDGTFDKRF**VYEEPGTHTLTLNYTDKYGLSDERKVTFGVTDKDQGIELINLTVEDGQEPVYQTVDVTGGT
SLTAYPDMDGNITLALDSNNFYNFVSPLRNVSNIQPTADIPITPYAEVFV**RDSNGDPVSGQRV**LAT**RAGQTEILCDETSDSDGRAFCKL**TSG
EDYDIKLNSQTGRVIA**KAQSPIQVTTGTSSYRGSIKYQFLKNNRELGDEIVKYLTGTTGERV**CRGKTTSNGNFFCQPILDS**KYDVVTSGYTG**
REIDVKKGLDPNFGDPITFDPGTNIHLVDITGNDVPSLEATVYETGTDTKACEGFTNSTGRLVCDLDESKKYDIKTSQAVERYEMQPPEETV
VQVASFFNNKMLNLTLKGPDQNNLTDTDI**KVTNRDENGVVACRG**KTNDKGFFSCGIDRNDRYDISSEGQPLLANVEVPNQLDLGRNLEIKAV
DSTGSVVQNAAFQAYNVQNDLLACSGQTNSEGILRCGLERDKEY**RIESPNGVERY**RVKMPE**RTTIEYSDDNEPVNMTFRN**STDQ**RMIDEGRI**
VIKNQEQIEVCSGDTNKLGEFSCGLNSSGTYQIETDLYNTKRQTIKADLATSNQYTIKPNVNVSLTNYTGAPATGNIKLVDNQTEATACYGE
TDQNGQLTCALEEDQTYNIIPYYNQISEGYTANITAPGEKALETIKFTTYDPDAPPGDGAVGINESVEIRNNQRALCTGYTGESSSLYCGTV
PGESYSIDNPVKNVQDIMSNNSLQVGYGQTDLEVTALDIPDFGITDANFTY**KVEVYNPGPRF**AYDIDVNATASGSGWQGY**RTVEGSLAPGER**
RNVSIEVFVPDGTVGTVKSIETQTNWTNPDLTSDSITDTGTIDVVGTGRLTTNSTIESILGEGSDFTKIGEVKLGSVGNEEALANYNVDGPE
SWEFGFDPFPSPLVEIPIGDTKNISVFAKIPESELQGTYNATLNIFEESRKDDEANITVYVPPSAEFE**RTPDSLGTIDAPLGTSGDVGTIDV**
KNTGNQNLTFTVN**KEIGEGSSTFVKRG**LTTFELEPLEEREVDITYQVPADSTGGEYEYNITLSCSPGCGDKKTDLTLDVRDLPPSIDNYSFS
PKFLEPSETIEWNINASDNDRIDRVWVDILGQENITAETAGELTGNPFYTANFTPQEESALYDF**RIYAQDSGDLTTRTDVKS**FQVIPQTDVE
TTLTQTVFTMPDVTVDSGDTVNFNATFTNNGDQKAYNVSYEMQLPDNFSSSTPNLNVGDLESGESKTTELEVTVSAGVSPSVYLPTI**RTDWE**
NADESTDSSEISFKADVLENIDLRSNISTQLTEMEQDSVREFNITLSSEGNEPINDINGECASGDGCENLFYTFFLPDRIPVGEQRQAKVRV
EADLGTPDGQYDSNIEIGGEGDIAELSNAVTVNKDLSMELSTNNYSQRVGPGTQIALENLSIRNNGNQPLEVAMPESSNIEFDPTGYDLDIR
NTKESSIELTAPETPGDYEYNLTVAGFDRETFERIE**RFVEFSIRV**FDYSIDLENVENDTEITDGEEITLTTNVEFEGSSVTSDMDWDVEIGN
KEADVVSTNYNSGQGVWEVNVEAPDIEQGNRYDLEVQGTYDSEEITTASAYTDRIEYADLEAPVYTDVNAEDIEESGSSRISL**RAQDKGEIE**
SVRDINATIETPGGTNLTLALEESGDQWVVNTPEFDQEGYYEVYLTSSDTAGNMKTERTFFQLYETDNIQGEIKTPEGEGLDIEVTLQKPDT
KEEIDQFYTSNGNYSRSVKSGTYDVVLDINNSQMVISLQDVPHEELNDPLDMDPFIPNAYVDVPISSNDNELTGVAIKT**KSLQDHPATVGIS**
YSGYIDQIKNVRDLGIYKCDSWSFENQQEGCGSDWTKKDTAVSPATETVFVSVDSFSSFYLTEFGGSDEEENVTAGDGSTGNSTEENGTNQD
IVDAIEGLDTGGGGGGGGGGIGDREFEEGIESLLGALNDTEENDTLQFSGE**RIVVEVAPGQRKT**ASISVSNNIRYNQTLDIGLTGDIQ**RFVD**
IEDEVRIDGNSQRDIELLIAAAEDTETGSYSGFVTIEGEAVSKQMPVNIEVVEPAETDRDLLDLEIETVVDNIVPGENLRI**KTSMLNQGYTR**
NVDVDLNISVRDPDTGDIINRKTTTVAVSTTLDKIFEIKIPEDIELKRYEVEASVSYSNIEPPQTANAIQTFVVDKPFWDKPLFGMPRKDIA
GSMIIFLFLSLASYSGYLYYRKKLLENQ**RYQEDLDVSTLPSGGSRQ**AHLGKLAEHGS**RSFLELEEMTTHTLIAGATGSGKTVTGQGIVEEAL**
KEGTNVIVLDPTAQWSGFLRESNETSMLEKYSDYGMSKNEARGFDGNIRAIEPGEELDITEYLETDDEGQIIVFSMHKLDSDRIDEFVDTTI
QQVFDANLPESGQLDTLIVYDEVHRLLEKFGGSGKGLKQLERGAREFRKWGVGMILLSQVISDFSGEIRANIGTTIQM**RSQYEGDLKRM**ENK
YGMDTVKSIVKANVGSGMLQNSDYNHGKPYFVDFRPLLHSPHRLSDEKLEKYEHYNQAIDTIKTEIEKREEKGEDMFELKSELRLAKKNLRK
GSFNLVKIYIKEIKENLN

Fig. S18. Gel separation and mass spectrometry analysis of *Ca.* Nha. antarcticus SPEARE protein. (**A**) 3-8% Tris-acetate gel, run at 150 V for 1 h with replicate samples of Nha-C protein preparations. A single lane was left empty between loaded protein preparations. Lane 5 was cut into 6 size fractions and proteins extracted from the gel pieces used for mass spectrometry analysis. For each excised piece of gel, data is provided for the molecular weight size range of the excised fraction, the number of unique peptides matching to the *Ca.* Nha. antarcticus SPEARE protein, and the percent sequence coverage the number of peptides represents. High molecular weight marker (Cell Signaling) (lane 1); 34 μg Nha-C protein (lane 2); 23 μg Nha-C protein (lane 3); 43 μg Nha-C protein (lane 4); 34 μg Nha-C protein (lane 5); PageRuler pre-stained protein marker (Thermo Fisher) (lane 6). Molecular weight (kDa) of proteins in the size standards are shown flanking lanes 1 and 6. (**B**) *Ca.* Nha. antarcticus SPEARE protein sequence showing location of peptides identified by mass spectrometry. Gel sections 1-6 shown in panel A. Peptides identified in the >315 kDa sections 1 and 2 (red font); peptides identified in the <315 kDa sections 3-5 (green font); TMD (grey shading).

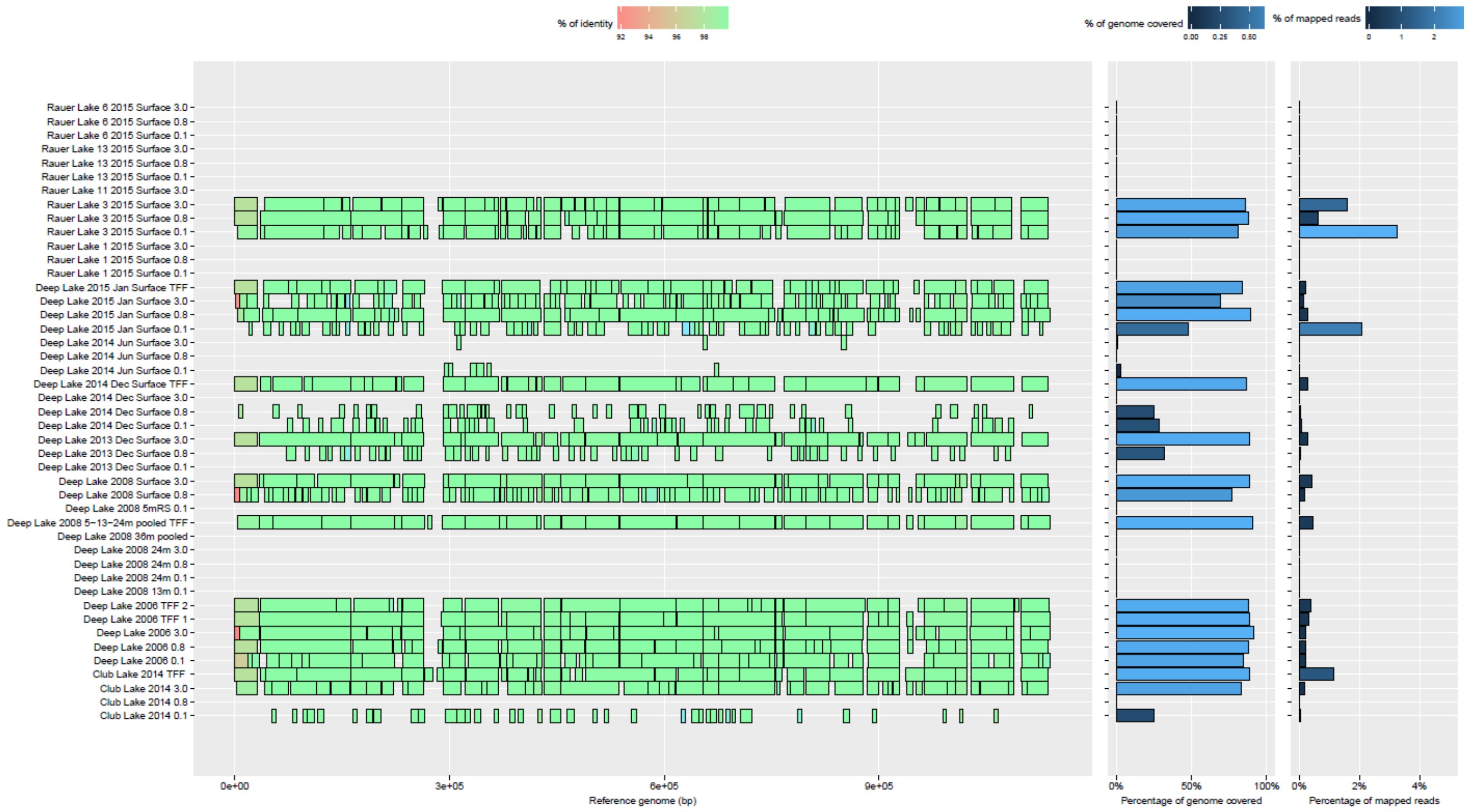

Fig. S19. Contig recruitment of Antarctic metagenome assemblies to Nha-Ce. Contigs from metagenomic assemblies across a range of sampled Antarctic hypersaline systems were aligned to the Nha-Ce contig (Ga0309993_1030). Shown is the level of sequence identity (colour coded), calculated genome coverage and total percentage of reads that mapped to the Nha-Ce MAG.

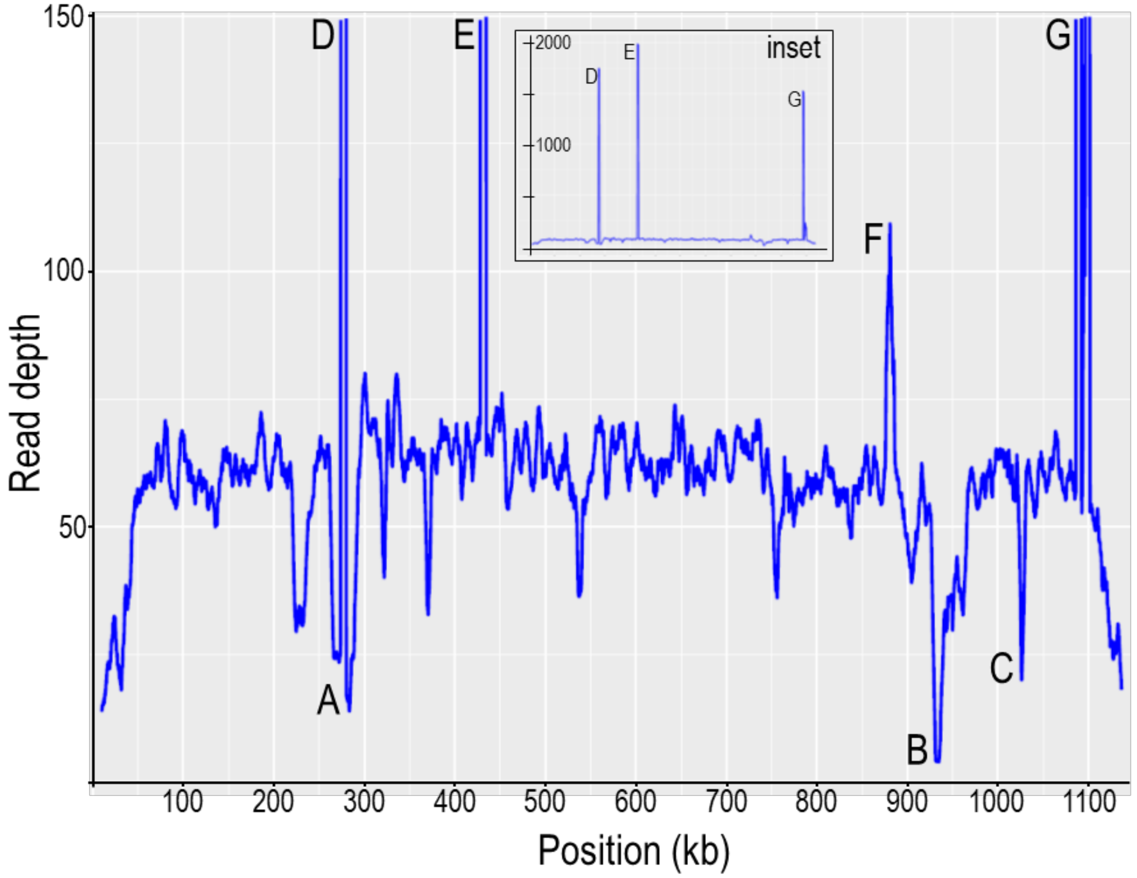

Fig. S20. Read recruitment plot for the Club Lake metagenome. Reads from metagenomic sequencing of Club Lake were mapped to the Nha-Ce contig (Ga0309993_1030) using bowtie2 to assess heterogeneity of the Nha-C population of the lake. Regions of low recruitment (a-c) represent a transposon, integrated virus, and region of putative horizontal gene transfer, respectively. Type 5 BREX system that is absent in Nha-CHl but present in Nha-R1 (c). Regions of high read recruitment (d-g) contain similar elements. Insert (i) shows the recruitment plot scaled to show the full size of peaks.

a

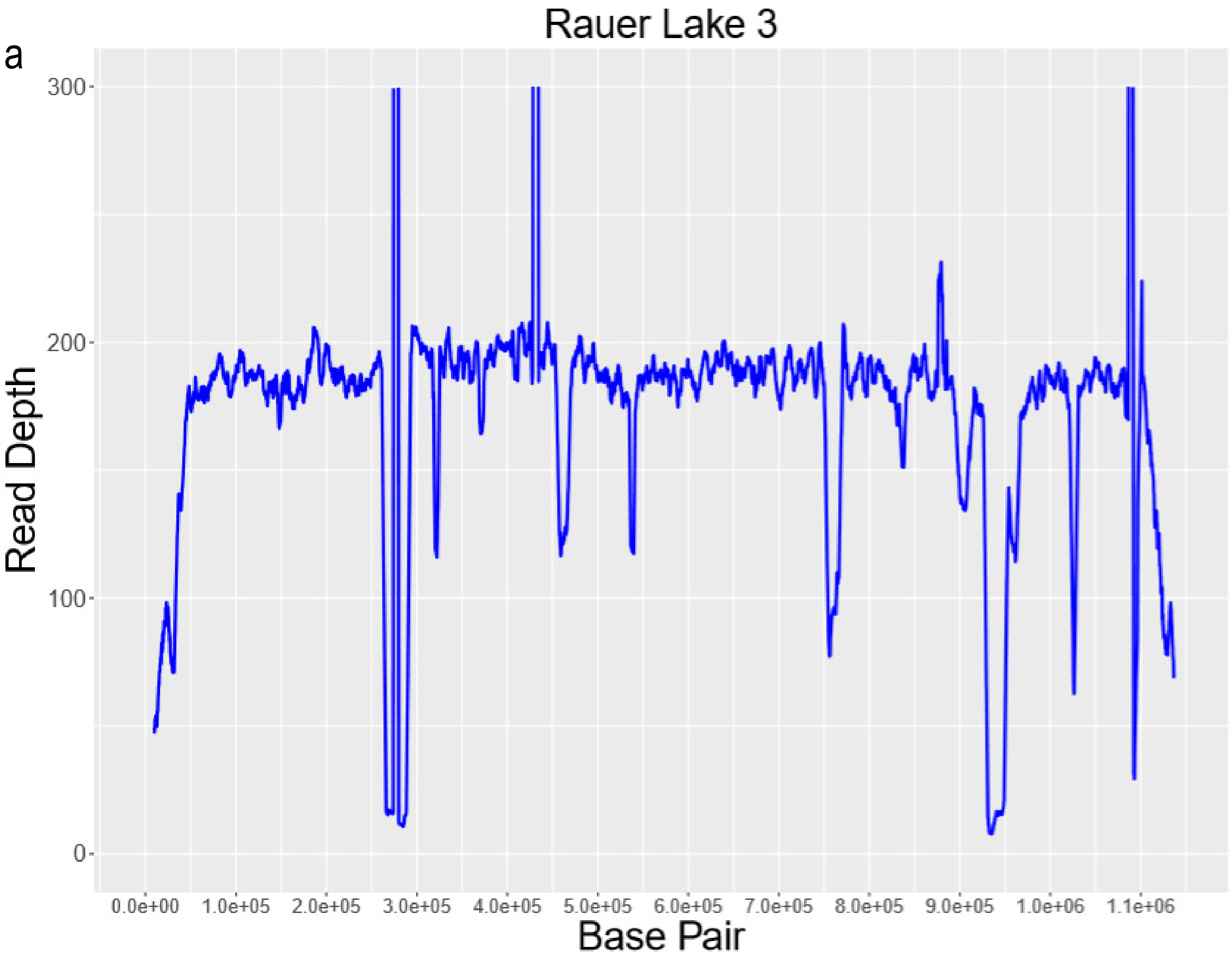

Rauer Lake 3
Read Depth
300
200
100
0
Base Pair
0.0e+00
1.0e+05
2.0e+05
3.0e+05
4.0e+05
5.0e+05
6.0e+05
7.0e+05
8.0e+05
9.0e+05
1.0e+06
1.1e+06

b

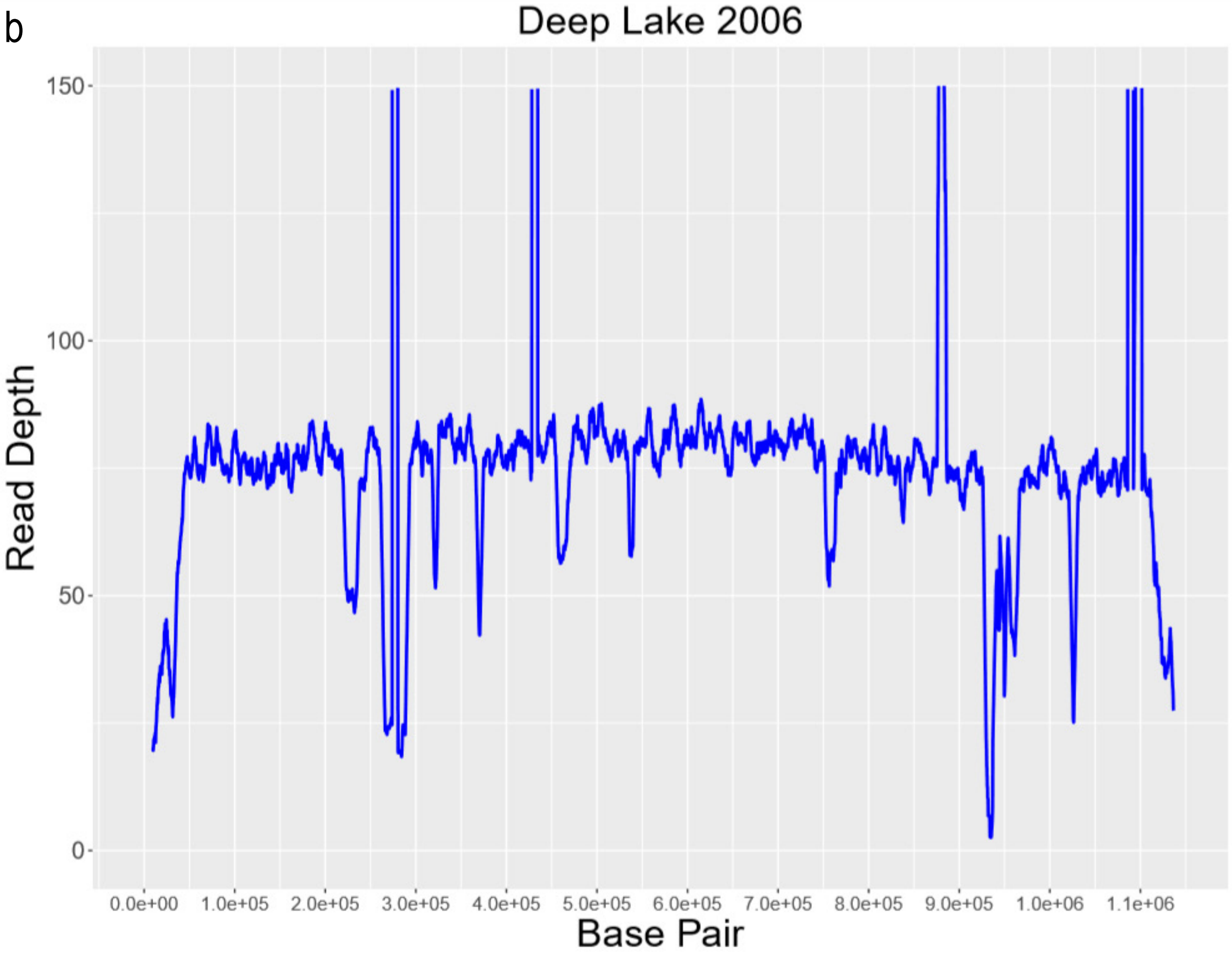
Deep Lake 2006
150
100
50
0
Read Depth
0.0e+00
1.0e+05
2.0e+05
3.0e+05
4.0e+05
5.0e+05
6.0e+05
7.0e+05
8.0e+05
9.0e+05
1.0e+06
1.1e+06
Base Pair

c

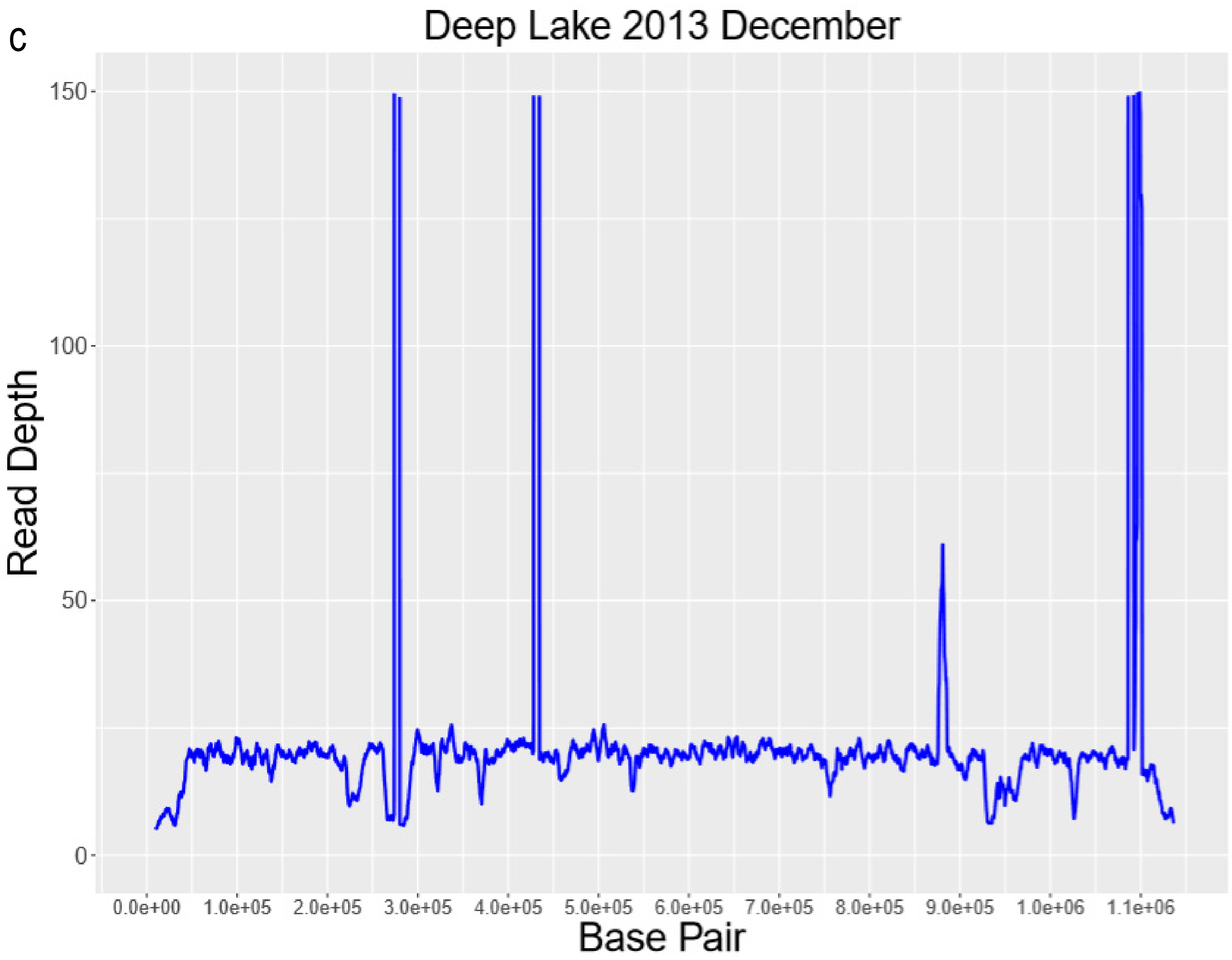

Deep Lake 2013 December
Read Depth
150
100
50
0
0.0e+00
1.0e+05
2.0e+05
3.0e+05
4.0e+05
5.0e+05
6.0e+05
7.0e+05
8.0e+05
9.0e+05
1.0e+06
1.1e+06
Base Pair

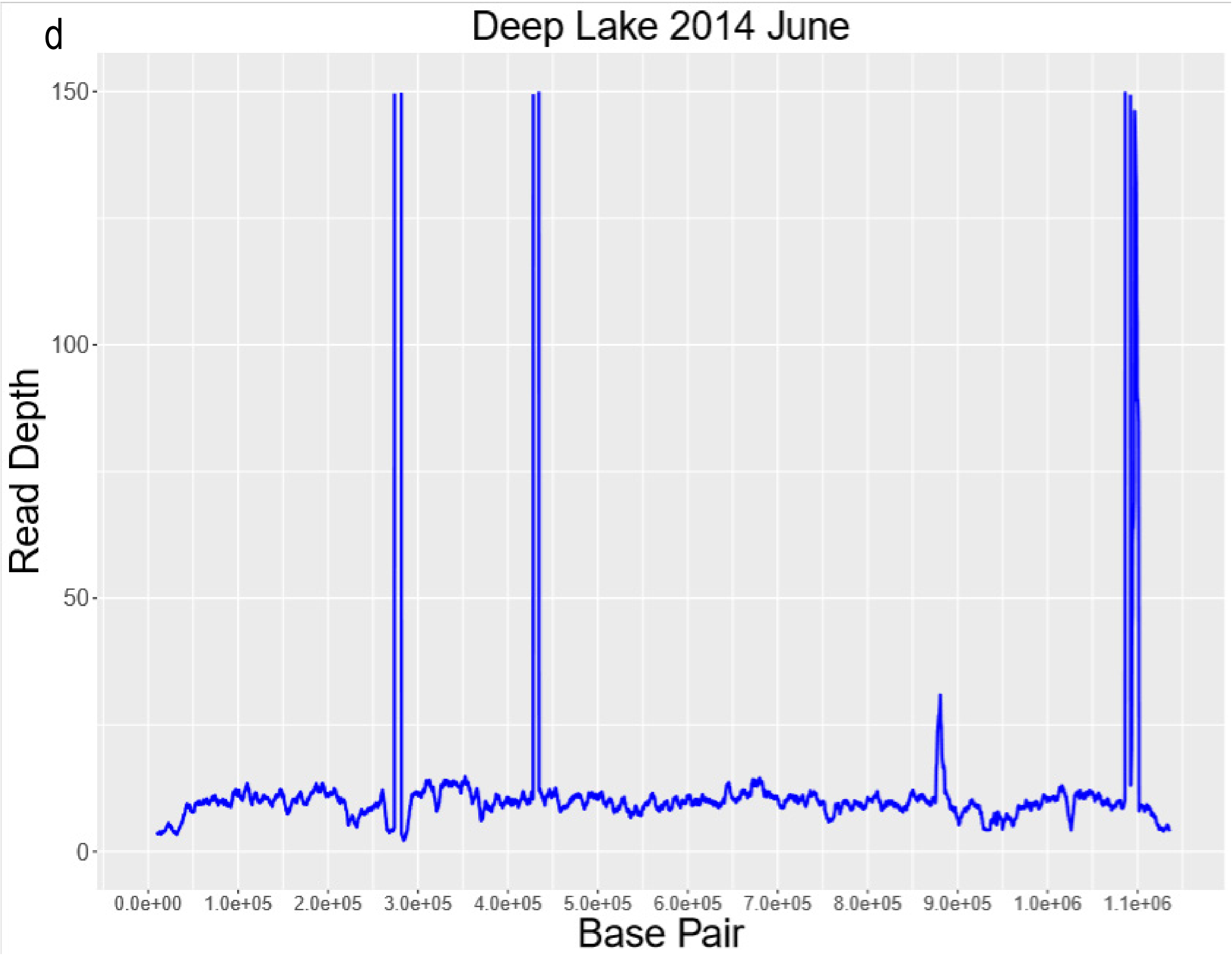
d
Deep Lake 2014 June
Read Depth
150
100
50
0
0.0e+00
1.0e+05
2.0e+05
3.0e+05
4.0e+05
5.0e+05
6.0e+05
7.0e+05
8.0e+05
9.0e+05
1.0e+06
1.1e+06
Base Pair

e

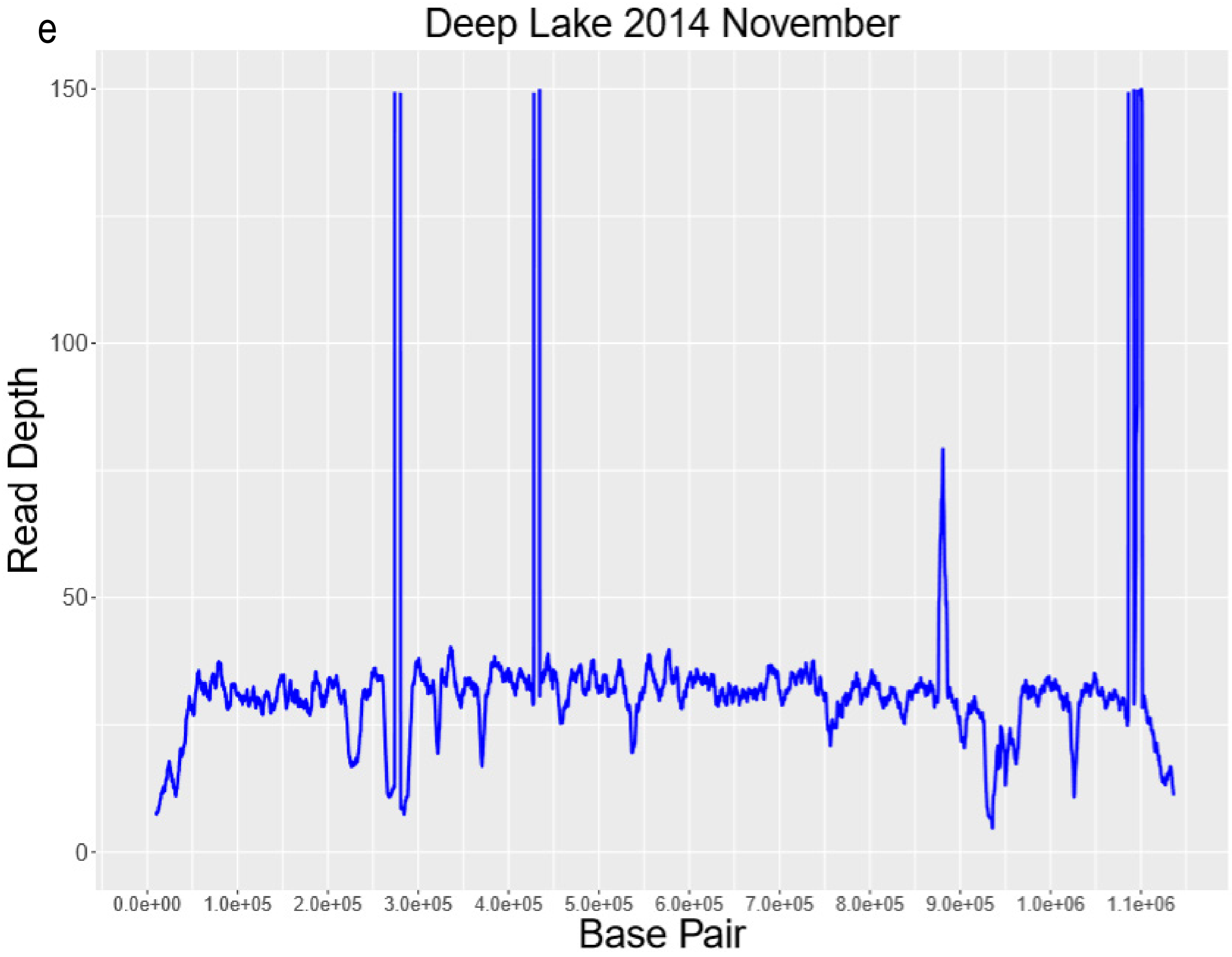

Deep Lake 2014 November
Read Depth
150
100
50
0
0.0e+00
1.0e+05
2.0e+05
3.0e+05
4.0e+05
5.0e+05
6.0e+05
7.0e+05
8.0e+05
9.0e+05
1.0e+06
1.1e+06
Base Pair

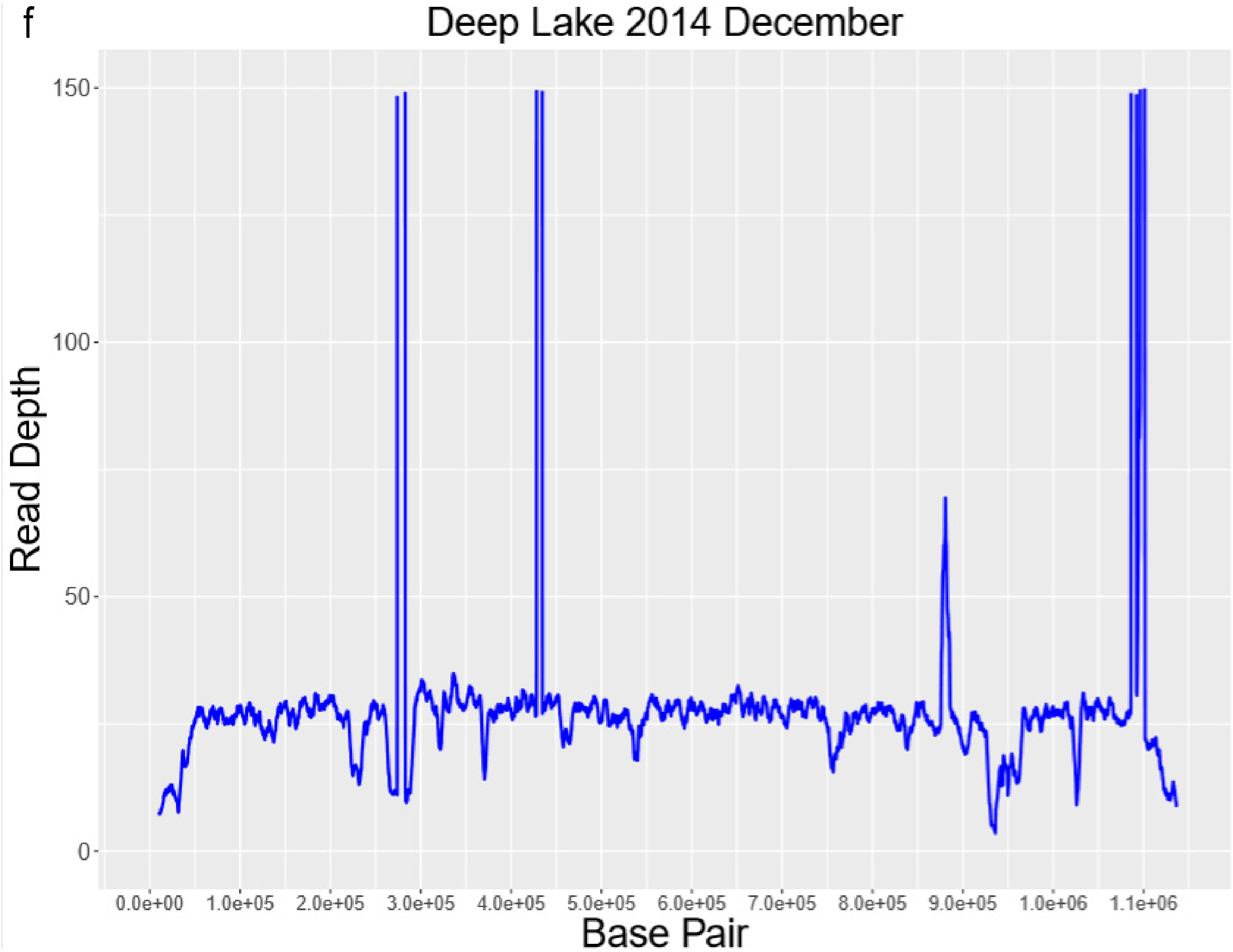

Fig. S21. Read mapping of Antarctic metagenomes to Nha-Ce. Reads from six pooled Antarctic hypersaline metagenomes (8) which contained *Ca.* Nha. antarcticus (Rauer 3 Lake, Deep Lake time series) were mapped onto the Nha-Ce MAG (**A-F**). The plots show the coverage (y-axis) across the length of the Nha-Ce contig (Ga0309993_1030) (x-axis). For Club Lake, see Fig. S20.

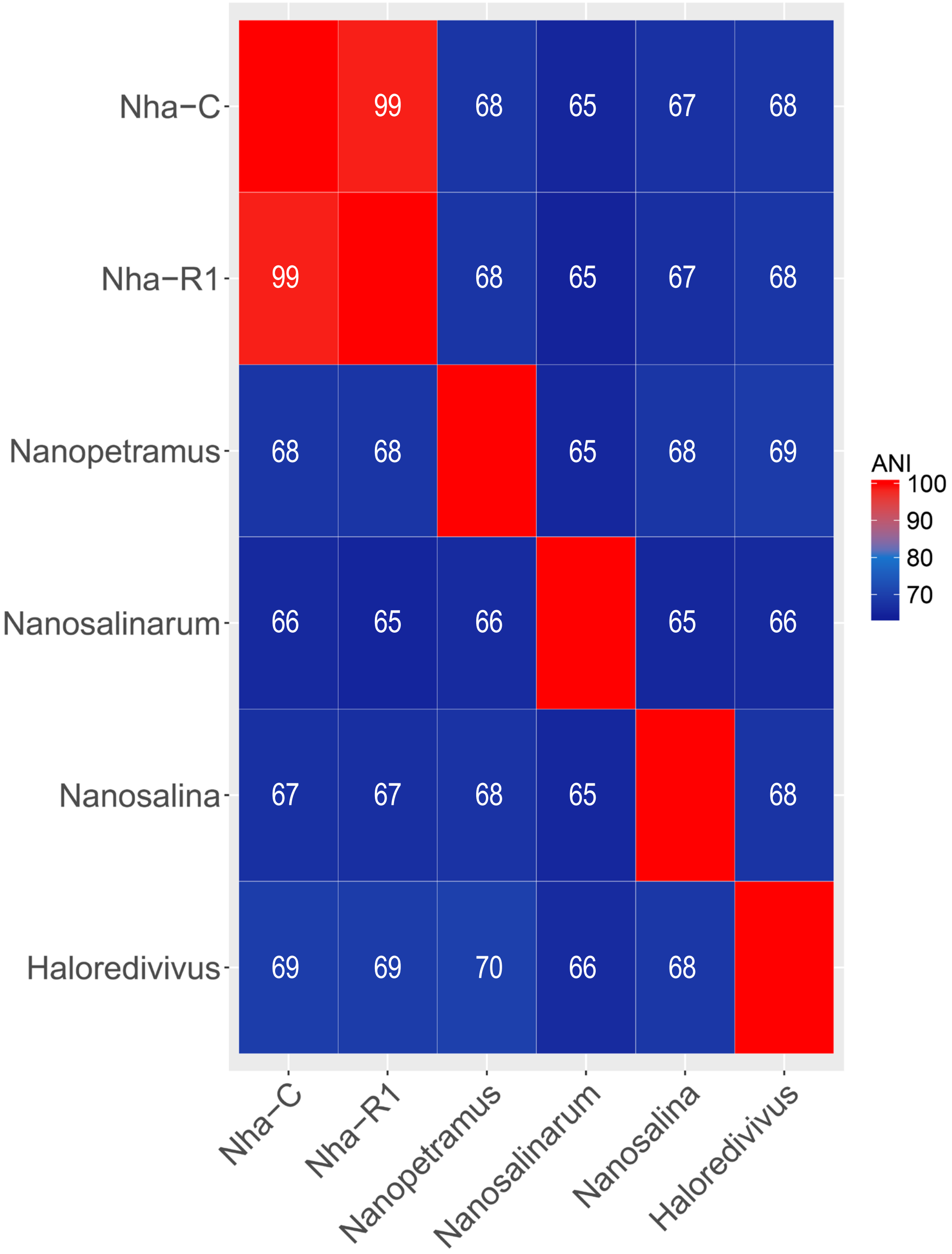

Fig. S22. ANI heatmap of nanohaloarchaeal MAGs. ANI comparisons calculated using the JSpeciesWS ANIb tool (9) shows high level sequence identity (>99% ANI) between the two *Ca.* Nha. antarcticus strains. All other nanohaloarchaeal MAGs had much lower (≤70%) ANI suggesting each MAG represents a distinct genus of Nanohaloarchaeota; the proposal for distinct genera is supported by their 16S rRNA gene identity (<90%). The Nanohaloarchaeota are from diverse types of hypersaline environments: marine-derived lakes (Nha-R1 and Nha-C), thallasohaline lake (*Ca.* Nanosalina, *Ca.* Nanosalinarum), saltern (*Ca.* Haloredivivus), halite (*Ca.* Nanopetramus). Numbers within the heatmap (white font) represent pairwise ANI percentage.

Ca. Nha. *antarcticus*	**CGAATGCTCTGGGATACAC**
Hrr. lacusprofundi	**CGAATGCGCTGGGCAACAC**
Natrinema	**CGAATGTGCTGGGCGACAC**

Hrr. lacusprofundi	**GGACATCTCACCAGCGTCGACTGTAATAA**
Natrinema	**GGACATCTCACCAGCATCGACAATGTGCA**
Ca. Nha. *antarcticus*	**GCGCACCCTACCAGAGCCGACAGCAGAAT**

Fig. S23. Primers design for FISH probes. Sequence targeted by FISH probes (i.e. reverse complement of the probe sequence; Table S6). The top underlined sequence is the target and red font shows mismatches to the specified organisms.

Table S1. Metagenomes of enrichment cultures and MAGs of *Ca.* Nha. antarcticus generated in this study.

IMG ID	Metagenome / MAG	Sequencing method and assembly method	Number of contigs and size of MAG	Average read depth of taxa	Primary use in this study
3300005925	Metagenome: Rauer 1 Lake enrichment (includes contigs for Nha-R1)	Illumina; Megahit 1.0.6	28529 contigs; 32.21 Mbp	Halorubrum 670<br>Nanohaloarchaeota 980<br>Natrinema 20<br>Other archaea 540<br>Bacteria 188	Relative taxonomic abundances in enrichment metagenome; Closing gap in Nha-R1 MAG.
2643221421	MAG: Nha-R1	As for the metagenome except assembled with SPADES 3.5.0	2 contigs*; 1.01 Mbp<br>*Note scaffold 2 and 3 were manually joined removing 5.8kb from one scaffold and showing they are contiguous with no intervening sequences missing in the metagenome data.	N/A	Genomic analysis; proteomics
3300028663	Metagenome: Club Lake enrichment (includes contigs for Nha-Ce)	Nanopore; minimap, miniasm, racon	572 contigs; 60.30 Mbp, Nha-Ce MAG: 7 contigs, manually reduced to 1 contig; 1.14 Mbp	Halorubrum 260<br>Nanohaloarchaeota 85<br>Natrinema 60<br>Other archaea 35	Relative taxonomic abundances in enrichment metagenome; Lake metagenome read recruitments
3300028522	Metagenome: Club Lake enrichment grown with *Hrr. lacusprofundi* ACAM34 (includes contigs for Nha-CHl)	Nanopore; minimap, miniasm, racon	169 contigs; 29.40 Mbp	Nanohaloarchaeota 475<br>Halorubrum 890<br>Natrinema 130<br>Marinobacter 90	Relative taxonomic abundances in enrichment metagenome; MAG closure
2791354821	MAG: Nha-CHl	From the metagenome, plus manual concatenatation of two contigs	1 contig; 1.09 Mbp	N/A	ANI
3300031914	Metagenome: Club Lake enrichment grown with *Hrr. lacusprofundi* ACAM34, harvested at stationary phase	As for metagenome except assembled with SPADES 3.5.0	17341 contigs; 34.9 Mbp	Nanohaloarchaeota 780<br>Halorubrum 480<br>Natrinema 260<br>Other 130	Relative taxonomic abundances in enrichment metagenome

Table S2. Domain annotations of the nanohaloarchaeal SPEARE proteins. Protein sequences were analysed using a combination of I-Tasser, Interproscan, and TMHMM. Transmembrane Domain: TMD

I-Tasser										Interproscan			TMHMM			
Nha-R1 - 2643306914																
AA range	Model C-score	TM-Score	RMSD	PDB Homolog	Function	TM-Score	RMSD	Identity	Coverage	AA range	Domain	Function	Number of TMD's	Intracellular AAs	TMD AAs	Extracellular AAs
0-1500	-1.32	.55+-.15	12.2+-4.4	5j44A	Protease	0.874	1.36	0.091	0.886	77-262	PTHR31513	Unknown	1	5634-5998	5611-5633	1-5610
1500-3000	-0.16	0.69+-0.12	10.2-+4.6	4iglA	BC Toxin	0.946	1.22	0.097	0.954	324-696	PTHR31513	Unknown				
3000-4500	-1.02	.59+-.14	12.4+-4.3	4o9xA	Tcc Toxin	0.932	1.7	0.109	0.947	814-1038	PTHR31513	Unknown				
4500-5998	-0.02	0.71+-0.12	9.8+-4.6	2b39A	C3 complement protein	0.94	1.46	0.092	0.951	1134-1363	PTHR31513	Unknown				
										1480-1698	PTHR31513	Unknown				
										1817-2006	PTHR31513	Unknown				
										2143-2355	PTHR31513	Unknown				
										2720-2965	PTHR31513	Unknown				
										4616-4683	PF10633	Unknown, asscoiated with galactosidases				
										5611-5633	TMHelix	Transmembrane Helix				
										5652-5896	SSF52540	P-loop containing nucleoside triphosphate hydrolase				
										5677-5825	PF04581	Restriction Enzyme Type III				

Nanopetramus - AOV94740.1

AA range	Model C-score	TM-Score	RMSD	PDB Homolog	Function	TM-Score	RMSD	Identity	Coverage	AA range	Domain	Function	Number of TMD's	Intracellular AAs	TMD AAs	Extracellular AAs
0-1500	-1.48	.53+-.15	13.6+-4.0	4iglA	BC Toxin	0.898	2	0.11	0.915	195-227	SM00564	Pyrrolo-quinoline quinone beta-propeller repeat	0	N/A	N/A	1-8553
1500-3000	-0.69	.63+-.14	11.6+-4.5	4iglA	BC Toxin	0.937	1.36	0.089	0.946	995-1025	SM00564	Pyrrolo-quinoline quinone beta-propeller repeat				
3000-4500	-0.56	.64+-.13	11.2+-4.6	4iglA	BC Toxin	0.914	1.41	0.1	0.923	1040-1069	SM00564	Pyrrolo-quinoline quinone beta-propeller repeat				
4500-6000	-0.18	.69+-.12	10.3+-4.6	4iglA	BC Toxin	0.917	1.06	0.082	0.923	1085-1115	SM00564	Pyrrolo-quinoline quinone beta-propeller repeat				
6000-7500	-1.6	.52+-.15	14+-3.9	4o9xA	Tcc Toxin	0.93	1.91	0.09	0.948	4421-4650	SSF49899	Concanavalin A-like lectins/glucanases				
7500-8500	-0.75	.62+-.14	10.8+-4.6	3ja4A	Viral RNA-RNA polymerase	0.931	1.8	0.091	0.944	8208-8464	SSF52540	P-Loop containing nucleoside triphosphate hydrolase				
										8216-8361	PF01935	Unknown				
										8233-8433	SM00382	AAA-ATPase				

Nanopetramus - AOV95205.1

AA range	Model C-score	TM-Score	RMSD	PDB Homolog	Function	TM-Score	RMSD	Identity	Coverage	AA range	Domain	Function	Number of TMD's	Intracellular AAs	TMD AAs	Extracellular AAs
0-1500	-0.7	.62+-.14	11.6+-4.5	4iglA	BC Toxin	0.915	1.34	0.099	0.925	238-437	SSF049899	Concanavalin A-like lectins/glucanases	1	2941-3315	2921-2940	1-2920
1500-3000	-1.33	.55+-.15	13.2+-4.1	4iglA	BC Toxin	0.878	1.49	0.106	0.888	278-448	PS50025	Laminin-G Domain				
3000-3315	-2.72	.4+-.14	12.8+-4.2	4d2iA	DNA Translocase	0.672	2.58	0.161	0.721	738-940	SSF049899	Concanavalin A-like lectins/glucanases				
										795-936	SM00282	Laminin-G Domain				
										2921-2940	TMHelix	Transmembrane Helix				
										2966-3210	SSF52540	P-loop containing nucleoside triphosphate hydrolase				
										2971-3237	DUF87	Unknown				

Nanopetramus - AOV94611.1

AA range	Model C-score	TM-Score	RMSD	PDB Homolog	Function	TM-Score	RMSD	Identity	Coverage	AA range	Domain	Function	Number of TMD's	Intracellular AAs	TMD AAs	Extracellular AAs
0-1500	-0.49	0.65+-0.13	11.1+-4.6	4iglA	BC Toxin	0.946	0.84	0.074	0.949	5-27	Tmhelix	Transmembrane Helix	1	1-4	5-27	28-4057
1500-3000	-0.2	.69+-0.12	10.3+-4.6	4iglA	BC Toxin	0.909	1.26	0.097	0.917	60-184	SSF049899	Concanavalin A-like lectins/glucanases				
3000-4057	-1.52	.53+-.15	12.8+-4.2	4O9Y	TcdA1 Toxin	0.92	2.79	0.082	0.959	192-324	SSF69318	Integrin alpha N-terminal Domain				
										399-616	SSF69322	Tricorn Protease domain 2				

AA range	Domain	Function
643-745	SSF69322	Tricorn Protease domain 2
727-838	SSF049899	Concanavalin A-like lectins/glucanases
1309-1389	SSF49265	Fibronectin type III

Nanopetramus - AOV94739.1

AA range	Model C-score	TM-Score	RMSD	PDB Homolog	Function	TM-Score	RMSD	Identity	Coverage	AA range	Domain	Function	Number of TMD's	Intracellular AAs	TMD AAs	Extracellular AAs
0-625	-2.24	0.45+-0.15	13.3+-4.1	5yfpH	Exocyst Complex	0.727	1.68	0.062	0.749	274-532	SSF52540	P-loop containing nucleoside triphosphate hydrolase	0	N/A	N/A	1-625

Lake Tyrrell - LTJ07AB_218510

AA range	Model C-score	TM-Score	RMSD	PDB Homolog	Function	TM-Score	RMSD	Identity	Coverage	AA range	Domain	Function	Number of TMD's	Intracellular AAs	TMD AAs	Extracellular AAs
0-717	-0.86	0.61+-0.14	10.2+-4.6	5yfpB	Exocyst Complex	0.935	1.96	0.071	0.968	374-616	SSF52540	P-loop containing nucleoside triphosphate hydrolase	1	346-717	323-345	1-322

Table S3. Genes used in phylogenetic analysis of SPEARE type III restriction enzyme domains.

Organism	IMG Gene ID	Genbank ID	Annotation	Locus Tag
Ca. Nha. antarcticus	2643306914		NPCBM-associated, NEW3 domain of alpha-galactosidase	NAR1_1133
Ca. Nanohaloarchaea archaeon SG9		AOV95205.1	hypothetical protein	AQV86_04780
Ca. Micrarchaeota archaeon CG1 02 47 40		OIO21255.1	hypothetical protein	AUJ17_02890
Ca. Altiarchaeales archaeon IMC4		ODS42329.1	hypothetical protein	MSIBF_03095
archaeon GW2011 AR3		KHO45953.1	hypothetical protein	QS98_C0006G0018
partial archaeon GW2011 AR13		KHO53209.1	hypothetical protein	QT05_C0007G0001
Ca. Woesearchaeota archaeon CG1 02 33 12		OIO61381.1	hypothetical protein	AUJ83_05050
Ca. Aenigmarchaeota archaeon CG1 02 38 14		OIN86191.1	hypothetical protein	AUJ50_03900
Euryarchaeota archaeon SM23-78		KYK27349.1	hypothetical protein	AYK26_03765
Ca. Micrarchaeota archaeon CG1 02 49 24		OIO25093.1	hypothetical protein	AUJ13_01735
Ca. Nanohaloarchaea archaeon SG9		AOV94740.1	hypothetical protein	AQV86_02335
Ca. Woesearchaeota archaeon CG1 02 33 12		OIO63256.1	hypothetical protein	AUJ83_01515
archaeon GW2011 AR4		KHO45466.1	subtilisin-like protein serine protease	QS99_C0015G0039
archaeon GW2011 AR4		KHO45468.1	hypothetical protein	QS99_C0015G0041
Ca. Nanohaloarchaea archaeon SG9		AOV94739.1	hypothetical protein	AQV86_02330
Lake Tyrrell Nanohaloarchaea	LTJ07AB_218510		Predicted ATPase	LTJ07AB_218510

Table S4. Proteins identified matching to *Ca.* Nha. antarcticus identified from in-solution digestion of proteins.

Gene ID	Locus tag	Annotation
**Glycolysis (Embden-Meyerhof pathway, substrate-level phosphorylation)**		
2643307493	NAR1_11612	glucokinase (GlkA)
2643308091	NAR1_1327	bifunctional ADP-specific glucokinase/phosphofructokinase (PfkC)
2643308087	NAR1_1323	glucose-6-phosphate isomerase (Pgi)
2643308074	NAR1_1310	fructose-bisphosphate aldolase class 1 (FbaB)
2643308090	NAR1_1326	triosephosphate isomerase (Tpi)
2643308079	NAR1_1315	glyceraldehyde-3-phosphate dehydrogenase (Gap)
2643307743	NAR1_12113	phosphoglycerate kinase (Pgk)
2643307293	NAR1_11412	enolase (Eno)
2643307988	NAR1_12358	pyruvate kinase (Pyk)
2643306921	NAR1_1140	pyruvate dehydrogenase E1 component subunit alpha (PdhA)
2643306922	NAR1_1141	pyruvate dehydrogenase E1 component subunit beta (PdhB)
2643306924	NAR1_1143	pyruvate dehydrogenase complex, dihydrolipoyllysine-residue acetyltransferase component (PdhC)
2643306925	NAR1_1144	pyruvate dehydrogenase E3 component/dihydrolipoyl dehydrogenase (PdhD)
2643307858	NAR1_12228	pyruvate dehydrogenase E3 component/dihydrolipoyl dehydrogenase (PdhD)
2643306920	NAR1_1139	acetate-CoA ligase [ADP-forming]
2643306916	NAR1_1135	malate dehydrogenase (oxaloacetate-decarboxylating) (MaeA)
2643307076	NAR1_11195	D-lactate dehydrogenase (LdhA)
**Gluconeogenesis / glycogen biosynthesis**		
2643307780	NAR1_12150	phosphoenolpyruvate synthase (Pps)
2643308073	NAR1_139	fructose-1,6-bisphosphatase class 1 (Fbp)
2643308092	NAR1_1328	phosphomannomutase/phosphoglucomutase
2643307840	NAR1_12210	glucose-1-phosphate adenylyltransferase (GlgC)
2643308088	NAR1_1324	glycogen synthase
2643308089	NAR1_1325	debranching enzyme: six-hairpin glycosidase superfamily / glycogen debranching enzyme, C-terminal domain
**Other carbohydrate metabolism (glycosylation, etc)**		
2643306978	NAR1_1197	glycosyltransferase (AglI homolog)
2643306981	NAR1_11100	UDP-N-acetyl-D-mannosamine dehydrogenase
2643306984	NAR1_11103	hexuronic acid methyltransferase (AglP homolog)
2643306988	NAR1_11107	N-terminal glycosyltransferase subfamily 4-like, N-terminal domain + C-terminal glycosyl transferase, family 1
2643307005	NAR1_11124	NAD-dependent epimerase/dehydratase (UDP-N-acetylglucosamine 4-epimerase WbgU homolog)
2643307001	NAR1_11120	ribbon-helix-helix CopG domain + nucleotidyl transferase domain
2643307004	NAR1_11123	NUDIX hydrolase domain
2643307008	NAR1_11127	oligosaccharyl transferase, STT3 subunit family (dolichyl-diphosphooligosaccharide--protein glycosyltransferase subunit STT3A homolog)
2643307453	NAR1_11572	glycosyltransferase RgtA/B/C/D-like domain; multiple transmembrane helices
2643307758	NAR1_12128	dolichol-phosphate mannosyltransferase
2643307113	NAR1_11232	xylose isomerase-like superfamily
2643307977	NAR1_12347	sugar phosphate isomerase/epimerase: xylose isomerase-like, TIM barrel domain
2643307508	NAR1_11627	nucleotide-diphospho-sugar transferase (GlmU homolog)
2643308084	NAR1_1320	six-hairpin glycosidase-like superfamily (glucoamylase and related glycosyl hydrolase)
2643308082	NAR1_1318	glycoside hydrolase/deacetylase, beta/alpha-barrel superfamily / glycoside hydrolase family 57, N-terminal domain: putative alpha-amylase or glycogen branching enzyme
**Iron / sulfur / phosphorus metabolism**		

2643306987	NAR1_11106	alkaline-phosphatase-like/sulfatase
2643306989	NAR1_11108	alkaline-phosphatase-like/sulfatase
2643307103	NAR1_11222	alkaline phosphatase/sulfatase
2643307310	NAR1_11429	FeS cluster assembly protein SufB
2643307313	NAR1_11432	FeS cluster assembly protein SufD
2643307318	NAR1_11437	FeS cluster assembly ATPase SufC
2643306997	NAR1_11116	sulfate adenylyltransferase subunit 2 (NodP homolog)
2643307263	NAR1_11382	inorganic pyrophosphatase (Ppa)
**Cell surface**		
2643308122	NAR1_1358	signal peptide + S-layer family
2643306914	NAR1_1133	SPEARE protein
2643307034	NAR1_11153	'cell wall protein': signal peptide + immunoglobulin-like fold + C-terminal transmembrane helix
2643307702	NAR1_1272	archaellin/pilin N-terminal-like domain/signal peptide
2643307794	NAR1_12164	archaellin/pilin N-terminal-like domain/signal peptide
2643307098	NAR1_11217	archaellar protein FlaI/Type II/IV secretion system protein (ATPase)
2643307209	NAR1_11328	archaellar protein FlaJ/Type II secretion system F domain
2643307210	NAR1_11329	archaellar protein FlaI/Type II/IV secretion system protein (ATPase)
2643307787	NAR1_12157	archaellar protein FlaJ/Type II secretion system F domain
2643307790	NAR1_12160	archaellar protein FlaI/Type II/IV secretion system protein (ATPase)
2643307183	NAR1_11302	signal peptide + 3x immunoglobulin-like fold + CARDB domain (within 3rd Ig-like fold) + C-terminal transmembrane helix
2643307387	NAR1_11506	immunoglubulin-like fold, C-terminal transmembrane helix
2643307638	NAR1_128	signal peptide + PGF-pre-PGF domain + PKD/chitinase domain + C-terminal transmembrane helix
2643307238	NAR1_11357	signal peptide + concanavalin A-like lectin/glucanase domain superfamily (multiple domains) + galactose-binding-like domain superfamily
2643308046	NAR1_12416	signal peptide + alpha-galactosidase, NEW3 domain + C-terminal transmembrane helix
**Amino acid metabolism**		
2643307459	NAR1_11578	ABC transporter, extracytoplasmic amino acid-binding protein
2643307454	NAR1_11573	asparagine synthetase [glutamine-hydrolyzing] (AsnB)
2643306901	NAR1_1120	T-protein (chorismate mutase/prephenate dehydrogenase) (TyrA)
2643306902	NAR1_1121	aminotransferase class I, broad-specificity (aspartate aminotransferase [AspAT]) (AspC)
2643306903	NAR1_1122	prephenate dehydratase (PheA)
2643307114	NAR1_11233	catabolic NAD-specific glutamate dehydrogenase (RocG homolog)
2643307642	NAR1_1212	serine hydroxymethyltransferase (GlyA)
2643307967	NAR1_12337	S-adenosylmethionine synthase (Mat)
**Efflux/conductance**		
2643306887	NAR1_116	ATP-binding/permease protein (macrolide export MacB homolog)
2643307163	NAR1_11282	CPA2 family cation/H^+ exchanger (KefB homolog)
2643307267	NAR1_11386	ABC transporter ATP-binding protein NatA (sodium efflux)
2643307850	NAR1_12220	calcium-transporting ATPase 1 (PacL)
2643307175	NAR1_11294	mechanosensitive ion channel MscS family
2643307200	NAR1_11319	small-conductance mechanosensitive channel
**Energy conservation & redox metabolism**		
2643307952	NAR1_12322	A-type ATPase/ATP synthase, subunit A (AtpA)
2643307953	NAR1_12323	A-type ATPase/ATP synthase, subunit B (AtpB)
2643307950	NAR1_12320	A-type ATPase/ATP synthase, subunit C (AtpC)
2643307954	NAR1_12324	A-type ATPase/ATP synthase, subunit D (AtpD)
2643307949	NAR1_12319	A-type ATPase/ATP synthase, subunit E (AtpE)
2643307951	NAR1_12321	A-type ATPase/ATP synthase, subunit F (AtpF)

2643307946	NAR1_12316	A-type ATPase/ATP synthase, subunit H (AtpH)
2643307947	NAR1_12317	A-type ATPase/ATP synthase, subunit I (AtpI)
2643307517	NAR1_11636	FAD/NAD(P)-binding domain protein (assimilatory nitrate reductase electron transfer subunit NasB homolog)
2643307855	NAR1_12225	pyridine nucleotide-disulphide oxidoreductase, FAD-dependent (NADH dehydrogenase II Ndh homolog)
2643308033	NAR1_12403	pyridine nucleotide-disulphide oxidoreductase, FAD-dependent (alkyl hydroperoxide large subunit AhpF homolog)
2643307201	NAR1_11320	thioredoxin (TrxA)
**DNA maintenance & replication**		
2643306946	NAR1_1165	archaeal histone A1 (HfoA1)
2643306932	NAR1_1151	archaeal histone A2 (HfoA2)
2643307939	NAR1_12309	archaeal histone B (HfoB)
2643307916	NAR1_12286	minichromosome maintenance protein MCM
2643307651	NAR1_1221	chromosome partition protein Smc
2643306974	NAR1_1193	ORC1-type DNA replication protein
2643306900	NAR1_1119	DNA topoisomerase 1 (TopA)
2643307082	NAR1_11201	DNA topoisomerase 6 subunit B (Top6B)
2643307083	NAR1_11202	DNA topoisomerase 6 subunit A (Top6A)
2643306966	NAR1_1185	DNA primase (DnaG)
2643306972	NAR1_1191	DNA polymerase II large subunit (PolC)
2643306973	NAR1_1192	DNA polymerase II small subunit (PolB)
2643306930	NAR1_1149	DNA-directed DNA polymerase, family B
2643307278	NAR1_11397	DNA polymerase sliding clamp (Pcn)
2643307072	NAR1_11191	flap endonuclease 1 (Fen)
2643307655	NAR1_1225	deoxyuridine 5'-triphosphate nucleotidohydrolase (= dUTP pyrophosphatase) (Dut)
2643307203	NAR1_11322	replication factor C small subunit (RfcS)
2643307926	NAR1_12296	DNA ligase (Lig)
**Transcription**		
2643307942	NAR1_12312	transcription initiation factor IIB (TFIIB)
2643307919	NAR1_12289	TATA-box-binding protein (Tbp)
2643307720	NAR1_1290	DNA-directed RNA polymerase, subunit A' (RpoA1)
2643307719	NAR1_1289	DNA-directed RNA polymerase, subunit A'' (RpoA2)
2643307721	NAR1_1291	DNA-directed RNA polymerase, subunit B' (RpoB1)
2643307722	NAR1_1292	DNA-directed RNA polymerase, subunit B'' (RpoB2)
2643307658	NAR1_1228	DNA-directed RNA polymerase, subunit D (RpoD)
2643307170	NAR1_11289	DNA-directed RNA polymerase, subunit E' (RpoE1)
2643307382	NAR1_11501	DNA-directed RNA polymerase, subunit F (RpoF)
2643307723	NAR1_1293	DNA-directed RNA polymerase, subunit H (RpoH)
2643307271	NAR1_11390	DNA-directed RNA polymerase, subunit L (RpoL)
2643307275	NAR1_11394	DNA-directed RNA polymerase, subunit M (RpoM)
2643307149	NAR1_11268	DNA-directed RNA polymerase, subunit N (RpoN)
2643307421	NAR1_11540	DNA-directed RNA polymerase subunit P (RpoP)
2643306994	NAR1_11113	transcriptional regulator, CopG family
2643306995	NAR1_11114	transcriptional regulator
2643307018	NAR1_11137	winged helix-like DNA-binding domain superfamily (transcriptional regulator TrmB?)
2643307311	NAR1_11430	HTH-type transcriptional regulator Lrp
2643307483	NAR1_11602	Helix-turn-helix, HxlR type; likely transcriptional regulator
2643307495	NAR1_11614	HTH-type transcriptional regulator; Lambda repressor-like, DNA-binding domain superfamily / Cro/C1-type helix-turn-helix domain
2643307928	NAR1_12298	HTH-type sugar sensing transcriptional regulator
**DNA repair**		

2643307865	NAR1_12235	excinuclease ABC subunit B
2643307866	NAR1_12236	excinuclease ABC subunit A
2643306927	NAR1_1146	N-glycosylase/DNA lyase (Ogg)
2643306948	NAR1_1167	DNA repair and recombination protein RadA
2643307159	NAR1_11278	single-stranded-DNA-specific exonuclease RecJ
2643307812	NAR1_12182	DHH phosphoesterase superfamily: single-stranded-DNA-specific exonuclease RecJ
2643307197	NAR1_11316	ATP dependent helicase, Lhr family
2643307397	NAR1_11516	DNA helicase: P-loop containing nucleoside triphosphate hydrolase / helicase HerA family
2643307181	NAR1_11300	deoxyribodipyrimidine photo-lyase (Phr)
2643307269	NAR1_11388	putative DNA helicase Rad25
2643307306	NAR1_11425	ATP-dependent DNA helicase
2643307589	NAR1_11708	DEAD-box ATP-dependent RNA helicase (CshA)
2643307753	NAR1_12123	DNA double-strand break repair helicase HerA
2643307612	NAR1_11731	endonuclease IV
2643307399	NAR1_11518	calcineurin-like phosphoesterase domain, ApaH type (double-strand break repair protein MRE11 homolog)
**Nucleotide metabolism**		
2643307935	NAR1_12305	adenylate kinase (Adk)
2643307283	NAR1_11402	uridylate kinase (PyrH)
2643307385	NAR1_11504	CTP synthase (PyrG)
2643307706	NAR1_1276	cytidylate kinase (Cmk)
2643307413	NAR1_11532	5'-deoxyadenosine deaminase (DadD)
2643307640	NAR1_1210	thymidylate synthase (ThyA)
2643307751	NAR1_12121	nucleoside diphosphate kinase (Ndk)
2643308093	NAR1_1329	diadenosine tetraphosphate (AP-4-A) phosphorylase
**Nucleic acid degradation**		
2643307423	NAR1_11542	exosome complex component Rrp42
2643307424	NAR1_11543	exosome complex component Rrp41
2643307425	NAR1_11544	exosome complex component Rrp4
2643307248	NAR1_11367	ribonuclease J (Rnj)
2643307734	NAR1_12104	ribonuclease HII (RnhB)
2643307017	NAR1_11136	NTP pyrophosphohydrolase MazG, putative catalytic core domain
**Oxidative stress**		
2643307523	NAR1_11642	superoxide dismutase [Mn] (Sod)
**Cell division**		
2643307417	NAR1_11536	archaeal cell division protein (SepF homolog)
2643308010	NAR1_12380	cell division protein FtsZ
2643308104	NAR1_1340	cell division protein FtsZ
2643308096	NAR1_1332	septum site-determining protein MinD
**Translation**		
2643307693	NAR1_1263	translation initiation factor 1 (aIF-1)
2643307079	NAR1_11198	translation initiation factor 1A (aIF-1A)
2643307912	NAR1_12282	translation initiation factor 2 subunit beta (aIF-2b)
2643307286	NAR1_11405	translation initiation factor 2 subunit alpha (aIF-2a)
2643307176	NAR1_11295	translation initiation factor 2 subunit gamma (aIF-2g)
2643307301	NAR1_11420	translation initiation factor 5B (aIF-5B)
2643307365	NAR1_11484	translation initiation factor 6 (aeIF-6)
2643307710	NAR1_1280	translation elongation factor 1A (aEF-1A) (Tuf)
2643307073	NAR1_11192	translation elongation factor 1B (aEF-1B)

2643307711	NAR1_1281	translation elongation factor 2 (aEF-2)
2643307975	NAR1_12345	peptide chain release factor 1 (aRF1)
2643307026	NAR1_11145	translation release factor aPelota
2643306915	NAR1_1134	methionine aminopeptidase (Map)
2643307012	NAR1_11131	nascent polypeptide associated complex NAC protein (Nac)
2643307513	NAR1_11632	2-(3-amino-3-carboxypropyl) histidine synthase (Dph2)
2643307043	NAR1_11162	diphthine synthase (DphB)
2643306896	NAR1_1115	tyrosyl-tRNA synthetase (TyrS)
2643306898	NAR1_1117	seryl-tRNA synthetase (SerS)
2643307740	NAR1_12110	glycyl-tRNA synthetase (GlyS)
2643307099	NAR1_11218	arginyl-tRNA synthetase (AlaS)
2643307254	NAR1_11373	glutamyl-tRNA synthetase (GltX)
2643307745	NAR1_12115	tryptophanyl-tRNA synthetase (TrpS)
2643307748	NAR1_12118	phenylalanyl-tRNA synthetase, alpha subunit (PheS)
2643307359	NAR1_11478	threonyl-tRNA synthetase (ThrS)
2643307403	NAR1_11522	prolyl-tRNA synthetase (ProS)
2643307491	NAR1_11610	histidyl-tRNA synthetase (HisS)
2643307531	NAR1_11650	lysyl-tRNA synthetase (LysS)
2643307652	NAR1_1222	aspartyl-tRNA synthetase (AspS)
2643307810	NAR1_12180	cysteinyl-tRNA synthetase (CysS)
2643307813	NAR1_12183	isoleucyl-tRNA synthetase (IleS)
2643307978	NAR1_12348	valyl-tRNA synthetase (ValS)
2643307985	NAR1_12355	methionyl-tRNA synthetase (MetS)
2643308098	NAR1_1334	leucyl-tRNA synthetase (LeuS)
2643308118	NAR1_1354	alanyl-tRNA synthetase (AlaS)
2643308011	NAR1_12381	aspartyl-tRNA amidotransferase subunit C (GatC)
2643308018	NAR1_12388	aspartyl-tRNA amidotransferase subunit A (GatA)
2643308020	NAR1_12390	aspartyl-tRNA amidotransferase subunit B (GatB)
2643307228	NAR1_11347	glutamyl-tRNA amidotransferase subunit D (GatD)
2643307223	NAR1_11342	glutamyl-tRNA amidotransferase subunit E (GatE)
2643307704	NAR1_1274	tRNA-splicing ligase (RtcB)
2643307379	NAR1_11498	tRNA pseudouridine synthase Pus10
2643307756	NAR1_12126	pseudouridine synthase (TruD)
2643307705	NAR1_1275	archease
2643307832	NAR1_12202	tRNA (cytosine(49)-C(5))-methyltransferase
2643307843	NAR1_12213	tRNA thiolation protein, TtcA/Ctu1 type, family
2643307936	NAR1_12306	CCA-adding enzyme (Cca)
2643306999	NAR1_11118	formyltransferase, N-terminal domain
2643307162	NAR1_11281	KEOPS complex subunit Bud32
2643307356	NAR1_11475	ribonuclease Z (Rnz)
2643307322	NAR1_11441	fibrillarin-like rRNA/tRNA 2'-O-methyltransferase (FlpA)
2643307384	NAR1_11503	ribosomal RNA small subunit methyltransferase (RsmA)
2643307173	NAR1_11292	ribosomal protein S6--L-glutamate ligase (RimK)
2643307150	NAR1_11269	SSU ribosomal protein S9P
2643307151	NAR1_11270	LSU ribosomal protein L13P
2643307152	NAR1_11271	LSU ribosomal protein L18AE
2643307158	NAR1_11277	SSU ribosomal protein S3AE
2643307160	NAR1_11279	SSU ribosomal protein S15P
2643307167	NAR1_11286	SSU ribosomal protein S27AE
2643307168	NAR1_11287	ribosomal protein S24e
2643307177	NAR1_11296	SSU ribosomal protein S6e
2643307284	NAR1_11403	LSU ribosomal protein L44e
2643307294	NAR1_11413	SSU ribosomal protein S2P
2643307298	NAR1_11417	LSU ribosomal protein L7AE

2643307299	NAR1_11418	SSU ribosomal protein S28E
2643307362	NAR1_11481	SSU ribosomal protein S19E
2643307364	NAR1_11483	ribosomal protein L31E
2643307381	NAR1_11500	LSU ribosomal protein L21e
2643307368	NAR1_11487	ribosomal L18ae/LX protein domain
2643307422	NAR1_11541	LSU ribosomal protein L37Ae
2643307511	NAR1_11630	LSU ribosomal protein L10AE
2643307659	NAR1_1229	SSU ribosomal protein S11P
2643307660	NAR1_1230	SSU ribosomal protein S4P
2643307661	NAR1_1231	SSU ribosomal protein S13P
2643307675	NAR1_1245	LSU ribosomal protein L15P
2643307676	NAR1_1246	LSU ribosomal protein L30
2643307677	NAR1_1247	SSU ribosomal protein S5
2643307678	NAR1_1248	LSU ribosomal protein L18P
2643307679	NAR1_1249	LSU ribosomal protein L19E
2643307680	NAR1_1250	LSU ribosomal protein L32e
2643307681	NAR1_1251	LSU ribosomal protein L6P
2643307682	NAR1_1252	SSU ribosomal protein S8
2643307683	NAR1_1253	SSU ribosomal protein S14
2643307684	NAR1_1254	LSU ribosomal protein L5P
2643307685	NAR1_1255	SSU ribosomal protein S4E
2643307686	NAR1_1256	ribosomal protein uL24
2643307687	NAR1_1257	LSU ribosomal protein L14P
2643307688	NAR1_1258	SSU ribosomal protein S17P
2643307695	NAR1_1265	SSU ribosomal protein S3
2643307696	NAR1_1266	LSU ribosomal protein L22
2643307697	NAR1_1267	SSU ribosomal protein S19P
2643307698	NAR1_1268	LSU ribosomal protein L2P
2643307699	NAR1_1269	LSU ribosomal protein L23P
2643307700	NAR1_1270	LSU ribosomal protein L4P
2643307701	NAR1_1271	LSU ribosomal protein L3P
2643307708	NAR1_1278	SSU ribosomal protein S10P
2643307713	NAR1_1283	SSU ribosomal protein S7P
2643307714	NAR1_1284	SSU ribosomal protein S12P
2643307900	NAR1_12270	SSU ribosomal protein S8e
2643307999	NAR1_12369	SSU ribosomal protein S17E
2643308109	NAR1_1345	LSU ribosomal protein L24A
2643308110	NAR1_1346	LSU ribosomal protein L11P
2643308111	NAR1_1347	LSU ribosomal protein L1P
2643308112	NAR1_1348	LSU ribosomal protein L10P
2643308116	NAR1_1352	LSU ribosomal protein L12AE
2643307429	NAR1_11548	ribosome maturation protein SDO1 homolog
2643307662	NAR1_1232	foldase (PrsA)
2643308101	NAR1_1337	FKBP-type peptidyl-prolyl cis-trans isomerase (SlyD)
2643307369	NAR1_11488	prefoldin alpha subunit (PfdA)
2643307419	NAR1_11538	prefoldin beta subunit (PfdB)
2643306926	NAR1_1145	protein-disulfide isomerase (disulfide bond formation protein D BdbD homolog)
2643307247	NAR1_11366	protein-disulfide isomerase (disulfide bond formation protein D BdbD homolog)
**Protein secretion/translocation**		
2643307010	NAR1_11129	protein-export membrane protein SecD
2643307674	NAR1_1244	protein translocase subunit SecY
2643307370	NAR1_11489	signal recognition particle-docking protein FtsY
2643307376	NAR1_11495	signal recognition particle subunit Srp54

**Protein chaperones**		
2643306935	NAR1_1154	chaperone protein GrpE
2643306936	NAR1_1155	chaperone protein DnaK
2643307820	NAR1_12190	chaperone protein DnaK
2643307217	NAR1_11336	thermosome subunit Ths
**Proteolysis**		
2643306953	NAR1_1172	tetrahedral aminopeptidase
2643306954	NAR1_1173	oligoendopeptidase F
2643306959	NAR1_1178	leucyl aminopeptidase (aminopeptidase T)
2643307361	NAR1_11480	leucyl aminopeptidase (aminopeptidase T)
2643307122	NAR1_11241	carboxypeptidase Taq metallopeptidase family
2643307025	NAR1_11144	serine endoprotease (DegP homolog) (signal peptide)
2643308025	NAR1_12395	proteasome-activating nucleotidase (Pan)
2643307430	NAR1_11549	proteasome subunit alpha (PsmA)
**Metabolism – other**		
2643307100	NAR1_11219	agmatinase (SpeB)
2643307460	NAR1_11579	(5-formylfuran-3-yl) methyl phosphate synthase (MfnB)
2643308078	NAR1_1314	nicotinamide-nucleotide adenylyltransferase (NadM)
**General function prediction only - DNA- or RNA-binding**		
2643307081	NAR1_11200	KH domain protein, archaea
2643307604	NAR1_11723	KH and PIN-domain containing protein
2643308008	NAR1_12378	KH domain, NusA-like
2643307415	NAR1_11534	KH-domain/beta-lactamase-domain protein, archaea, family (incl. Beta-Casp + Zn-dependent metallo-hydrolase, RNA specificity domain)
2643307171	NAR1_11290	PIN-like domain superfamily
2643307054	NAR1_11173	Hint domain + Homing endonuclease + P-loop containing nucleoside triphosphate hydrolase; also multiple intein domains, + intein N-terminal splicing region + intein C-terminal splicing region
2643307799	NAR1_12169	Hint domain + Homing endonuclease + P-loop containing nucleoside triphosphate hydrolase; also multiple intein domains, + intein N-terminal splicing region + intein C-terminal splicing region
2643307277	NAR1_11396	OB-fold nucleic acid binding domain, AA-tRNA synthetase-type domain
2643307914	NAR1_12284	OB-fold nucleic acid binding domain, AA-tRNA synthetase-type, domain + winged helix-like DNA-binding domain superfamily
2643307905	NAR1_12275	nucleic acid-binding, OB-fold, domain
2643307817	NAR1_12187	C-terminal Nucleic acid-binding, OB-fold / TRAM domain
2643307791	NAR1_12161	winged helix DNA-binding domain superfamily
2643307477	NAR1_11596	histone-fold
2643307500	NAR1_11619	histone-like protein
2643307070	NAR1_11189	chromosome maintenance protein-like
2643307053	NAR1_11172	chromosome segregation protein-like
2643307071	NAR1_11190	chromosome segregation protein-like
2643307499	NAR1_11618	DUF4349
2643307184	NAR1_11303	lambda repressor-like, DNA-binding domain superfamily / Cro/C1-type helix-turn-helix domain
2643307305	NAR1_11424	ribbon-helix-helix / antitoxin ParD1/3/4
2643307174	NAR1_11293	ATP-grasp fold, RimK-type domain
2643307323	NAR1_11442	NOP5 family
2643307609	NAR1_11728	NYN domain, limkain-b1-type domain
2643307383	NAR1_11502	DUF655 family (putative nucleotide binding)
2643307672	NAR1_1242	tRNA pseudouridine synthase B family

2643307537	NAR1_11656	exonuclease, phage-type/RecB, C-terminal/PD-(D/E)XK endonuclease-like domain, AddAB-type
2643307804	NAR1_12174	ribonuclease Z/hydroxyacylglutathione hydrolase-like / metallo-beta-lactamase
2643307819	NAR1_12189	tRNA sulfurtransferase ThiI family: THUMP domain + Thil, AANH domain
2643307990	NAR1_12360	NurA (nuclease) domain
2643306996	NAR1_11115	alkaline phosphatase-like/Type I phosphodiesterase/nucleotide pyrophosphatase/phosphate transferase
**General function prediction only - proteolysis**		
2643307930	NAR1_12300	signal peptide + ClpP/crotonase-like domain superfamily / Peptidase S49 domain
2643307530	NAR1_11649	membrane-bound transcription factor site-2 protease/Peptidase M50 domain; multiple transmembrane helices
2643307287	NAR1_11406	proteasome assembly chaperone family
2643308083	NAR1_1319	alpha/beta hydrolase fold / serine aminopeptidase, S33
**General function prediction only - metabolic?**		
2643307989	NAR1_12359	haloacid dehydrogenase (HAD) superfamily: phosphoglycolate phosphatase?
2643307504	NAR1_11623	haloacid dehydrogenase (HAD) superfamily
2643307134	NAR1_11253	haloacid dehydrogenase (HAD)-like superfamily
2643307024	NAR1_11143	alkyl hydroperoxide reductase subunit C/thiol specific antioxidant domain + galactose-binding-like domain superfamily
2643308075	NAR1_1311	alkyl hydroperoxide reductase subunit C/thiol specific antioxidant domain + galactose-binding-like domain superfamily
2643307087	NAR1_11206	aldo/keto reductase family
2643307601	NAR1_11720	vitamin B12-dependent ribonucleoside-diphosphate reductase
2643307218	NAR1_11337	AMP-dependent synthetase/ligase domain (phenylacetate-coenzyme A ligase PaaK homolog)
2643307222	NAR1_11341	isochorismatase-like domain (nicotinamidase/pyrazinamidase PncA homolog)
2643307295	NAR1_11414	NAD(P)H-dependent FMN reductase
2643307336	NAR1_11455	kynurenine formamidase/cyclase-like family (kynurenine formamidase KynB homolog)
2643307484	NAR1_11603	thioredoxin / glutaredoxin domain
2643308120	NAR1_1356	thioredoxin-like superfamily, N-terminal transmembrane domain
2643307887	NAR1_12257	thioredoxin-like superfamily / glutathione S-transferase, N-terminal domain
2643307512	NAR1_11631	pyruvate phosphate dikinase, PEP/pyruvate-binding, domain
2643307744	NAR1_12114	succinylglutamate desuccinylase / aspartoacylase family protein
2643307760	NAR1_12130	N-terminal transmembrane helix + thioredoxin-like superfamily
2643307825	NAR1_12195	multiple N-terminal transmembrane helices + SAM-dependent methyltransferase/polyamine biosynthesis domain (polyamine aminopropyltransferase SpeE homolog)
2643307917	NAR1_12287	pterin-4-alpha-carbinolamine dehydratase domain
**General function prediction only - other**		
2643306909	NAR1_1128	AAA ATPase, CDC48 family
2643306971	NAR1_1190	CBS domain-containing protein (2 x CBS domains)
2643307608	NAR1_11727	CBS domain-containing protein (3 x CBS domains)
2643306931	NAR1_1150	calcineurin-like phosphoesterase domain, lpxH type
2643307439	NAR1_11558	calcineurin-like phosphoesterase domain, lpxH type
2643307020	NAR1_11139	PRC barrel domain protein
2643307097	NAR1_11216	RecA-superfamily ATPase, KaiC/GvpD/RAD55 family
2643307109	NAR1_11228	RecA-superfamily ATPase, KaiC/GvpD/RAD55 family
2643307292	NAR1_11411	RecA-superfamily ATPase, KaiC/GvpD/RAD55 family
2643307192	NAR1_11311	P-loop containing nucleoside triphosphate hydrolase superfamily / ATPase, AAA-3 domain (MoxR homolog)
2643307771	NAR1_12141	P-loop containing nucleoside triphosphate hydrolase superfamily / ATPase, AAA-3 domain (MoxR homolog)

2643307883	NAR1_12253	P-loop containing nucleoside triphosphate hydrolase superfamily / SMCs flexible hinge superfamily
2643307885	NAR1_12255	P-loop containing nucleoside triphosphate hydrolase superfamily / Small GTP-binding protein domain
2643308005	NAR1_12375	P-loop containing nucleoside triphosphate hydrolase superfamily / ABC transporter-like domain
2643307617	NAR1_11736	amphi-Trp domain
2643307881	NAR1_12251	amphi-Trp domain
2643308023	NAR1_12393	amphi-Trp domain
2643307107	NAR1_11226	GAF domain
2643307140	NAR1_11259	DUF304 (PH domain-like structure)
2643307290	NAR1_11409	NO signalling/Golgi transport ligand-binding domain superfamily, incl. C-terminal 4-vinyl reductase (4VR) domain
2643307572	NAR1_11691	pheromone shutdown, TraB, bacterial/archaeal domain
2643307488	NAR1_11607	GTP-binding conserved hypothetical protein TIGR00650; translation-associated GTPase
2643307489	NAR1_11608	DUF4186
2643307561	NAR1_11680	stomatin homolog
2643307648	NAR1_1218	ribosomal protein S13-like, H2TH + DUF814
2643307715	NAR1_1285	protein kinase-like domain superfamily / UbiB domain, C-terminal transmembrane helix
2643307762	NAR1_12132	uncharacterised protein family MJ0538
2643307769	NAR1_12139	von Willebrand factor A-like domain superfamily; multiple transmembrane helices
2643307802	NAR1_12172	pheromone shutdown, TraB, family
2643307884	NAR1_12254	DUF2073
2643307899	NAR1_12269	LSM domain, eukaryotic/archaea-type
2643307931	NAR1_12301	elongator complex protein 3-like; radical SAM, alpha/beta horseshoe + acyl-CoA N-acyltransferase
2643307958	NAR1_12328	polymerase, nucleotidyl transferase domain
2643307974	NAR1_12344	Tubby-like, C-terminal superfamily
2643307993	NAR1_12363	SsuA/THI5-like domain
2643306890	NAR1_119	HSP20-like chaperone superfamily
2643308114	NAR1_1350	DUF1704
2643307391	NAR1_11510	N-terminal transmembrane helix + DUF1512
2643307236	NAR1_11355	collagen triple helix repeat
2643307904	NAR1_12274	ribosome interacting GTPase-like
2643306947	NAR1_1166	DUF2240
**No functional prediction**		
2643306886	NAR1_115	signal peptide + C-terminal transmembrane helix
2643306910	NAR1_1129	-
2643306934	NAR1_1153	-
2643306958	NAR1_1177	-
2643306963	NAR1_1182	-
2643306970	NAR1_1189	-
2643307027	NAR1_11146	-
2643307028	NAR1_11147	-
2643307030	NAR1_11149	signal peptide
2643307031	NAR1_11150	signal peptide
2643307032	NAR1_11151	signal peptide
2643307041	NAR1_11160	-
2643307047	NAR1_11166	-
2643307052	NAR1_11171	-
2643307056	NAR1_11175	-
2643307058	NAR1_11177	-
2643307088	NAR1_11207	-
2643307117	NAR1_11236	signal peptide

2643307142	NAR1_11261	-
2643307154	NAR1_11273	-
2643307186	NAR1_11305	-
2643307194	NAR1_11313	signal peptide
2643307212	NAR1_11331	-
2643307215	NAR1_11334	-
2643307255	NAR1_11374	-
2643307258	NAR1_11377	-
2643307276	NAR1_11395	-
2643307282	NAR1_11401	-
2643307296	NAR1_11415	-
2643307297	NAR1_11416	-
2643307302	NAR1_11421	-
2643307324	NAR1_11443	-
2643307372	NAR1_11491	-
2643307373	NAR1_11492	-
2643307392	NAR1_11511	-
2643307393	NAR1_11512	TAT signal
2643307394	NAR1_11513	-
2643307395	NAR1_11514	-
2643307396	NAR1_11515	-
2643307485	NAR1_11604	-
2643307492	NAR1_11611	-
2643307502	NAR1_11621	-
2643307418	NAR1_11537	UPF0147
2643307524	NAR1_11643	-
2643307525	NAR1_11644	-
2643307597	NAR1_11716	UPF0058
2643307571	NAR1_11690	-
2643307611	NAR1_11730	TAT signal
2643307656	NAR1_1226	-
2643307716	NAR1_1286	-
2643307768	NAR1_12138	-
2643307772	NAR1_12142	-
2643307773	NAR1_12143	-
2643307777	NAR1_12147	-
2643307784	NAR1_12154	-
2643307793	NAR1_12163	-
2643307874	NAR1_12244	-
2643307877	NAR1_12247	-
2643307878	NAR1_12248	-
2643307879	NAR1_12249	-
2643307880	NAR1_12250	-
2643307822	NAR1_12192	-
2643307909	NAR1_12279	-
2643307892	NAR1_12262	-
2643307961	NAR1_12331	-
2643307962	NAR1_12332	-
2643307963	NAR1_12333	-
2643307997	NAR1_12367	-
2643308077	NAR1_1313	-
2643308081	NAR1_1317	-
2643308100	NAR1_1336	-
2643308117	NAR1_1353	-

2643308121	NAR1_1357	-
2643308123	NAR1_1359	-

Table S5. Genes present within Nha-Ce contigs, Nha-CHl MAG, and Nha-R1 MAG, identified from read recruitment plots of metagenome data. Regions of high or low coverage were identified by aligning raw reads from environmental metagenomes to Nha-Ce contig Ga0309993_1030. Genes within these regions were then manually reviewed to assign function. [1]A-G are from Fig. S20-S21, denoting regions of high or low coverage.

[1]*SI Appendix*, Fig. S20 labels, Assemblies containing genes	IMG gene ID, size (aa)	Best blast hit	E-Value	AA identity	Functional domains
**A&D, Nha-Ce & Nha-R1**	Ga0309993_1030504, 273	MULTISPECIES: IS5-like element ISHla2 family transposase [Halorubraceae]	0	100	COG3039 (Transposase); pfam01609 (Transposase)
**B, Nha-CHl**	2791619057, 588	ATP-binding protein [Halopiger aswanensis]	0.00E+00	64	N/A
	2791619058, 75	uncharacterized protein Hqrw_1092 [Haloquadratum walsbyi C23]	2.00E-34	73	cd13905 (cupredoxin)
	2791619059, 243	MarR family transcriptional regulator [Halogeometricum borinquense]	2.00E-93	59	N/A
	2791619060, 93	hypothetical protein [Halorubrum aethiopicum]	7.00E-25	60	N/A
	2791619061, 144	hypothetical protein [Halorubrum aethiopicum]	1.00E-63	67	N/A
	2791619062, 83	hypothetical protein C5B89_18960 [Haloferax sp. Atlit-47N]	7.00E-06	39	TIGR01273 (arginine decarboxylase)
	2791619063, 261	MULTISPECIES: hypothetical protein [Haloarcula]	2.00E-148	79	cd05400 (Nucleotidyltransferase)
	2791619064, 214	SLATT domain-containing protein [Haloferax sp. SB3]	1.00E-131	87	N/A
	2791619065, 89	hypothetical protein AKJ36_00110 [candidate division MSBL1 archaeon SCGC-AAA259I07]	4.00E-35	64	N/A
	2791619066, 471	DUF2813 domain-containing protein [Halopiger aswanensis]	0.00E+00	63	pfam13175 (AAA ATPase); COG3593 (ATP-dependent endonuclease); PRK00064 (recombination protein F); cd03240 (ATP-binding cassette)
	2791619067, 43	DUF2813 domain-containing protein [Halopiger aswanensis]	3.00E-05	70	N/A
	2791619068, 81	DUF2813 domain-containing protein [Halopiger aswanensis]	7.00E-12	38	N/A

**B, Nha-R1**	2643307445, 1345	HNH endonuclease [Natrialba taiwanensis]	0.00E+00	70	cd00085 (HNH nuclease); pfam13391 (HNH endonuclease); TIGR02168 (SMC)
	2643307446, 171	hypothetical protein [Halogeometricum borinquense]	2.00E-200	85	N/A
	2643307447, 569	hypothetical protein [Halogeometricum borinquense]	0.00E+00	88	COG1196 (Chromosome Segregation ATPase); TIGR02168 (SMC); PRK03918 (Chromosome Segregation protein); pfam10186 (Vacuolar sorting 38); cd03240 (ATP-binding cassette); smart00787 (SPC7 kinetochore protein); cd13769 (apolipophorin-III)
	2643307448, 149	hypothetical protein [Halogeometricum borinquense]	5.00E-92	88	N/A
	2643307449, 477	DUF932 domain-containing protein [Haloplanus salinus]	7.00E-188	67	TIGR03299 (Phage/plasmid-like protein)
	2643307450, 375	site-specific integrase [Haloplanus salinus]	0.00E+00	85	cd00397 (DNA breaking-rejoining enzymes); pfam00589 (Phage integrase); COG4974 (Site-specific recombinase); TIGR02224 (Tyrosine recombinase); PRK00236 (Tyrosine Recombinase)
**C**	264307543; NAR1_11662, 1210	hypothetical protein [Haloarculaceae archaeon HArcel1]	0	36	N/A
	264307544; NAR1_11663, 187	DUF1788 domain-containing protein [Halosimplex carlsbadense]	3.00E-50	43	N/A
	2643307545; NAR1_11664, 1212	hypothetical protein [Halopiger xanaduensis]	0	34	N/A
	2643307546; NAR1_11665, 1400	BREX-5 system adenine-specific DNA-methyltransferase PglX [Halobacterium sp. CBA113	0	50	pfam02384 (N-6 DNA Methylase)
	2643307547; NAR1_11666, 693	BREX-5 system phosphatase PglZ [Natrinema salaciae]	1.00E-127	33	TIGR02687; pfam08665 (PgIZ domain)
	2643307548; NAR1_11667, 267	helicase [Natronorubrum tibetense]	6.00E-92	60	PRK04914 (Helicase); smart00487 (Helicase); COG0553 (Helicase); pfam04851 (Type III restriction enzyme)
	2643307549; NAR1_11668, 890	hypothetical protein BRC29_03640 [Nanohaloarchaea archaeon SW_7_43_1]	0.00E+00	56	N/A
**E, Nha-CHl & Nha-R1**	2791619978, 303	transposase [Halohasta litchfieldiae]	0	99	COG0675 (Transposase); pfam07282 (Putative transposase); TIGR01766 (transposase); PHA02942 (putative transposase)

**F, Nha-CHl**	2791619143, 386	hypothetical protein [Haloarcula sinaiiensis]	1.00E-41	42	N/A
	2791619144, 244	N/A	N/A	N/A	N/A
	2791619145, 147	N/A	N/A	N/A	N/A
	2791619146, 286	N/A	N/A	N/A	N/A
	2791619147, 77	N/A	N/A	N/A	N/A
	2791619148, 69	N/A	N/A	N/A	N/A
	2791619149, 29	N/A	N/A	N/A	N/A
	2791619150, 239	hypothetical protein [Haloarcula argentinensis]	7.00E-15	27	N/A
**F, Nha-R1**	2643307393, 368	hypothetical protein [Haloarcula sinaiiensis]	3.00E-55	49	pfam10518 (Twin-arginine translocation signal)
	2643307394, 855	hypothetical protein BRD20_11840 [Halobacteriales archaeon SW_8_65_20]	0.003	23	N/A
	2643307395, 386	hypothetical protein [Haloarcula sinaiiensis]	8.00E-43	42	N/A
**G, Nha-Ce & Nha-R1**	Ga0309993_143673, 419	transposase [Haloferax prahovense]	0	95	COG0675 (Transposase); pfam01385 (Probable Transposase); TIGR01766 (Transposase)

Table S6. Primers used for PCR and FISH.

PCR	Target	Name	Forward sequence (5'–3')	Reverse sequence (5'–3')	Reference
	Ca. Nha. antarcticus	SB1	CGAGATTAACAGCGCTCAGG	CGTCCGATCTACCTTCTGTC	This study
	Ca. Nha. antarcticus	SB2	CGCTCAGGCAGTTGCAGTAA	CGTCCGATCTACCTTCTGTCTT	This study
	Ca. Nha. antarcticus	NHA-FISH	CTAGTCGACTGGAGTCTTGA	GTGTATCCCAGAGCATTCG	This study
	Hrr. lacusprofundi	Lac16S	GCCGATTAGGTAGACGGTGG	TCGTGTAACGAGCCTCATCC	This study
	Natrinema	Nat16S	ATACCGTTCAACGCCTGGAG	GGCAGTCCTCCTAATGTAGCC	This study
	Archaea	Archaeal 16S	TTCCGGTTGATCCTGCCCGA	GGTTACCTTGTTACGACTT	41
	Bacteria	Bacterial 16S	AGAGTTTGATCCTGGCTCAG	GGTTACCTTGTTACGACTT	41
**FISH**	**Target**	**Name**[1]	**Sequence (5'–3')**	**Conjugate**	**Reference**
	Ca. Nha. antarcticus	NHAA	GTGTATCCCAGAGCATTCG	Cy5	This study
	Ca. Nha. antarcticus	NHAB	AGCATTCGGGCCATACTGA	Cy5	This study
	Hrr. lacusprofundi	LACA	TTATTACAGTCGACGCTGGTGAGATGTCCG	Cy3	This study
	Hrr. lacusprofundi	LACB	TTTCGGAGTTGGAACCCTTTGTC	Cy3	This study

[1] Two probes were designed for each target organism and each proved successful. For all microscopy images reported here, the 'A' probe for each target was used.

Supplementary Information References

1. D. Nichols, M. R. Miller, N. W. Davies, A. Goodchild, M. Raftery, R. Cavicchioli. Cold adaptation in the Antarctic archaeon, *Methanococcoides burtonii*, involves membrane lipid unsaturation. *J. Bacteriol.* **186**, 8508-8515 (2004).
2. C. Dörr, M. Zaparty, B. Tjaden, H., Brinkmann, B. Siebers B. The hexokinase of the hyperthermophile *Thermoproteus tenax*. ATP-dependent hexokinases and ADP-dependent glucokinases, two alternatives for glucose phosphorylation in Archaea. *J. Biol. Chem.* **278**, 18744-18753 (2003).
3. C. J. Castelle, J. F. Banfield. Major new microbial groups expand diversity and alter our understanding of the tree of life. *Cell* **172**, 1181–119 (2018).
4. J. L. Spudich, O. A. Sineshchekov, E. G. Govorunova. Mechanism divergence in microbial rhodopsins. *Biochim. Biophys. Acta.* **1837**, 546-552 (2014).
5. Y. Liao, T. J. Williams, J. C. Walsh, M. Ji, A. Poljak, P. M. G. Curmi, I. G. Duggin, R. Cavicchioli. Developing a genetic manipulation system for the Antarctic archaeon, *Halorubrum lacusprofundi:* Investigating acetamidase gene function. *Sci. Rep.* **6,** 34639 (2016).
6. D. T. Jones, W. R. Taylor, J. M. Thornton. The rapid generation of mutation data matrices from protein sequences. *Bioinformatics* **8**, 275–282 (1992).
7. S. Kumar, G. Stecher, K. Tamura. MEGA7: Molecular Evolutionary Genetics Analysis Version 7.0 for bigger datasets. *Mol. Biol. Evol.* **33,** 1870-1874 (2016).
8. B. Tschitschko, S. Erdmann, M. Z. DeMaere, S. Roux, P. Panwar, M. A. Allen, T. J. Williams, S. Brazendale, A. M. Hancock, E. A. Eloe-Fadrosh, R. Cavicchioli. Genomic variation and biogeography of Antarctic haloarchaea. *Microbiome* **6**, 113 (2018).
9. M. Richter, R. Rosselló-Móra, F. O. Glöckner, J. Peplies. JSpeciesWS: a web server for prokaryotic species circumscription based on pairwise genome comparison. *Bioinformatics* **32**, 929–931 (2016).
